# Supplementary material for: Genetically predicted on-statin LDL response is associated with higher intracerebral hemorrhage risk
Source: Brain. Author manuscript; Available in PMC 2023 May 30. (PMC9612789; doi:10.1093/brain/awac186)
Supplement: Supplementary material [file EMS175065-supplement-Supplementary_material.pdf]

# Supplementary Methods

## Preparation of the UK Biobank primary care data

A detailed description of the primary care data including limitations is available from the UK Biobank (https://biobank.ndph.ox.ac.uk/showcase/showcase/docs/primary_care_data.pdf). We extracted data on-statin prescriptions from the GP prescriptions table by using international nonproprietary (INN) names, former and current trade names, Dictionary of Medicines and Devices (DM+D) and British National Formulary (BNF) codes of all available ever approved statins. The list of trade names was obtained by queries to the DM+D product search browser (https://services.nhsbsa.nhs.uk/dmd-browser/search). The list of terms and codes used for searching the prescription data is given in **Supplementary Table S1**. We used only prescriptions for dates after 1990, which accounted for the vast majority (99.99%) of the data and also coincided with the time period when wide usage of statins began.[1] The dosages were extracted from the prescription texts by using regular expressions with subsequent manual curation of entries (**Supplementary Table S1**). As quality control measures, we removed duplicate prescriptions for the same data per individual and in case of multiple different statins prescribed for the same date (0.4%), we randomly removed all but one prescription. We defined statin users as individuals who had at least one statin prescription between 1990 and end of follow-up. For obtaining statin exposure metrics, we harmonized the dosages of different statins based on comparison factors from trials evaluating statin efficacy.[2-5] We averaged the slightly different comparison factors to assign potency equivalency factors of 20, 2, 0.5, 0.25, 0.125, for cerivastatin, rosuvastatin, simvastatin, pravastatin, and fluvastatin, respectively, using atorvastatin as the reference statin.[2-5] We then calculated a cumulative statin exposure in units of 10mg atorvastatin/5 years.

Beyond statin prescriptions, we extracted LDL cholesterol measurements from the primary care data. We gathered all available codes for LDL measurements by an automatic search for "LDL" in the lookup table of GP codes and following curation of the code list, we used these codes to extract LDL measurements from the GP clinical event records table (**Supplementary Table S2**). Only two of the codes specified that LDL was calculated (14.8% of the values obtained), for the rest of the codes it was unknown whether LDL was directly measured or calculated. Information on measurement assay was unavailable for all codes. Values were converted from mmol/l to mg/dl by multiplying with the factor 38.67.[6] As quality control measures, we removed entries with measurement date before 1990 or after 2022 (0.003%), entries with empty values (3.6%), entries with values over 300 mg/dl (10.3 mmol/l) or less than 10 mg/dl (0.26 mmol/l) to account for miscoding in mg/dl (0.04%), and duplicate entries.

## Supplementary References


1. O'Keeffe AG, Nazareth I, Petersen I. Time trends in the prescription of statins for the primary prevention of cardiovascular disease in the United Kingdom: a cohort study using The Health Improvement Network primary care data. *Clin Epidemiol*. 2016;8:123-32. doi:10.2147/CLEP.S104258
2. Weng TC, Yang YH, Lin SJ, Tai SH. A systematic review and meta-analysis on the therapeutic equivalence of statins. *J Clin Pharm Ther*. Apr 2010;35(2):139-51. doi:10.1111/j.1365-2710.2009.01085.x
3. Adams SP, Tiellet N, Alaeiilkhchi N, Wright JM. Cerivastatin for lowering lipids. *Cochrane Database Syst Rev*. Jan 25 2020;1:CD012501. doi:10.1002/14651858.CD012501.pub2
4. Grundy SM, Stone NJ, Bailey AL, et al. 2018 AHA/ACC/AACVPR/AAPA/ABC/ACPM/ADA/AGS/APhA/ASPC/NLA/PCNA Guideline on the Management of Blood Cholesterol: A Report of the American College of Cardiology/American Heart Association Task Force on Clinical Practice Guidelines. *Circulation*. Jun 18 2019;139(25):e1082-e1143. doi:10.1161/CIR.0000000000000625
5. Stein EA. Extending therapy options in treating lipid disorders: a clinical review of cerivastatin, a novel HMG-CoA reductase inhibitor. *Drugs*. 1998;56 Suppl 1:25-31; discussion 33. doi:10.2165/00003495-199856001-00004
6. Rugge B, Balshem H, Sehgal R, Relevo R, Gorman P, Helfand M. Screening and treatment of subclinical hypothyroidism or hyperthyroidism. 2012;

**Supplemental Table S1. Terms and codes used for extraction of statin prescriptions and dosages**

| drug_names | read2_codes | bnf_codes | |
|---|---|---|---|
| Cerivastatin | ^bxi | ^0212000B0 | Regex used for extraction of dosages: "(?i)(\\d+)\\s?(mg\|microgram\|mcg)" |
| Atorvastatin | ^bxj | ^0212000C0 | |
| Simvastatin | ^bxg | ^0212000M0 | |
| Fluvastatin | ^bxe | ^0212000X0 | |
| Pravastatin | ^bxk | ^0212000AA | |
| Rosuvastatin | ^bxd | ^0212000Y0 | |
| Eptastatin | | ^0212000AC | |
| Velastatin | | | |
| Pitavastatin | | | |
| Lovastatin | | | |
| Cadaff | | | |
| Lipobay | | | |
| Lescol | | | |
| Lipitor | | | |
| Inegy | | | |
| Lipostat | | | |
| Zocor | | | |
| Crestor | | | |
| Simvador | | | |
| Ranzolont | | | |
| Cholesterol Lowering | | | |
| Dorisin | | | |
| Luvinsta | | | |
| Nandovar | | | |
| Pinmactil | | | |
| Stefluvin | | | |
| Mevacor | | | |
| Pravachol | | | |

**Supplemental Table S2. Codes used for extraction of LDL with entry counts before and after quality control (QC).**

| Code | Description | before QC | after QC |
|---|---|---|---|
| 44d4. | Plasma random LDL cholesterol level | 51 | 36 |
| 44d5. | Plasma fasting LDL cholesterol level | 367 | 278 |
| 44dB. | Plasma LDL cholesterol level | 860 | 830 |
| 44P6. | Serum LDL cholesterol level | 693152 | 673806 |
| 44PD. | Serum fasting LDL cholesterol level | 724 | 502 |
| 44PE. | Serum random LDL cholesterol level | 263 | 232 |
| 44PI. | Calculated LDL cholesterol level | 47401 | 40896 |
| 44R4. | Lipoprotein electroph. - LDL | 5288 | 3900 |
| X772N | LDL - Low density lipoprotein cholesterol level | 225 | 0 |
| X80Ne | LDL - Low density lipoprotein | 23 | 0 |
| X80Ni | LDL - Low density lipoprotein cholesterol | 10 | 0 |
| XaEVs | Plasma LDL cholesterol level | 9193 | 8208 |
| XaIp4 | Calculated LDL cholesterol level | 80193 | 78788 |
| .44P6 | Serum LDL cholesterol level | 0 | 0 |
| .44PD | Serum fasting LDL cholesterol level | 0 | 0 |
| .44PE | Serum random LDL cholesterol level | 0 | 0 |
| .44PI | Calculated LDL cholesterol level | 0 | 0 |
| .44R4 | Lipoprotein electroph. - LDL | 0 | 0 |
| .44d4 | Plasma random LDL cholesterol level | 0 | 0 |
| .44d5 | Plasma fasting LDL cholesterol level | 0 | 0 |
| .44dB | Plasma LDL cholesterol level | 0 | 0 |

**Supplemental Table S3. Distribution of prescribed drugs among ever statin users. We removed 70,410 (1.6%) entries for quality control.**

| Drug | Prescriptions (n) | Percentage | Participants (n) | Percentage |
|---|---|---|---|---|
| Overall statin prescriptions | 4,151,471 | 100.00% | 75,973 | 100.00% |
| Simvastatin | 2,522,810 | 60.77% | 60,334 | 79.42% |
| Atorvastatin | 1,262,694 | 30.42% | 40,789 | 53.69% |
| Pravastatin | 183,546 | 4.42% | 7,399 | 9.74% |
| Rosuvastatin | 159,393 | 3.84% | 5,023 | 6.61% |
| Fluvastatin | 19,892 | 0.48% | 859 | 1.13% |
| Cerivastatin | 3,136 | 0.08% | 344 | 0.45% |
| More than one drug | | | 30,957 | 40.75% |

**Supplemental Table S4. Effect of age, sex, genetic score, statin dose on LDL levels from GP data in linear mixed effects models (clustered by participant id)**

Random effects:

| Groups | Name | Variance | Std.Dev. |
|---|---|---|---|
| eid | (Intercept) | 723.8 | 26.90 |
| Residual | | 733.2 | 27.08 |

Number of obs: 373824, groups: eid, 45664

| **Fixed effects:** | **Estimate** | **Std. Error** | **df** | **t value** | **Pr(>\|t\|)** | **lower .95** | **upper .95** |
|---|---|---|---|---|---|---|---|
| (Intercept) | 1.68E+02 | 1.65E+00 | 4.58E+04 | 102.057 | < 2e-16 | | |
| Age | -6.06E-01 | 2.14E-02 | 4.59E+04 | -28.401 | < 2e-16 | -0.65 | -0.56 |
| Sex | -1.17E+01 | 2.75E-01 | 4.51E+04 | -42.591 | < 2e-16 | -12.26 | -11.18 |
| Statin Dose (1 SD) | -1.88E+01 | 6.51E-02 | 3.72E+05 | -288.607 | < 2e-16 | -18.91 | -18.66 |
| Statin Response Genetic Score (1 SD) | -2.29E+00 | 1.51E-01 | 6.20E+04 | -15.217 | < 2e-16 | -2.59 | -2.00 |
| Years offset | -1.34E+00 | 1.43E-02 | 3.68E+05 | -93.802 | < 2e-16 | -1.37 | -1.32 |
| PC1 | -2.63E-02 | 3.76E-03 | 4.38E+04 | -6.982 | 2.96E-12 | -0.03 | -0.02 |
| PC2 | 2.78E-02 | 5.86E-03 | 4.36E+04 | 4.738 | 2.17E-06 | 0.02 | 0.04 |
| PC3 | -3.67E-02 | 9.53E-03 | 4.33E+04 | -3.851 | 0.00012 | -0.06 | -0.02 |
| PC4 | -8.93E-02 | 1.25E-02 | 4.33E+04 | -7.155 | 8.48E-13 | -0.11 | -0.06 |
| PC5 | -2.11E-01 | 1.88E-02 | 4.55E+04 | -11.251 | < 2e-16 | -0.25 | -0.17 |
| PC6 | -5.64E-03 | 2.89E-02 | 4.44E+04 | -0.195 | 0.8455 | -0.06 | 0.05 |
| PC7 | -2.03E-02 | 2.68E-02 | 4.38E+04 | -0.759 | 0.44768 | -0.07 | 0.03 |
| PC8 | -5.54E-02 | 2.94E-02 | 4.45E+04 | -1.884 | 0.05963 | -0.11 | 0.00 |
| PC9 | -9.85E-02 | 2.66E-02 | 4.42E+04 | -3.697 | 0.00022 | -0.15 | -0.05 |
| PC10 | 8.69E-02 | 2.91E-02 | 4.32E+04 | 2.987 | 0.00282 | 0.03 | 0.14 |
| race | -2.55E+00 | 6.63E-01 | 4.49E+04 | -3.838 | 0.00012 | -3.84 | -1.25 |
| kinship | -4.48E-03 | 2.71E-01 | 4.53E+04 | -0.017 | 0.9868 | -0.53 | 0.53 |
| gen_assay | 9.88E-01 | 4.34E-01 | 4.54E+04 | 2.279 | 0.02269 | 0.14 | 1.84 |
| Statin Response Genetic Score : Years Offset | -4.51E-02 | 1.24E-02 | 3.56E+05 | -3.633 | 0.00028 | -0.07 | -0.02 |

**Supplemental Table S5. SNPs associated with LDL response after statin intake which were used for the genetic score and the adjusted genetic score**

| | | | Association with LDL response after statin intake (from Smit et al. 2020) | | | Association with baseline LDL among non-statin users in the UKB | | | | |
|---|---|---|---|---|---|---|---|---|---|---|
| rsid | Effect allele | Other allele | Beta | SE | pval | Beta [mg/dl] | SE | pval | Included in the score | Included in the alternative score |
| rs10026358 | G | T | 5 | 1.1 | 2.8E-06 | 0.205 | 0.187 | 2.73E-01 | Yes | Yes |
| rs10145152 | G | A | 5.9 | 1.4 | 1.4E-05 | -0.062 | 0.288 | 8.28E-01 | Yes | Yes |
| rs10455872 | A | G | 5.2 | 0.4 | 7.4E-44 | -3.762 | 0.139 | 3.65E-160 | Yes | No |
| rs1080022 | T | C | 2.4 | 0.6 | 3.0E-05 | -0.204 | 0.161 | 2.05E-01 | Yes | Yes |
| rs10961118 | A | G | 6.7 | 1.5 | 7.5E-06 | -0.064 | 0.318 | 8.41E-01 | Yes | Yes |
| rs10994219 | C | T | 8.5 | 2.1 | 4.5E-05 | 0.475 | 0.305 | 1.19E-01 | Yes | Yes |
| rs11144515 | C | A | 1.7 | 0.4 | 1.8E-05 | 0.151 | 0.132 | 2.51E-01 | Yes | Yes |
| rs1152493 | C | G | 1.8 | 0.4 | 1.2E-05 | -0.057 | 0.121 | 6.39E-01 | Yes | Yes |
| rs11638450 | T | C | 1.2 | 0.2 | 3.2E-07 | -0.257 | 0.093 | 5.74E-03 | Yes | Yes |
| rs11708473 | T | C | 1.2 | 0.3 | 3.5E-05 | -0.095 | 0.082 | 2.43E-01 | Yes | Yes |
| rs12428035 | T | C | 1.3 | 0.3 | 8.4E-07 | 0.241 | 0.116 | 3.83E-02 | Yes | Yes |
| rs1399708 | C | T | 1.1 | 0.3 | 2.9E-05 | 0.122 | 0.105 | 2.45E-01 | Yes | Yes |
| rs16848588 | A | C | 9.8 | 2.4 | 4.1E-05 | -0.051 | 0.212 | 8.09E-01 | Yes | Yes |
| rs17236593 | C | G | 1.6 | 0.4 | 3.8E-05 | 0.040 | 0.104 | 7.03E-01 | Yes | Yes |
| rs17343443 | A | G | 2 | 0.5 | 3.9E-05 | 0.226 | 0.134 | 9.07E-02 | Yes | Yes |
| rs17756944 | T | C | 2.9 | 0.7 | 1.6E-05 | -0.022 | 0.192 | 9.08E-01 | Yes | Yes |
| rs2891833 | C | T | 1.1 | 0.3 | 1.1E-05 | 0.081 | 0.085 | 3.43E-01 | Yes | Yes |
| rs2900478 | T | A | 1.6 | 0.3 | 1.2E-09 | 0.427 | 0.100 | 1.89E-05 | Yes | No |
| rs2939334 | C | G | 2.3 | 0.5 | 1.3E-05 | 0.200 | 0.178 | 2.59E-01 | Yes | Yes |
| rs32506 | T | C | 4.2 | 0.9 | 3.2E-06 | -0.304 | 0.213 | 1.54E-01 | Yes | Yes |
| rs3797135 | C | G | 2 | 0.5 | 1.7E-05 | 0.213 | 0.140 | 1.28E-01 | Yes | Yes |

| | | | | | | | | | | |
|---|---|---|---|---|---|---|---|---|---|---|
| rs445925 | A | G | 5.1 | 0.5 | 8.5E-29 | -11.548 | 0.112 | 2.39396E-22[illegible] | Yes | No |
| rs4613264 | G | A | 0.8 | 0.2 | 4.1E-06 | -0.077 | 0.074 | 2.97E-01 | Yes | Yes |
| rs646776 | C | T | 1.3 | 0.2 | 1.1E-09 | -4.698 | 0.086 | 1.50509E-65[illegible] | Yes | No |
| rs6478277 | C | T | 0.9 | 0.2 | 3.3E-06 | 0.036 | 0.078 | 6.50E-01 | Yes | Yes |
| rs6494593 | A | G | 6.9 | 1.7 | 3.4E-05 | 0.318 | 0.262 | 2.24E-01 | Yes | Yes |
| rs6687920 | T | C | 2.9 | 0.7 | 1.2E-05 | -0.099 | 0.160 | 5.37E-01 | Yes | Yes |
| rs6739693 | T | G | 1.7 | 0.4 | 3.5E-06 | 0.026 | 0.133 | 8.47E-01 | Yes | Yes |
| rs6884324 | T | C | 2.2 | 0.5 | 4.7E-05 | -0.181 | 0.119 | 1.28E-01 | Yes | Yes |
| rs6980143 | G | C | 1.6 | 0.4 | 3.1E-05 | 0.116 | 0.074 | 1.14E-01 | Yes | Yes |
| rs7586037 | A | G | 5.1 | 1.2 | 1.8E-05 | -0.174 | 0.284 | 5.40E-01 | Yes | Yes |
| rs7696430 | G | A | 1.4 | 0.3 | 8.4E-08 | -0.048 | 0.099 | 6.25E-01 | Yes | Yes |
| rs7835385 | T | G | 1.9 | 0.4 | 8.5E-06 | 0.085 | 0.120 | 4.76E-01 | Yes | Yes |
| rs9397844 | C | T | 2.7 | 0.6 | 1.8E-06 | -0.141 | 0.140 | 3.13E-01 | Yes | Yes |
| rs981844 | G | A | 0.9 | 0.2 | 9.7E-07 | 0.101 | 0.085 | 2.37E-01 | Yes | Yes |

**Supplemental Table S6. Association of NMR fatty acid metabolites with statin dose, genetic score, and adjusted genetic score**

| collective | metabolite | field | variable | Estimate | Std. Error | t value | p value | p value Bonferroni |
|---|---|---|---|---|---|---|---|---|
| Statin users | Total Cholesterol | 23400 | Statin dose (1 SD) | -0.0591746 | 0.00878175 | -6.74 | 1.72E-11 | 3.93E-09 |
| Statin users | Total Cholesterol | 23400 | GRS (1 SD) | -0.0333302 | 0.00563145 | -5.92 | 3.30E-09 | 7.53E-07 |
| Statin users | Total Cholesterol | 23400 | Adjusted GRS (1 SD) | -0.0020947 | 0.00556378 | -0.38 | 7.07E-01 | 1.00E+00 |
| Non-statin users | Total Cholesterol | 23400 | GRS (1 SD) | -0.0566806 | 0.00282419 | -20.07 | 2.15E-89 | 4.91E-87 |
| Non-statin users | Total Cholesterol | 23400 | Adjusted GRS (1 SD) | 0.00085609 | 0.00281073 | 0.30 | 7.61E-01 | 1.00E+00 |
| All | Total Cholesterol | 23400 | Statin dose (1 SD) | -0.1421819 | 0.0111662 | -12.73 | 8.03E-37 | 1.83E-34 |
| All | Total Cholesterol | 23400 | GRS (1 SD) | -0.0429846 | 0.00267463 | -16.07 | 4.68E-58 | 1.07E-55 |
| All | Total Cholesterol | 23400 | Adjusted GRS (1 SD) | 0.00410048 | 0.00265815 | 1.54 | 1.23E-01 | 1.00E+00 |
| Statin users | Total Cholesterol Minus HDL-C | 23401 | Statin dose (1 SD) | -0.0511513 | 0.00751542 | -6.81 | 1.08E-11 | 2.47E-09 |
| Statin users | Total Cholesterol Minus HDL-C | 23401 | GRS (1 SD) | -0.0290475 | 0.00481282 | -6.04 | 1.61E-09 | 3.68E-07 |
| Statin users | Total Cholesterol Minus HDL-C | 23401 | Adjusted GRS (1 SD) | 0.00073296 | 0.00475517 | 0.15 | 8.78E-01 | 1.00E+00 |
| Non-statin users | Total Cholesterol Minus HDL-C | 23401 | GRS (1 SD) | -0.0555906 | 0.00259503 | -21.42 | 1.52E-101 | 3.46E-99 |
| Non-statin users | Total Cholesterol Minus HDL-C | 23401 | Adjusted GRS (1 SD) | 0.00080923 | 0.00258349 | 0.31 | 7.54E-01 | 1.00E+00 |
| All | Total Cholesterol Minus HDL-C | 23401 | Statin dose (1 SD) | -0.131454 | 0.01004006 | -13.09 | 8.17E-39 | 1.86E-36 |
| All | Total Cholesterol Minus HDL-C | 23401 | GRS (1 SD) | -0.0422518 | 0.0024448 | -17.28 | 7.74E-67 | 1.77E-64 |
| All | Total Cholesterol Minus HDL-C | 23401 | Adjusted GRS (1 SD) | 0.00413494 | 0.00243015 | 1.70 | 8.88E-02 | 1.00E+00 |
| Statin users | Remnant Cholesterol | 23402 | Statin dose (1 SD) | -0.0265864 | 0.00365551 | -7.27 | 3.88E-13 | 8.85E-11 |
| Statin users | Remnant Cholesterol | 23402 | GRS (1 SD) | -0.0098769 | 0.0023394 | -4.22 | 2.43E-05 | 5.55E-03 |
| Statin users | Remnant Cholesterol | 23402 | Adjusted GRS (1 SD) | 0.00099151 | 0.00231026 | 0.43 | 6.68E-01 | 1.00E+00 |
| Non-statin users | Remnant Cholesterol | 23402 | GRS (1 SD) | -0.0233946 | 0.001285 | -18.21 | 6.33E-74 | 1.44E-71 |
| Non-statin users | Remnant Cholesterol | 23402 | Adjusted GRS (1 SD) | 0.00071859 | 0.00127836 | 0.56 | 5.74E-01 | 1.00E+00 |
| All | Remnant Cholesterol | 23402 | Statin dose (1 SD) | -0.0680781 | 0.0049719 | -13.69 | 2.98E-42 | 6.79E-40 |
| All | Remnant Cholesterol | 23402 | GRS (1 SD) | -0.0165393 | 0.001215 | -13.61 | 3.63E-42 | 8.28E-40 |
| All | Remnant Cholesterol | 23402 | Adjusted GRS (1 SD) | 0.0024672 | 0.00120713 | 2.04 | 4.10E-02 | 1.00E+00 |
| Statin users | VLDL Cholesterol | 23403 | Statin dose (1 SD) | -0.0110828 | 0.00229694 | -4.83 | 1.43E-06 | 3.26E-04 |
| Statin users | VLDL Cholesterol | 23403 | GRS (1 SD) | 0.00214732 | 0.00145872 | 1.47 | 1.41E-01 | 1.00E+00 |

| collective | metabolite | field | variable | Estimate | Std. Error | t value | p value | p value Bonferroni |
|---|---|---|---|---|---|---|---|---|
| Statin users | VLDL Cholesterol | 23403 | Adjusted GRS (1 SD) | 0.0016517 | 0.00143992 | 1.15 | 2.51E-01 | 1.00E+00 |
| Non-statin users | VLDL Cholesterol | 23403 | GRS (1 SD) | -0.0056446 | 0.00079305 | -7.12 | 1.11E-12 | 2.52E-10 |
| Non-statin users | VLDL Cholesterol | 23403 | Adjusted GRS (1 SD) | 0.0007527 | 0.00078769 | 0.96 | 3.39E-01 | 1.00E+00 |
| All | VLDL Cholesterol | 23403 | Statin dose (1 SD) | -0.0321669 | 0.00297098 | -10.83 | 3.77E-27 | 8.59E-25 |
| All | VLDL Cholesterol | 23403 | GRS (1 SD) | -0.00226 | 0.00072368 | -3.12 | 1.79E-03 | 4.08E-01 |
| All | VLDL Cholesterol | 23403 | Adjusted GRS (1 SD) | 0.00165881 | 0.00071845 | 2.31 | 2.10E-02 | 1.00E+00 |
| Statin users | Clinical LDL Cholesterol | 23404 | Statin dose (1 SD) | -0.0452831 | 0.00657084 | -6.89 | 5.98E-12 | 1.36E-09 |
| Statin users | Clinical LDL Cholesterol | 23404 | GRS (1 SD) | -0.0382455 | 0.00421297 | -9.08 | 1.21E-19 | 2.76E-17 |
| Statin users | Clinical LDL Cholesterol | 23404 | Adjusted GRS (1 SD) | -0.0017411 | 0.00416742 | -0.42 | 6.76E-01 | 1.00E+00 |
| Non-statin users | Clinical LDL Cholesterol | 23404 | GRS (1 SD) | -0.0586365 | 0.00225644 | -25.99 | 2.58E-148 | 5.87E-146 |
| Non-statin users | Clinical LDL Cholesterol | 23404 | Adjusted GRS (1 SD) | -8.259E-05 | 0.00224915 | -0.04 | 9.71E-01 | 1.00E+00 |
| All | Clinical LDL Cholesterol | 23404 | Statin dose (1 SD) | -0.112487 | 0.00870142 | -12.93 | 6.85E-38 | 1.56E-35 |
| All | Clinical LDL Cholesterol | 23404 | GRS (1 SD) | -0.0474235 | 0.00212774 | -22.29 | 8.15E-110 | 1.86E-107 |
| All | Clinical LDL Cholesterol | 23404 | Adjusted GRS (1 SD) | 0.00254304 | 0.00211679 | 1.20 | 2.30E-01 | 1.00E+00 |
| Statin users | LDL Cholesterol | 23405 | Statin dose (1 SD) | -0.0245646 | 0.00404726 | -6.07 | 1.35E-09 | 3.07E-07 |
| Statin users | LDL Cholesterol | 23405 | GRS (1 SD) | -0.0191704 | 0.00259201 | -7.40 | 1.46E-13 | 3.33E-11 |
| Statin users | LDL Cholesterol | 23405 | Adjusted GRS (1 SD) | -0.0002582 | 0.00256217 | -0.10 | 9.20E-01 | 1.00E+00 |
| Non-statin users | LDL Cholesterol | 23405 | GRS (1 SD) | -0.032196 | 0.00136389 | -23.61 | 8.08E-123 | 1.84E-120 |
| Non-statin users | LDL Cholesterol | 23405 | Adjusted GRS (1 SD) | 9.0554E-05 | 0.00135858 | 0.07 | 9.47E-01 | 1.00E+00 |
| All | LDL Cholesterol | 23405 | Statin dose (1 SD) | -0.0633754 | 0.0052499 | -12.07 | 2.69E-33 | 6.14E-31 |
| All | LDL Cholesterol | 23405 | GRS (1 SD) | -0.0257126 | 0.00127325 | -20.19 | 1.56E-90 | 3.56E-88 |
| All | LDL Cholesterol | 23405 | Adjusted GRS (1 SD) | 0.00166777 | 0.00126621 | 1.32 | 1.88E-01 | 1.00E+00 |
| Statin users | HDL Cholesterol | 23406 | Statin dose (1 SD) | -0.0080238 | 0.00328456 | -2.44 | 1.46E-02 | 1.00E+00 |
| Statin users | HDL Cholesterol | 23406 | GRS (1 SD) | -0.0042828 | 0.00208652 | -2.05 | 4.01E-02 | 1.00E+00 |
| Statin users | HDL Cholesterol | 23406 | Adjusted GRS (1 SD) | -0.0028279 | 0.00205971 | -1.37 | 1.70E-01 | 1.00E+00 |
| Non-statin users | HDL Cholesterol | 23406 | GRS (1 SD) | -0.0010898 | 0.00099816 | -1.09 | 2.75E-01 | 1.00E+00 |
| Non-statin users | HDL Cholesterol | 23406 | Adjusted GRS (1 SD) | 4.7088E-05 | 0.00099115 | 0.05 | 9.62E-01 | 1.00E+00 |
| All | HDL Cholesterol | 23406 | Statin dose (1 SD) | -0.0107288 | 0.00352908 | -3.04 | 2.37E-03 | 5.41E-01 |

| collective | metabolite | field | variable | Estimate | Std. Error | t value | p value | p value Bonferroni |
|---|---|---|---|---|---|---|---|---|
| All | HDL Cholesterol | 23406 | GRS (1 SD) | -0.0007327 | 0.00086566 | -0.85 | 3.97E-01 | 1.00E+00 |
| All | HDL Cholesterol | 23406 | Adjusted GRS (1 SD) | -3.434E-05 | 0.00085939 | -0.04 | 9.68E-01 | 1.00E+00 |
| Statin users | Total TG | 23407 | Statin dose (1 SD) | -0.0020202 | 0.00675373 | -0.30 | 7.65E-01 | 1.00E+00 |
| Statin users | Total TG | 23407 | GRS (1 SD) | 0.03523958 | 0.00420858 | 8.37 | 5.98E-17 | 1.36E-14 |
| Statin users | Total TG | 23407 | Adjusted GRS (1 SD) | 0.00684131 | 0.00416149 | 1.64 | 1.00E-01 | 1.00E+00 |
| Non-statin users | Total TG | 23407 | GRS (1 SD) | 0.01575595 | 0.00182678 | 8.62 | 6.52E-18 | 1.49E-15 |
| Non-statin users | Total TG | 23407 | Adjusted GRS (1 SD) | 0.0024839 | 0.00181468 | 1.37 | 1.71E-01 | 1.00E+00 |
| All | Total TG | 23407 | Statin dose (1 SD) | -0.0211452 | 0.00762235 | -2.77 | 5.55E-03 | 1.00E+00 |
| All | Total TG | 23407 | GRS (1 SD) | 0.01950123 | 0.00165299 | 11.80 | 4.19E-32 | 9.55E-30 |
| All | Total TG | 23407 | Adjusted GRS (1 SD) | 0.00347249 | 0.00164195 | 2.11 | 3.44E-02 | 1.00E+00 |
| Statin users | TG in VLDL | 23408 | Statin dose (1 SD) | -0.0038748 | 0.00570627 | -0.68 | 4.97E-01 | 1.00E+00 |
| Statin users | TG in VLDL | 23408 | GRS (1 SD) | 0.03017562 | 0.00354539 | 8.51 | 1.84E-17 | 4.21E-15 |
| Statin users | TG in VLDL | 23408 | Adjusted GRS (1 SD) | 0.00578413 | 0.00350593 | 1.65 | 9.90E-02 | 1.00E+00 |
| Non-statin users | TG in VLDL | 23408 | GRS (1 SD) | 0.01337756 | 0.00152133 | 8.79 | 1.48E-18 | 3.37E-16 |
| Non-statin users | TG in VLDL | 23408 | Adjusted GRS (1 SD) | 0.00207267 | 0.00151127 | 1.37 | 1.70E-01 | 1.00E+00 |
| All | TG in VLDL | 23408 | Statin dose (1 SD) | -0.0190778 | 0.00639872 | -2.98 | 2.88E-03 | 6.56E-01 |
| All | TG in VLDL | 23408 | GRS (1 SD) | 0.01652486 | 0.00137824 | 11.99 | 4.20E-33 | 9.58E-31 |
| All | TG in VLDL | 23408 | Adjusted GRS (1 SD) | 0.00283921 | 0.00136907 | 2.07 | 3.81E-02 | 1.00E+00 |
| Statin users | TG in LDL | 23409 | Statin dose (1 SD) | 0.00094483 | 0.00044422 | 2.13 | 3.35E-02 | 1.00E+00 |
| Statin users | TG in LDL | 23409 | GRS (1 SD) | 0.00115198 | 0.00028274 | 4.07 | 4.63E-05 | 1.06E-02 |
| Statin users | TG in LDL | 23409 | Adjusted GRS (1 SD) | 0.00032197 | 0.0002792 | 1.15 | 2.49E-01 | 1.00E+00 |
| Non-statin users | TG in LDL | 23409 | GRS (1 SD) | 0.00012989 | 0.00013273 | 0.98 | 3.28E-01 | 1.00E+00 |
| Non-statin users | TG in LDL | 23409 | Adjusted GRS (1 SD) | 0.0001533 | 0.0001318 | 1.16 | 2.45E-01 | 1.00E+00 |
| All | TG in LDL | 23409 | Statin dose (1 SD) | -0.0008461 | 0.00052513 | -1.61 | 1.07E-01 | 1.00E+00 |
| All | TG in LDL | 23409 | GRS (1 SD) | 0.00038461 | 0.00011919 | 3.23 | 1.25E-03 | 2.85E-01 |
| All | TG in LDL | 23409 | Adjusted GRS (1 SD) | 0.00022936 | 0.00011833 | 1.94 | 5.26E-02 | 1.00E+00 |
| Statin users | TG in HDL | 23410 | Statin dose (1 SD) | 0.00032052 | 0.0005428 | 0.59 | 5.55E-01 | 1.00E+00 |
| Statin users | TG in HDL | 23410 | GRS (1 SD) | 0.00323543 | 0.00033989 | 9.52 | 1.95E-21 | 4.44E-19 |

| collective | metabolite | field | variable | Estimate | Std. Error | t value | p value | p value Bonferroni |
|---|---|---|---|---|---|---|---|---|
| Statin users | TG in HDL | 23410 | Adjusted GRS (1 SD) | 0.00053315 | 0.00033626 | 1.59 | 1.13E-01 | 1.00E+00 |
| Non-statin users | TG in HDL | 23410 | GRS (1 SD) | 0.00206538 | 0.0001552 | 13.31 | 2.29E-40 | 5.22E-38 |
| Non-statin users | TG in HDL | 23410 | Adjusted GRS (1 SD) | 0.00016163 | 0.00015427 | 1.05 | 2.95E-01 | 1.00E+00 |
| All | TG in HDL | 23410 | Statin dose (1 SD) | -0.0006659 | 0.00061102 | -1.09 | 2.76E-01 | 1.00E+00 |
| All | TG in HDL | 23410 | GRS (1 SD) | 0.00226663 | 0.0001378 | 16.45 | 9.98E-61 | 2.28E-58 |
| All | TG in HDL | 23410 | Adjusted GRS (1 SD) | 0.00025339 | 0.00013696 | 1.85 | 6.43E-02 | 1.00E+00 |
| Statin users | Total Phospholipids in Lipoprotein | 23411 | Statin dose (1 SD) | -0.0243908 | 0.00492595 | -4.95 | 7.53E-07 | 1.72E-04 |
| Statin users | Total Phospholipids in Lipoprotein | 23411 | GRS (1 SD) | -0.0002869 | 0.00312342 | -0.09 | 9.27E-01 | 1.00E+00 |
| Statin users | Total Phospholipids in Lipoprotein | 23411 | Adjusted GRS (1 SD) | -0.0004556 | 0.00308311 | -0.15 | 8.83E-01 | 1.00E+00 |
| Non-statin users | Total Phospholipids in Lipoprotein | 23411 | GRS (1 SD) | -0.0111472 | 0.00143816 | -7.75 | 9.21E-15 | 2.10E-12 |
| Non-statin users | Total Phospholipids in Lipoprotein | 23411 | Adjusted GRS (1 SD) | 0.00113922 | 0.00142852 | 0.80 | 4.25E-01 | 1.00E+00 |
| All | Total Phospholipids in Lipoprotein | 23411 | Statin dose (1 SD) | -0.0571111 | 0.0057667 | -9.90 | 5.27E-23 | 1.20E-20 |
| All | Total Phospholipids in Lipoprotein | 23411 | GRS (1 SD) | -0.0057135 | 0.00132059 | -4.33 | 1.52E-05 | 3.46E-03 |
| All | Total Phospholipids in Lipoprotein | 23411 | Adjusted GRS (1 SD) | 0.00238969 | 0.00131111 | 1.82 | 6.84E-02 | 1.00E+00 |
| Statin users | Phospholipids in VLDL | 23412 | Statin dose (1 SD) | -0.0048492 | 0.00188443 | -2.57 | 1.01E-02 | 1.00E+00 |
| Statin users | Phospholipids in VLDL | 23412 | GRS (1 SD) | 0.00610067 | 0.00118642 | 5.14 | 2.74E-07 | 6.26E-05 |
| Statin users | Phospholipids in VLDL | 23412 | Adjusted GRS (1 SD) | 0.00176546 | 0.00117184 | 1.51 | 1.32E-01 | 1.00E+00 |
| Non-statin users | Phospholipids in VLDL | 23412 | GRS (1 SD) | -0.0001835 | 0.00058366 | -0.31 | 7.53E-01 | 1.00E+00 |
| Non-statin users | Phospholipids in VLDL | 23412 | Adjusted GRS (1 SD) | 0.00068063 | 0.00057955 | 1.17 | 2.40E-01 | 1.00E+00 |
| All | Phospholipids in VLDL | 23412 | Statin dose (1 SD) | -0.0169446 | 0.00226742 | -7.47 | 8.57E-14 | 1.95E-11 |
| All | Phospholipids in VLDL | 23412 | GRS (1 SD) | 0.00185018 | 0.00052588 | 3.52 | 4.34E-04 | 9.91E-02 |
| All | Phospholipids in VLDL | 23412 | Adjusted GRS (1 SD) | 0.00122994 | 0.00052208 | 2.36 | 1.85E-02 | 1.00E+00 |
| Statin users | Phospholipids in LDL | 23413 | Statin dose (1 SD) | -0.0075751 | 0.00128825 | -5.88 | 4.28E-09 | 9.76E-07 |
| Statin users | Phospholipids in LDL | 23413 | GRS (1 SD) | -0.0066954 | 0.00082477 | -8.12 | 5.02E-16 | 1.15E-13 |
| Statin users | Phospholipids in LDL | 23413 | Adjusted GRS (1 SD) | -0.0001426 | 0.00081551 | -0.17 | 8.61E-01 | 1.00E+00 |
| Non-statin users | Phospholipids in LDL | 23413 | GRS (1 SD) | -0.0110286 | 0.00043843 | -25.15 | 3.92E-139 | 8.94E-137 |
| Non-statin users | Phospholipids in LDL | 23413 | Adjusted GRS (1 SD) | 3.6212E-05 | 0.00043691 | 0.08 | 9.34E-01 | 1.00E+00 |
| All | Phospholipids in LDL | 23413 | Statin dose (1 SD) | -0.0202304 | 0.00168476 | -12.01 | 5.77E-33 | 1.32E-30 |

| collective | metabolite | field | variable | Estimate | Std. Error | t value | p value | p value Bonferroni |
|---|---|---|---|---|---|---|---|---|
| All | Phospholipids in LDL | 23413 | GRS (1 SD) | -0.0088925 | 0.00040926 | -21.73 | 1.78E-104 | 4.06E-102 |
| All | Phospholipids in LDL | 23413 | Adjusted GRS (1 SD) | 0.00053871 | 0.00040711 | 1.32 | 1.86E-01 | 1.00E+00 |
| Statin users | Phospholipids in HDL | 23414 | Statin dose (1 SD) | -0.0078909 | 0.00337058 | -2.34 | 1.93E-02 | 1.00E+00 |
| Statin users | Phospholipids in HDL | 23414 | GRS (1 SD) | 0.00341764 | 0.00212387 | 1.61 | 1.08E-01 | 1.00E+00 |
| Statin users | Phospholipids in HDL | 23414 | Adjusted GRS (1 SD) | -0.0020052 | 0.00209655 | -0.96 | 3.39E-01 | 1.00E+00 |
| Non-statin users | Phospholipids in HDL | 23414 | GRS (1 SD) | 0.0047326 | 0.00098292 | 4.81 | 1.48E-06 | 3.36E-04 |
| Non-statin users | Phospholipids in HDL | 23414 | Adjusted GRS (1 SD) | 0.00037339 | 0.00097613 | 0.38 | 7.02E-01 | 1.00E+00 |
| All | Phospholipids in HDL | 23414 | Statin dose (1 SD) | -0.0094185 | 0.00363273 | -2.59 | 9.54E-03 | 1.00E+00 |
| All | Phospholipids in HDL | 23414 | GRS (1 SD) | 0.00498389 | 0.00085402 | 5.84 | 5.37E-09 | 1.22E-06 |
| All | Phospholipids in HDL | 23414 | Adjusted GRS (1 SD) | 0.00030892 | 0.00084795 | 0.36 | 7.16E-01 | 1.00E+00 |
| Statin users | Total Esterified Cholesterol | 23415 | Statin dose (1 SD) | -0.0425335 | 0.00637989 | -6.67 | 2.80E-11 | 6.39E-09 |
| Statin users | Total Esterified Cholesterol | 23415 | GRS (1 SD) | -0.0257508 | 0.0040932 | -6.29 | 3.22E-10 | 7.34E-08 |
| Statin users | Total Esterified Cholesterol | 23415 | Adjusted GRS (1 SD) | -0.0021677 | 0.00404447 | -0.54 | 5.92E-01 | 1.00E+00 |
| Non-statin users | Total Esterified Cholesterol | 23415 | GRS (1 SD) | -0.0416714 | 0.00202952 | -20.53 | 1.83E-93 | 4.16E-91 |
| Non-statin users | Total Esterified Cholesterol | 23415 | Adjusted GRS (1 SD) | 0.00042292 | 0.00202007 | 0.21 | 8.34E-01 | 1.00E+00 |
| All | Total Esterified Cholesterol | 23415 | Statin dose (1 SD) | -0.1003212 | 0.00801069 | -12.52 | 1.10E-35 | 2.51E-33 |
| All | Total Esterified Cholesterol | 23415 | GRS (1 SD) | -0.0320882 | 0.00191753 | -16.73 | 8.75E-63 | 1.99E-60 |
| All | Total Esterified Cholesterol | 23415 | Adjusted GRS (1 SD) | 0.00264649 | 0.0019059 | 1.39 | 1.65E-01 | 1.00E+00 |
| Statin users | CE in VLDL | 23416 | Statin dose (1 SD) | -0.0072577 | 0.00127348 | -5.70 | 1.25E-08 | 2.85E-06 |
| Statin users | CE in VLDL | 23416 | GRS (1 SD) | -0.000456 | 0.00081253 | -0.56 | 5.75E-01 | 1.00E+00 |
| Statin users | CE in VLDL | 23416 | Adjusted GRS (1 SD) | 0.00075617 | 0.00080204 | 0.94 | 3.46E-01 | 1.00E+00 |
| Non-statin users | CE in VLDL | 23416 | GRS (1 SD) | -0.0046209 | 0.00045684 | -10.11 | 4.89E-24 | 1.12E-21 |
| Non-statin users | CE in VLDL | 23416 | Adjusted GRS (1 SD) | 0.00037737 | 0.00045389 | 0.83 | 4.06E-01 | 1.00E+00 |
| All | CE in VLDL | 23416 | Statin dose (1 SD) | -0.0203592 | 0.00170188 | -11.96 | 9.85E-33 | 2.25E-30 |
| All | CE in VLDL | 23416 | GRS (1 SD) | -0.0025521 | 0.00042086 | -6.06 | 1.33E-09 | 3.04E-07 |
| All | CE in VLDL | 23416 | Adjusted GRS (1 SD) | 0.00093462 | 0.00041787 | 2.24 | 2.53E-02 | 1.00E+00 |
| Statin users | CE in LDL | 23417 | Statin dose (1 SD) | -0.017412 | 0.00301072 | -5.78 | 7.62E-09 | 1.74E-06 |
| Statin users | CE in LDL | 23417 | GRS (1 SD) | -0.0118598 | 0.00192536 | -6.16 | 7.43E-10 | 1.69E-07 |

| collective | metabolite | field | variable | Estimate | Std. Error | t value | p value | p value Bonferroni |
|---|---|---|---|---|---|---|---|---|
| Statin users | CE in LDL | 23417 | Adjusted GRS (1 SD) | 0.00014812 | 0.00190237 | 0.08 | 9.38E-01 | 1.00E+00 |
| Non-statin users | CE in LDL | 23417 | GRS (1 SD) | -0.0216763 | 0.00100799 | -21.50 | 2.60E-102 | 5.92E-100 |
| Non-statin users | CE in LDL | 23417 | Adjusted GRS (1 SD) | 0.0001839 | 0.00100353 | 0.18 | 8.55E-01 | 1.00E+00 |
| All | CE in LDL | 23417 | Statin dose (1 SD) | -0.0455986 | 0.00387675 | -11.76 | 1.04E-31 | 2.38E-29 |
| All | CE in LDL | 23417 | GRS (1 SD) | -0.0169971 | 0.00093652 | -18.15 | 1.64E-73 | 3.74E-71 |
| All | CE in LDL | 23417 | Adjusted GRS (1 SD) | 0.00134319 | 0.00093103 | 1.44 | 1.49E-01 | 1.00E+00 |
| Statin users | CE in HDL | 23418 | Statin dose (1 SD) | -0.0062107 | 0.00258349 | -2.40 | 1.62E-02 | 1.00E+00 |
| Statin users | CE in HDL | 23418 | GRS (1 SD) | -0.0034975 | 0.0016416 | -2.13 | 3.31E-02 | 1.00E+00 |
| Statin users | CE in HDL | 23418 | Adjusted GRS (1 SD) | -0.0024108 | 0.00162051 | -1.49 | 1.37E-01 | 1.00E+00 |
| Non-statin users | CE in HDL | 23418 | GRS (1 SD) | -0.0005664 | 0.00078291 | -0.72 | 4.69E-01 | 1.00E+00 |
| Non-statin users | CE in HDL | 23418 | Adjusted GRS (1 SD) | -3.073E-05 | 0.00077741 | -0.04 | 9.68E-01 | 1.00E+00 |
| All | CE in HDL | 23418 | Statin dose (1 SD) | -0.0073828 | 0.00277681 | -2.66 | 7.86E-03 | 1.00E+00 |
| All | CE in HDL | 23418 | GRS (1 SD) | -0.0004271 | 0.00067921 | -0.63 | 5.29E-01 | 1.00E+00 |
| All | CE in HDL | 23418 | Adjusted GRS (1 SD) | -0.0001444 | 0.00067429 | -0.21 | 8.30E-01 | 1.00E+00 |
| Statin users | Total FC | 23419 | Statin dose (1 SD) | -0.0166408 | 0.00246298 | -6.76 | 1.52E-11 | 3.47E-09 |
| Statin users | Total FC | 23419 | GRS (1 SD) | -0.0075793 | 0.0015768 | -4.81 | 1.55E-06 | 3.52E-04 |
| Statin users | Total FC | 23419 | Adjusted GRS (1 SD) | 7.3082E-05 | 0.00155738 | 0.05 | 9.63E-01 | 1.00E+00 |
| Non-statin users | Total FC | 23419 | GRS (1 SD) | -0.0150091 | 0.00081363 | -18.45 | 7.66E-76 | 1.75E-73 |
| Non-statin users | Total FC | 23419 | Adjusted GRS (1 SD) | 0.00043326 | 0.00080947 | 0.54 | 5.92E-01 | 1.00E+00 |
| All | Total FC | 23419 | Statin dose (1 SD) | -0.0418602 | 0.00321836 | -13.01 | 2.48E-38 | 5.66E-36 |
| All | Total FC | 23419 | GRS (1 SD) | -0.0108964 | 0.00077229 | -14.11 | 3.63E-45 | 8.28E-43 |
| All | Total FC | 23419 | Adjusted GRS (1 SD) | 0.00145403 | 0.00076734 | 1.89 | 5.81E-02 | 1.00E+00 |
| Statin users | FC in VLDL | 23420 | Statin dose (1 SD) | -0.0038251 | 0.00107095 | -3.57 | 3.57E-04 | 8.14E-02 |
| Statin users | FC in VLDL | 23420 | GRS (1 SD) | 0.00260333 | 0.00067558 | 3.85 | 1.17E-04 | 2.66E-02 |
| Statin users | FC in VLDL | 23420 | Adjusted GRS (1 SD) | 0.00089554 | 0.00066709 | 1.34 | 1.79E-01 | 1.00E+00 |
| Non-statin users | FC in VLDL | 23420 | GRS (1 SD) | -0.0010238 | 0.00034609 | -2.96 | 3.10E-03 | 7.06E-01 |
| Non-statin users | FC in VLDL | 23420 | Adjusted GRS (1 SD) | 0.00037529 | 0.00034367 | 1.09 | 2.75E-01 | 1.00E+00 |
| All | FC in VLDL | 23420 | Statin dose (1 SD) | -0.0118076 | 0.00132129 | -8.94 | 4.82E-19 | 1.10E-16 |

| collective | metabolite | field | variable | Estimate | Std. Error | t value | p value | p value Bonferroni |
|---|---|---|---|---|---|---|---|---|
| All | FC in VLDL | 23420 | GRS (1 SD) | 0.00029207 | 0.00031285 | 0.93 | 3.51E-01 | 1.00E+00 |
| All | FC in VLDL | 23420 | Adjusted GRS (1 SD) | 0.00072416 | 0.00031058 | 2.33 | 1.97E-02 | 1.00E+00 |
| Statin users | FC in LDL | 23421 | Statin dose (1 SD) | -0.0071525 | 0.00109665 | -6.52 | 7.39E-11 | 1.69E-08 |
| Statin users | FC in LDL | 23421 | GRS (1 SD) | -0.0073107 | 0.0007038 | -10.39 | 3.29E-25 | 7.51E-23 |
| Statin users | FC in LDL | 23421 | Adjusted GRS (1 SD) | -0.0004065 | 0.00069664 | -0.58 | 5.60E-01 | 1.00E+00 |
| Non-statin users | FC in LDL | 23421 | GRS (1 SD) | -0.0105197 | 0.00037068 | -28.38 | 2.27E-176 | 5.17E-174 |
| Non-statin users | FC in LDL | 23421 | Adjusted GRS (1 SD) | -9.341E-05 | 0.00036975 | -0.25 | 8.01E-01 | 1.00E+00 |
| All | FC in LDL | 23421 | Statin dose (1 SD) | -0.0177766 | 0.00143196 | -12.41 | 4.24E-35 | 9.67E-33 |
| All | FC in LDL | 23421 | GRS (1 SD) | -0.0087155 | 0.00034956 | -24.93 | 7.51E-137 | 1.71E-134 |
| All | FC in LDL | 23421 | Adjusted GRS (1 SD) | 0.00032447 | 0.00034795 | 0.93 | 3.51E-01 | 1.00E+00 |
| Statin users | FC in HDL | 23422 | Statin dose (1 SD) | -0.0018132 | 0.00072795 | -2.49 | 1.28E-02 | 1.00E+00 |
| Statin users | FC in HDL | 23422 | GRS (1 SD) | -0.000785 | 0.00046175 | -1.70 | 8.91E-02 | 1.00E+00 |
| Statin users | FC in HDL | 23422 | Adjusted GRS (1 SD) | -0.000417 | 0.00045582 | -0.91 | 3.60E-01 | 1.00E+00 |
| Non-statin users | FC in HDL | 23422 | GRS (1 SD) | -0.0005234 | 0.00022319 | -2.35 | 1.90E-02 | 1.00E+00 |
| Non-statin users | FC in HDL | 23422 | Adjusted GRS (1 SD) | 7.7824E-05 | 0.00022163 | 0.35 | 7.25E-01 | 1.00E+00 |
| All | FC in HDL | 23422 | Statin dose (1 SD) | -0.003346 | 0.00078677 | -4.25 | 2.13E-05 | 4.86E-03 |
| All | FC in HDL | 23422 | GRS (1 SD) | -0.0003056 | 0.00019374 | -1.58 | 1.15E-01 | 1.00E+00 |
| All | FC in HDL | 23422 | Adjusted GRS (1 SD) | 0.00011005 | 0.00019233 | 0.57 | 5.67E-01 | 1.00E+00 |
| Statin users | Total Lipids in Lipoprotein | 23423 | Statin dose (1 SD) | -0.085585 | 0.01694229 | -5.05 | 4.49E-07 | 1.02E-04 |
| Statin users | Total Lipids in Lipoprotein | 23423 | GRS (1 SD) | 0.00162354 | 0.01072976 | 0.15 | 8.80E-01 | 1.00E+00 |
| Statin users | Total Lipids in Lipoprotein | 23423 | Adjusted GRS (1 SD) | 0.00429169 | 0.01059125 | 0.41 | 6.85E-01 | 1.00E+00 |
| Non-statin users | Total Lipids in Lipoprotein | 23423 | GRS (1 SD) | -0.0520722 | 0.00513794 | -10.13 | 3.99E-24 | 9.10E-22 |
| Non-statin users | Total Lipids in Lipoprotein | 23423 | Adjusted GRS (1 SD) | 0.00447903 | 0.00510476 | 0.88 | 3.80E-01 | 1.00E+00 |
| All | Total Lipids in Lipoprotein | 23423 | Statin dose (1 SD) | -0.2204386 | 0.02070444 | -10.65 | 2.58E-26 | 5.89E-24 |
| All | Total Lipids in Lipoprotein | 23423 | GRS (1 SD) | -0.0291969 | 0.00476336 | -6.13 | 8.84E-10 | 2.02E-07 |
| All | Total Lipids in Lipoprotein | 23423 | Adjusted GRS (1 SD) | 0.00996267 | 0.00472951 | 2.11 | 3.52E-02 | 1.00E+00 |
| Statin users | Total Lipids in VLDL | 23424 | Statin dose (1 SD) | -0.0198072 | 0.00945048 | -2.10 | 3.61E-02 | 1.00E+00 |
| Statin users | Total Lipids in VLDL | 23424 | GRS (1 SD) | 0.03842358 | 0.00591118 | 6.50 | 8.22E-11 | 1.87E-08 |

| collective | metabolite | field | variable | Estimate | Std. Error | t value | p value | p value Bonferroni |
|---|---|---|---|---|---|---|---|---|
| Statin users | Total Lipids in VLDL | 23424 | Adjusted GRS (1 SD) | 0.00920118 | 0.00584088 | 1.58 | 1.15E-01 | 1.00E+00 |
| Non-statin users | Total Lipids in VLDL | 23424 | GRS (1 SD) | 0.00754952 | 0.00275957 | 2.74 | 6.23E-03 | 1.00E+00 |
| Non-statin users | Total Lipids in VLDL | 23424 | Adjusted GRS (1 SD) | 0.00350599 | 0.00274025 | 1.28 | 2.01E-01 | 1.00E+00 |
| All | Total Lipids in VLDL | 23424 | Statin dose (1 SD) | -0.0681899 | 0.01102131 | -6.19 | 6.40E-10 | 1.46E-07 |
| All | Total Lipids in VLDL | 23424 | GRS (1 SD) | 0.01611505 | 0.00248585 | 6.48 | 9.04E-11 | 2.06E-08 |
| All | Total Lipids in VLDL | 23424 | Adjusted GRS (1 SD) | 0.00572794 | 0.00246822 | 2.32 | 2.03E-02 | 1.00E+00 |
| Statin users | Total Lipids in LDL | 23425 | Statin dose (1 SD) | -0.0311954 | 0.00554888 | -5.62 | 1.96E-08 | 4.46E-06 |
| Statin users | Total Lipids in LDL | 23425 | GRS (1 SD) | -0.0247138 | 0.00355399 | -6.95 | 3.67E-12 | 8.36E-10 |
| Statin users | Total Lipids in LDL | 23425 | Adjusted GRS (1 SD) | -7.903E-05 | 0.0035125 | -0.02 | 9.82E-01 | 1.00E+00 |
| Non-statin users | Total Lipids in LDL | 23425 | GRS (1 SD) | -0.0430946 | 0.0018707 | -23.04 | 4.46E-117 | 1.02E-114 |
| Non-statin users | Total Lipids in LDL | 23425 | Adjusted GRS (1 SD) | 0.00028023 | 0.00186314 | 0.15 | 8.80E-01 | 1.00E+00 |
| All | Total Lipids in LDL | 23425 | Statin dose (1 SD) | -0.0844525 | 0.00719989 | -11.73 | 1.52E-31 | 3.47E-29 |
| All | Total Lipids in LDL | 23425 | GRS (1 SD) | -0.0342203 | 0.00174199 | -19.64 | 8.88E-86 | 2.02E-83 |
| All | Total Lipids in LDL | 23425 | Adjusted GRS (1 SD) | 0.00243595 | 0.0017322 | 1.41 | 1.60E-01 | 1.00E+00 |
| Statin users | Total Lipids in HDL | 23426 | Statin dose (1 SD) | -0.0155953 | 0.00664724 | -2.35 | 1.90E-02 | 1.00E+00 |
| Statin users | Total Lipids in HDL | 23426 | GRS (1 SD) | 0.00237134 | 0.00419963 | 0.56 | 5.72E-01 | 1.00E+00 |
| Statin users | Total Lipids in HDL | 23426 | Adjusted GRS (1 SD) | -0.0042992 | 0.00414535 | -1.04 | 3.00E-01 | 1.00E+00 |
| Non-statin users | Total Lipids in HDL | 23426 | GRS (1 SD) | 0.00570823 | 0.00196933 | 2.90 | 3.75E-03 | 8.55E-01 |
| Non-statin users | Total Lipids in HDL | 23426 | Adjusted GRS (1 SD) | 0.00058213 | 0.00195557 | 0.30 | 7.66E-01 | 1.00E+00 |
| All | Total Lipids in HDL | 23426 | Statin dose (1 SD) | -0.0208141 | 0.00715276 | -2.91 | 3.62E-03 | 8.26E-01 |
| All | Total Lipids in HDL | 23426 | GRS (1 SD) | 0.00651806 | 0.00170881 | 3.81 | 1.37E-04 | 3.11E-02 |
| All | Total Lipids in HDL | 23426 | Adjusted GRS (1 SD) | 0.0005281 | 0.00169653 | 0.31 | 7.56E-01 | 1.00E+00 |
| Statin users | Total Concentration of Lipoprotein | 23427 | Statin dose (1 SD) | -9.742E-05 | 2.8318E-05 | -3.44 | 5.85E-04 | 1.33E-01 |
| Statin users | Total Concentration of Lipoprotein | 23427 | GRS (1 SD) | -8.599E-06 | 1.7959E-05 | -0.48 | 6.32E-01 | 1.00E+00 |
| Statin users | Total Concentration of Lipoprotein | 23427 | Adjusted GRS (1 SD) | -2.075E-05 | 1.7726E-05 | -1.17 | 2.42E-01 | 1.00E+00 |
| Non-statin users | Total Concentration of Lipoprotein | 23427 | GRS (1 SD) | -2.385E-05 | 7.908E-06 | -3.02 | 2.57E-03 | 5.85E-01 |
| Non-statin users | Total Concentration of Lipoprotein | 23427 | Adjusted GRS (1 SD) | 1.0343E-06 | 7.8528E-06 | 0.13 | 8.95E-01 | 1.00E+00 |
| All | Total Concentration of Lipoprotein | 23427 | Statin dose (1 SD) | -0.0001634 | 3.0668E-05 | -5.33 | 1.01E-07 | 2.31E-05 |

| collective | metabolite | field | variable | Estimate | Std. Error | t value | p value | p value Bonferroni |
|---|---|---|---|---|---|---|---|---|
| All | Total Concentration of Lipoprotein | 23427 | GRS (1 SD) | -1.15E-05 | 7.0097E-06 | -1.64 | 1.01E-01 | 1.00E+00 |
| All | Total Concentration of Lipoprotein | 23427 | Adjusted GRS (1 SD) | 2.5063E-06 | 6.959E-06 | 0.36 | 7.19E-01 | 1.00E+00 |
| Statin users | Concentration of VLDL | 23428 | Statin dose (1 SD) | -1.235E-06 | 4.39E-07 | -2.81 | 4.91E-03 | 1.00E+00 |
| Statin users | Concentration of VLDL | 23428 | GRS (1 SD) | 6.8293E-07 | 2.782E-07 | 2.45 | 1.41E-02 | 1.00E+00 |
| Statin users | Concentration of VLDL | 23428 | Adjusted GRS (1 SD) | 3.7854E-07 | 2.7464E-07 | 1.38 | 1.68E-01 | 1.00E+00 |
| Non-statin users | Concentration of VLDL | 23428 | GRS (1 SD) | -7.387E-07 | 1.4308E-07 | -5.16 | 2.44E-07 | 5.56E-05 |
| Non-statin users | Concentration of VLDL | 23428 | Adjusted GRS (1 SD) | 1.5179E-07 | 1.4209E-07 | 1.07 | 2.85E-01 | 1.00E+00 |
| All | Concentration of VLDL | 23428 | Statin dose (1 SD) | -4.572E-06 | 5.4572E-07 | -8.38 | 6.20E-17 | 1.41E-14 |
| All | Concentration of VLDL | 23428 | GRS (1 SD) | -2.025E-07 | 1.2936E-07 | -1.57 | 1.17E-01 | 1.00E+00 |
| All | Concentration of VLDL | 23428 | Adjusted GRS (1 SD) | 3.0516E-07 | 1.2842E-07 | 2.38 | 1.75E-02 | 1.00E+00 |
| Statin users | Concentration of LDL | 23429 | Statin dose (1 SD) | -1.397E-05 | 2.5284E-06 | -5.53 | 3.39E-08 | 7.72E-06 |
| Statin users | Concentration of LDL | 23429 | GRS (1 SD) | -9.045E-06 | 1.6162E-06 | -5.60 | 2.22E-08 | 5.06E-06 |
| Statin users | Concentration of LDL | 23429 | Adjusted GRS (1 SD) | 6.6176E-07 | 1.5967E-06 | 0.41 | 6.79E-01 | 1.00E+00 |
| Non-statin users | Concentration of LDL | 23429 | GRS (1 SD) | -1.755E-05 | 8.8122E-07 | -19.91 | 4.79E-88 | 1.09E-85 |
| Non-statin users | Concentration of LDL | 23429 | Adjusted GRS (1 SD) | 4.4156E-07 | 8.7699E-07 | 0.50 | 6.15E-01 | 1.00E+00 |
| All | Concentration of LDL | 23429 | Statin dose (1 SD) | -4.084E-05 | 3.4053E-06 | -11.99 | 6.97E-33 | 1.59E-30 |
| All | Concentration of LDL | 23429 | GRS (1 SD) | -1.327E-05 | 8.2509E-07 | -16.08 | 3.89E-58 | 8.87E-56 |
| All | Concentration of LDL | 23429 | Adjusted GRS (1 SD) | 1.5057E-06 | 8.2001E-07 | 1.84 | 6.63E-02 | 1.00E+00 |
| Statin users | Concentration of HDL | 23430 | Statin dose (1 SD) | -7.714E-05 | 2.7354E-05 | -2.82 | 4.82E-03 | 1.00E+00 |
| Statin users | Concentration of HDL | 23430 | GRS (1 SD) | 3.4442E-06 | 1.7322E-05 | 0.20 | 8.42E-01 | 1.00E+00 |
| Statin users | Concentration of HDL | 23430 | Adjusted GRS (1 SD) | -2.181E-05 | 1.7098E-05 | -1.28 | 2.02E-01 | 1.00E+00 |
| Non-statin users | Concentration of HDL | 23430 | GRS (1 SD) | 3.8953E-07 | 7.6258E-06 | 0.05 | 9.59E-01 | 1.00E+00 |
| Non-statin users | Concentration of HDL | 23430 | Adjusted GRS (1 SD) | 4.446E-07 | 7.5722E-06 | 0.06 | 9.53E-01 | 1.00E+00 |
| All | Concentration of HDL | 23430 | Statin dose (1 SD) | -0.0001054 | 2.9392E-05 | -3.59 | 3.38E-04 | 7.71E-02 |
| All | Concentration of HDL | 23430 | GRS (1 SD) | 6.6506E-06 | 6.7097E-06 | 0.99 | 3.22E-01 | 1.00E+00 |
| All | Concentration of HDL | 23430 | Adjusted GRS (1 SD) | 3.6262E-07 | 6.6611E-06 | 0.05 | 9.57E-01 | 1.00E+00 |
| Statin users | Avg. Diameter for VLDL | 23431 | Statin dose (1 SD) | -0.049304 | 0.01623931 | -3.04 | 2.41E-03 | 5.48E-01 |
| Statin users | Avg. Diameter for VLDL | 23431 | GRS (1 SD) | 0.11012965 | 0.01007132 | 10.93 | 9.45E-28 | 2.16E-25 |

| collective | metabolite | field | variable | Estimate | Std. Error | t value | p value | p value Bonferroni |
|---|---|---|---|---|---|---|---|---|
| Statin users | Avg. Diameter for VLDL | 23431 | Adjusted GRS (1 SD) | 0.01748207 | 0.0099712 | 1.75 | 7.96E-02 | 1.00E+00 |
| Non-statin users | Avg. Diameter for VLDL | 23431 | GRS (1 SD) | 0.04547082 | 0.00382755 | 11.88 | 1.60E-32 | 3.64E-30 |
| Non-statin users | Avg. Diameter for VLDL | 23431 | Adjusted GRS (1 SD) | 0.00323183 | 0.00380366 | 0.85 | 3.96E-01 | 1.00E+00 |
| All | Avg. Diameter for VLDL | 23431 | Statin dose (1 SD) | -0.0632301 | 0.01699595 | -3.72 | 2.00E-04 | 4.56E-02 |
| All | Avg. Diameter for VLDL | 23431 | GRS (1 SD) | 0.05437982 | 0.00347713 | 15.64 | 4.47E-55 | 1.02E-52 |
| All | Avg. Diameter for VLDL | 23431 | Adjusted GRS (1 SD) | 0.0048131 | 0.00345551 | 1.39 | 1.64E-01 | 1.00E+00 |
| Statin users | Avg. Diameter for LDL | 23432 | Statin dose (1 SD) | -0.0002396 | 0.00103948 | -0.23 | 8.18E-01 | 1.00E+00 |
| Statin users | Avg. Diameter for LDL | 23432 | GRS (1 SD) | -0.0051225 | 0.00064903 | -7.89 | 3.11E-15 | 7.10E-13 |
| Statin users | Avg. Diameter for LDL | 23432 | Adjusted GRS (1 SD) | -0.0005278 | 0.00064167 | -0.82 | 4.11E-01 | 1.00E+00 |
| Non-statin users | Avg. Diameter for LDL | 23432 | GRS (1 SD) | -0.0038134 | 0.00028377 | -13.44 | 3.95E-41 | 9.00E-39 |
| Non-statin users | Avg. Diameter for LDL | 23432 | Adjusted GRS (1 SD) | 5.7344E-05 | 0.00028206 | 0.20 | 8.39E-01 | 1.00E+00 |
| All | Avg. Diameter for LDL | 23432 | Statin dose (1 SD) | -0.0024971 | 0.0011261 | -2.22 | 2.66E-02 | 1.00E+00 |
| All | Avg. Diameter for LDL | 23432 | GRS (1 SD) | -0.0037077 | 0.00025227 | -14.70 | 7.39E-49 | 1.69E-46 |
| All | Avg. Diameter for LDL | 23432 | Adjusted GRS (1 SD) | 4.0739E-05 | 0.00025067 | 0.16 | 8.71E-01 | 1.00E+00 |
| Statin users | Avg. Diameter for HDL | 23433 | Statin dose (1 SD) | -0.003766 | 0.00203388 | -1.85 | 6.41E-02 | 1.00E+00 |
| Statin users | Avg. Diameter for HDL | 23433 | GRS (1 SD) | -0.001546 | 0.00128219 | -1.21 | 2.28E-01 | 1.00E+00 |
| Statin users | Avg. Diameter for HDL | 23433 | Adjusted GRS (1 SD) | -0.0008757 | 0.00126567 | -0.69 | 4.89E-01 | 1.00E+00 |
| Non-statin users | Avg. Diameter for HDL | 23433 | GRS (1 SD) | 0.00139727 | 0.00063743 | 2.19 | 2.84E-02 | 1.00E+00 |
| Non-statin users | Avg. Diameter for HDL | 23433 | Adjusted GRS (1 SD) | 0.00011216 | 0.00063296 | 0.18 | 8.59E-01 | 1.00E+00 |
| All | Avg. Diameter for HDL | 23433 | Statin dose (1 SD) | -0.0056868 | 0.00218204 | -2.61 | 9.17E-03 | 1.00E+00 |
| All | Avg. Diameter for HDL | 23433 | GRS (1 SD) | 0.00144596 | 0.00054981 | 2.63 | 8.54E-03 | 1.00E+00 |
| All | Avg. Diameter for HDL | 23433 | Adjusted GRS (1 SD) | 8.9526E-05 | 0.00054584 | 0.16 | 8.70E-01 | 1.00E+00 |
| Statin users | Phosphoglycerides | 23434 | Statin dose (1 SD) | -0.0170288 | 0.00436898 | -3.90 | 9.80E-05 | 2.23E-02 |
| Statin users | Phosphoglycerides | 23434 | GRS (1 SD) | 0.00398638 | 0.00275401 | 1.45 | 1.48E-01 | 1.00E+00 |
| Statin users | Phosphoglycerides | 23434 | Adjusted GRS (1 SD) | -0.0004688 | 0.00271853 | -0.17 | 8.63E-01 | 1.00E+00 |
| Non-statin users | Phosphoglycerides | 23434 | GRS (1 SD) | -0.0039251 | 0.00123549 | -3.18 | 1.49E-03 | 3.39E-01 |
| Non-statin users | Phosphoglycerides | 23434 | Adjusted GRS (1 SD) | 0.00093922 | 0.00122684 | 0.77 | 4.44E-01 | 1.00E+00 |
| All | Phosphoglycerides | 23434 | Statin dose (1 SD) | -0.0372031 | 0.00490848 | -7.58 | 3.82E-14 | 8.70E-12 |

| collective | metabolite | field | variable | Estimate | Std. Error | t value | p value | p value Bonferroni |
|---|---|---|---|---|---|---|---|---|
| All | Phosphoglycerides | 23434 | GRS (1 SD) | -0.0004859 | 0.00110974 | -0.44 | 6.62E-01 | 1.00E+00 |
| All | Phosphoglycerides | 23434 | Adjusted GRS (1 SD) | 0.00173269 | 0.00110167 | 1.57 | 1.16E-01 | 1.00E+00 |
| Statin users | TG to Phosphoglycerides ratio | 23435 | Statin dose (1 SD) | 0.00282587 | 0.00268898 | 1.05 | 2.93E-01 | 1.00E+00 |
| Statin users | TG to Phosphoglycerides ratio | 23435 | GRS (1 SD) | 0.01605038 | 0.00167586 | 9.58 | 1.11E-21 | 2.53E-19 |
| Statin users | TG to Phosphoglycerides ratio | 23435 | Adjusted GRS (1 SD) | 0.00344429 | 0.00165791 | 2.08 | 3.78E-02 | 1.00E+00 |
| Non-statin users | TG to Phosphoglycerides ratio | 23435 | GRS (1 SD) | 0.00823656 | 0.00068632 | 12.00 | 3.72E-33 | 8.49E-31 |
| Non-statin users | TG to Phosphoglycerides ratio | 23435 | Adjusted GRS (1 SD) | 0.00086403 | 0.00068202 | 1.27 | 2.05E-01 | 1.00E+00 |
| All | TG to Phosphoglycerides ratio | 23435 | Statin dose (1 SD) | 0.00049936 | 0.00290678 | 0.17 | 8.64E-01 | 1.00E+00 |
| All | TG to Phosphoglycerides ratio | 23435 | GRS (1 SD) | 0.00917421 | 0.00062234 | 14.74 | 3.87E-49 | 8.83E-47 |
| All | TG to Phosphoglycerides ratio | 23435 | Adjusted GRS (1 SD) | 0.00113647 | 0.00061839 | 1.84 | 6.61E-02 | 1.00E+00 |
| Statin users | Total Cholines | 23436 | Statin dose (1 SD) | -0.0206457 | 0.00436304 | -4.73 | 2.27E-06 | 5.16E-04 |
| Statin users | Total Cholines | 23436 | GRS (1 SD) | -6.965E-05 | 0.00276244 | -0.03 | 9.80E-01 | 1.00E+00 |
| Statin users | Total Cholines | 23436 | Adjusted GRS (1 SD) | -0.0008878 | 0.0027267 | -0.33 | 7.45E-01 | 1.00E+00 |
| Non-statin users | Total Cholines | 23436 | GRS (1 SD) | -0.0072467 | 0.00125964 | -5.75 | 8.80E-09 | 2.01E-06 |
| Non-statin users | Total Cholines | 23436 | Adjusted GRS (1 SD) | 0.0008853 | 0.00125098 | 0.71 | 4.79E-01 | 1.00E+00 |
| All | Total Cholines | 23436 | Statin dose (1 SD) | -0.0440589 | 0.00495445 | -8.89 | 7.12E-19 | 1.62E-16 |
| All | Total Cholines | 23436 | GRS (1 SD) | -0.0034359 | 0.00113823 | -3.02 | 2.54E-03 | 5.79E-01 |
| All | Total Cholines | 23436 | Adjusted GRS (1 SD) | 0.00176399 | 0.00113 | 1.56 | 1.19E-01 | 1.00E+00 |
| Statin users | Phosphatidylcholines | 23437 | Statin dose (1 SD) | -0.0172757 | 0.00402602 | -4.29 | 1.80E-05 | 4.11E-03 |
| Statin users | Phosphatidylcholines | 23437 | GRS (1 SD) | 0.00163656 | 0.00254291 | 0.64 | 5.20E-01 | 1.00E+00 |
| Statin users | Phosphatidylcholines | 23437 | Adjusted GRS (1 SD) | -0.0003394 | 0.00251004 | -0.14 | 8.92E-01 | 1.00E+00 |
| Non-statin users | Phosphatidylcholines | 23437 | GRS (1 SD) | -0.0054671 | 0.00115326 | -4.74 | 2.13E-06 | 4.87E-04 |
| Non-statin users | Phosphatidylcholines | 23437 | Adjusted GRS (1 SD) | 0.00062067 | 0.00114526 | 0.54 | 5.88E-01 | 1.00E+00 |
| All | Phosphatidylcholines | 23437 | Statin dose (1 SD) | -0.0367236 | 0.00453759 | -8.09 | 6.57E-16 | 1.50E-13 |
| All | Phosphatidylcholines | 23437 | GRS (1 SD) | -0.0021399 | 0.00103595 | -2.07 | 3.89E-02 | 1.00E+00 |
| All | Phosphatidylcholines | 23437 | Adjusted GRS (1 SD) | 0.00147203 | 0.00102844 | 1.43 | 1.52E-01 | 1.00E+00 |
| Statin users | Sphingomyelins | 23438 | Statin dose (1 SD) | -0.0030453 | 0.0006996 | -4.35 | 1.36E-05 | 3.10E-03 |
| Statin users | Sphingomyelins | 23438 | GRS (1 SD) | -0.0031359 | 0.00044725 | -7.01 | 2.44E-12 | 5.56E-10 |

| collective | metabolite | field | variable | Estimate | Std. Error | t value | p value | p value Bonferroni |
|---|---|---|---|---|---|---|---|---|
| Statin users | Sphingomyelins | 23438 | Adjusted GRS (1 SD) | -0.0003973 | 0.00044202 | -0.90 | 3.69E-01 | 1.00E+00 |
| Non-statin users | Sphingomyelins | 23438 | GRS (1 SD) | -0.0038739 | 0.00021456 | -18.06 | 9.73E-73 | 2.22E-70 |
| Non-statin users | Sphingomyelins | 23438 | Adjusted GRS (1 SD) | 9.4551E-05 | 0.00021344 | 0.44 | 6.58E-01 | 1.00E+00 |
| All | Sphingomyelins | 23438 | Statin dose (1 SD) | -0.0078928 | 0.00083056 | -9.50 | 2.57E-21 | 5.85E-19 |
| All | Sphingomyelins | 23438 | GRS (1 SD) | -0.0031428 | 0.00019782 | -15.89 | 8.95E-57 | 2.04E-54 |
| All | Sphingomyelins | 23438 | Adjusted GRS (1 SD) | 0.00026251 | 0.0001966 | 1.34 | 1.82E-01 | 1.00E+00 |
| Statin users | Apolipoprotein B | 23439 | Statin dose (1 SD) | -0.0104002 | 0.00177453 | -5.86 | 4.80E-09 | 1.10E-06 |
| Statin users | Apolipoprotein B | 23439 | GRS (1 SD) | -0.0061778 | 0.00113509 | -5.44 | 5.32E-08 | 1.21E-05 |
| Statin users | Apolipoprotein B | 23439 | Adjusted GRS (1 SD) | 0.00054313 | 0.00112129 | 0.48 | 6.28E-01 | 1.00E+00 |
| Non-statin users | Apolipoprotein B | 23439 | GRS (1 SD) | -0.0124344 | 0.00062333 | -19.95 | 2.43E-88 | 5.54E-86 |
| Non-statin users | Apolipoprotein B | 23439 | Adjusted GRS (1 SD) | 0.00030135 | 0.00062034 | 0.49 | 6.27E-01 | 1.00E+00 |
| All | Apolipoprotein B | 23439 | Statin dose (1 SD) | -0.0297632 | 0.00240556 | -12.37 | 7.05E-35 | 1.61E-32 |
| All | Apolipoprotein B | 23439 | GRS (1 SD) | -0.009313 | 0.00058492 | -15.92 | 5.14E-57 | 1.17E-54 |
| All | Apolipoprotein B | 23439 | Adjusted GRS (1 SD) | 0.00109874 | 0.00058131 | 1.89 | 5.87E-02 | 1.00E+00 |
| Statin users | Apolipoprotein A1 | 23440 | Statin dose (1 SD) | -0.0062804 | 0.00262011 | -2.40 | 1.66E-02 | 1.00E+00 |
| Statin users | Apolipoprotein A1 | 23440 | GRS (1 SD) | 0.00208473 | 0.00165443 | 1.26 | 2.08E-01 | 1.00E+00 |
| Statin users | Apolipoprotein A1 | 23440 | Adjusted GRS (1 SD) | -0.001723 | 0.0016331 | -1.06 | 2.91E-01 | 1.00E+00 |
| Non-statin users | Apolipoprotein A1 | 23440 | GRS (1 SD) | 0.00280941 | 0.00075254 | 3.73 | 1.89E-04 | 4.31E-02 |
| Non-statin users | Apolipoprotein A1 | 23440 | Adjusted GRS (1 SD) | 0.00016596 | 0.0007473 | 0.22 | 8.24E-01 | 1.00E+00 |
| All | Apolipoprotein A1 | 23440 | Statin dose (1 SD) | -0.0079081 | 0.00281796 | -2.81 | 5.02E-03 | 1.00E+00 |
| All | Apolipoprotein A1 | 23440 | GRS (1 SD) | 0.00314168 | 0.00065652 | 4.79 | 1.71E-06 | 3.90E-04 |
| All | Apolipoprotein A1 | 23440 | Adjusted GRS (1 SD) | 0.00014588 | 0.00065183 | 0.22 | 8.23E-01 | 1.00E+00 |
| Statin users | Apolipoprotein B to Apolipoprotein A1 ratio | 23441 | Statin dose (1 SD) | -0.0048137 | 0.0015141 | -3.18 | 1.48E-03 | 3.38E-01 |
| Statin users | Apolipoprotein B to Apolipoprotein A1 ratio | 23441 | GRS (1 SD) | -0.0051672 | 0.00095241 | -5.43 | 5.85E-08 | 1.33E-05 |
| Statin users | Apolipoprotein B to Apolipoprotein A1 ratio | 23441 | Adjusted GRS (1 SD) | 0.0012128 | 0.00094079 | 1.29 | 1.97E-01 | 1.00E+00 |

| collective | metabolite | field | variable | Estimate | Std. Error | t value | p value | p value Bonferroni |
|---|---|---|---|---|---|---|---|---|
| Non-statin users | Apolipoprotein B to Apolipoprotein A1 ratio | 23441 | GRS (1 SD) | -0.0096564 | 0.00053425 | -18.07 | 6.85E-73 | 1.56E-70 |
| Non-statin users | Apolipoprotein B to Apolipoprotein A1 ratio | 23441 | Adjusted GRS (1 SD) | 0.00011268 | 0.00053148 | 0.21 | 8.32E-01 | 1.00E+00 |
| All | Apolipoprotein B to Apolipoprotein A1 ratio | 23441 | Statin dose (1 SD) | -0.0179595 | 0.0019663 | -9.13 | 8.07E-20 | 1.84E-17 |
| All | Apolipoprotein B to Apolipoprotein A1 ratio | 23441 | GRS (1 SD) | -0.0076167 | 0.00048392 | -15.74 | 9.26E-56 | 2.11E-53 |
| All | Apolipoprotein B to Apolipoprotein A1 ratio | 23441 | Adjusted GRS (1 SD) | 0.00071108 | 0.00048092 | 1.48 | 1.39E-01 | 1.00E+00 |
| Statin users | Total Fatty Acids | 23442 | Statin dose (1 SD) | -0.0645436 | 0.02768564 | -2.33 | 1.98E-02 | 1.00E+00 |
| Statin users | Total Fatty Acids | 23442 | GRS (1 SD) | 0.08913892 | 0.01730496 | 5.15 | 2.62E-07 | 5.96E-05 |
| Statin users | Total Fatty Acids | 23442 | Adjusted GRS (1 SD) | 0.01530539 | 0.01709247 | 0.90 | 3.71E-01 | 1.00E+00 |
| Non-statin users | Total Fatty Acids | 23442 | GRS (1 SD) | 0.00217263 | 0.00770373 | 0.28 | 7.78E-01 | 1.00E+00 |
| Non-statin users | Total Fatty Acids | 23442 | Adjusted GRS (1 SD) | 0.01014715 | 0.00764927 | 1.33 | 1.85E-01 | 1.00E+00 |
| All | Total Fatty Acids | 23442 | Statin dose (1 SD) | -0.1996026 | 0.03183034 | -6.27 | 3.76E-10 | 8.57E-08 |
| All | Total Fatty Acids | 23442 | GRS (1 SD) | 0.0271791 | 0.00698901 | 3.89 | 1.01E-04 | 2.30E-02 |
| All | Total Fatty Acids | 23442 | Adjusted GRS (1 SD) | 0.01597273 | 0.00693855 | 2.30 | 2.13E-02 | 1.00E+00 |
| Statin users | Degree of Unsaturation | 23443 | Statin dose (1 SD) | -0.0008066 | 0.00099024 | -0.81 | 4.15E-01 | 1.00E+00 |
| Statin users | Degree of Unsaturation | 23443 | GRS (1 SD) | -0.0038399 | 0.00061223 | -6.27 | 3.64E-10 | 8.30E-08 |
| Statin users | Degree of Unsaturation | 23443 | Adjusted GRS (1 SD) | -0.000671 | 0.00060491 | -1.11 | 2.67E-01 | 1.00E+00 |
| Non-statin users | Degree of Unsaturation | 23443 | GRS (1 SD) | -0.0016123 | 0.00025252 | -6.38 | 1.72E-10 | 3.93E-08 |
| Non-statin users | Degree of Unsaturation | 23443 | Adjusted GRS (1 SD) | -7.387E-05 | 0.0002508 | -0.29 | 7.68E-01 | 1.00E+00 |
| All | Degree of Unsaturation | 23443 | Statin dose (1 SD) | -0.0005391 | 0.0010604 | -0.51 | 6.11E-01 | 1.00E+00 |
| All | Degree of Unsaturation | 23443 | GRS (1 SD) | -0.0018972 | 0.00022569 | -8.41 | 4.28E-17 | 9.76E-15 |
| All | Degree of Unsaturation | 23443 | Adjusted GRS (1 SD) | -9.829E-05 | 0.00022412 | -0.44 | 6.61E-01 | 1.00E+00 |
| Statin users | Omega-3 Fatty Acids | 23444 | Statin dose (1 SD) | 0.00223303 | 0.0023695 | 0.94 | 3.46E-01 | 1.00E+00 |
| Statin users | Omega-3 Fatty Acids | 23444 | GRS (1 SD) | 0.00539341 | 0.00149168 | 3.62 | 3.00E-04 | 6.85E-02 |

| collective | metabolite | field | variable | Estimate | Std. Error | t value | p value | p value Bonferroni |
|---|---|---|---|---|---|---|---|---|
| Statin users | Omega-3 Fatty Acids | 23444 | Adjusted GRS (1 SD) | 0.00136304 | 0.00147285 | 0.93 | 3.55E-01 | 1.00E+00 |
| Non-statin users | Omega-3 Fatty Acids | 23444 | GRS (1 SD) | 0.00159333 | 0.00071606 | 2.23 | 2.61E-02 | 1.00E+00 |
| Non-statin users | Omega-3 Fatty Acids | 23444 | Adjusted GRS (1 SD) | 0.00067972 | 0.00071102 | 0.96 | 3.39E-01 | 1.00E+00 |
| All | Omega-3 Fatty Acids | 23444 | Statin dose (1 SD) | -0.0017413 | 0.002685 | -0.65 | 5.17E-01 | 1.00E+00 |
| All | Omega-3 Fatty Acids | 23444 | GRS (1 SD) | 0.00237496 | 0.0006261 | 3.79 | 1.49E-04 | 3.39E-02 |
| All | Omega-3 Fatty Acids | 23444 | Adjusted GRS (1 SD) | 0.00109464 | 0.00062159 | 1.76 | 7.82E-02 | 1.00E+00 |
| Statin users | Omega-6 Fatty Acids | 23445 | Statin dose (1 SD) | -0.0532209 | 0.00689438 | -7.72 | 1.32E-14 | 3.02E-12 |
| Statin users | Omega-6 Fatty Acids | 23445 | GRS (1 SD) | 0.00430006 | 0.00435283 | 0.99 | 3.23E-01 | 1.00E+00 |
| Statin users | Omega-6 Fatty Acids | 23445 | Adjusted GRS (1 SD) | 0.00171621 | 0.00429662 | 0.40 | 6.90E-01 | 1.00E+00 |
| Non-statin users | Omega-6 Fatty Acids | 23445 | GRS (1 SD) | -0.0148401 | 0.00216347 | -6.86 | 6.96E-12 | 1.59E-09 |
| Non-statin users | Omega-6 Fatty Acids | 23445 | Adjusted GRS (1 SD) | 0.00213141 | 0.00214876 | 0.99 | 3.21E-01 | 1.00E+00 |
| All | Omega-6 Fatty Acids | 23445 | Statin dose (1 SD) | -0.1050832 | 0.0083094 | -12.65 | 2.39E-36 | 5.45E-34 |
| All | Omega-6 Fatty Acids | 23445 | GRS (1 SD) | -0.0062649 | 0.00196878 | -3.18 | 1.46E-03 | 3.33E-01 |
| All | Omega-6 Fatty Acids | 23445 | Adjusted GRS (1 SD) | 0.00430042 | 0.00195453 | 2.20 | 2.78E-02 | 1.00E+00 |
| Statin users | PUFA | 23446 | Statin dose (1 SD) | -0.0509883 | 0.00828293 | -6.16 | 7.86E-10 | 1.79E-07 |
| Statin users | PUFA | 23446 | GRS (1 SD) | 0.00969302 | 0.00523625 | 1.85 | 6.42E-02 | 1.00E+00 |
| Statin users | PUFA | 23446 | Adjusted GRS (1 SD) | 0.00307891 | 0.00516893 | 0.60 | 5.51E-01 | 1.00E+00 |
| Non-statin users | PUFA | 23446 | GRS (1 SD) | -0.0132467 | 0.00255351 | -5.19 | 2.13E-07 | 4.87E-05 |
| Non-statin users | PUFA | 23446 | Adjusted GRS (1 SD) | 0.00281119 | 0.00253585 | 1.11 | 2.68E-01 | 1.00E+00 |
| All | PUFA | 23446 | Statin dose (1 SD) | -0.1068252 | 0.00981911 | -10.88 | 2.14E-27 | 4.89E-25 |
| All | PUFA | 23446 | GRS (1 SD) | -0.00389 | 0.00230575 | -1.69 | 9.16E-02 | 1.00E+00 |
| All | PUFA | 23446 | Adjusted GRS (1 SD) | 0.00539507 | 0.00228898 | 2.36 | 1.84E-02 | 1.00E+00 |
| Statin users | MUFA | 23447 | Statin dose (1 SD) | 0.00138592 | 0.01004958 | 0.14 | 8.90E-01 | 1.00E+00 |
| Statin users | MUFA | 23447 | GRS (1 SD) | 0.04287392 | 0.00626195 | 6.85 | 7.78E-12 | 1.77E-09 |
| Statin users | MUFA | 23447 | Adjusted GRS (1 SD) | 0.00710855 | 0.00618823 | 1.15 | 2.51E-01 | 1.00E+00 |
| Non-statin users | MUFA | 23447 | GRS (1 SD) | 0.0112973 | 0.00262878 | 4.30 | 1.73E-05 | 3.94E-03 |
| Non-statin users | MUFA | 23447 | Adjusted GRS (1 SD) | 0.00373796 | 0.00261047 | 1.43 | 1.52E-01 | 1.00E+00 |
| All | MUFA | 23447 | Statin dose (1 SD) | -0.0295756 | 0.01126478 | -2.63 | 8.67E-03 | 1.00E+00 |

| collective | metabolite | field | variable | Estimate | Std. Error | t value | p value | p value Bonferroni |
|---|---|---|---|---|---|---|---|---|
| All | MUFA | 23447 | GRS (1 SD) | 0.01784897 | 0.0023945 | 7.45 | 9.11E-14 | 2.08E-11 |
| All | MUFA | 23447 | Adjusted GRS (1 SD) | 0.00499965 | 0.00237763 | 2.10 | 3.55E-02 | 1.00E+00 |
| Statin users | Saturated Fatty Acids | 23448 | Statin dose (1 SD) | -0.0149402 | 0.01137064 | -1.31 | 1.89E-01 | 1.00E+00 |
| Statin users | Saturated Fatty Acids | 23448 | GRS (1 SD) | 0.03657207 | 0.00705101 | 5.19 | 2.16E-07 | 4.93E-05 |
| Statin users | Saturated Fatty Acids | 23448 | Adjusted GRS (1 SD) | 0.00511812 | 0.00696455 | 0.73 | 4.62E-01 | 1.00E+00 |
| Non-statin users | Saturated Fatty Acids | 23448 | GRS (1 SD) | 0.00412256 | 0.0030502 | 1.35 | 1.77E-01 | 1.00E+00 |
| Non-statin users | Saturated Fatty Acids | 23448 | Adjusted GRS (1 SD) | 0.0035988 | 0.00302868 | 1.19 | 2.35E-01 | 1.00E+00 |
| All | Saturated Fatty Acids | 23448 | Statin dose (1 SD) | -0.0632002 | 0.01295596 | -4.88 | 1.09E-06 | 2.48E-04 |
| All | Saturated Fatty Acids | 23448 | GRS (1 SD) | 0.01322061 | 0.00277876 | 4.76 | 1.96E-06 | 4.47E-04 |
| All | Saturated Fatty Acids | 23448 | Adjusted GRS (1 SD) | 0.00557886 | 0.00275881 | 2.02 | 4.32E-02 | 1.00E+00 |
| Statin users | Linoleic Acid | 23449 | Statin dose (1 SD) | -0.0674828 | 0.00671443 | -10.05 | 1.30E-23 | 2.96E-21 |
| Statin users | Linoleic Acid | 23449 | GRS (1 SD) | 0.00744456 | 0.00424436 | 1.75 | 7.94E-02 | 1.00E+00 |
| Statin users | Linoleic Acid | 23449 | Adjusted GRS (1 SD) | 0.00320358 | 0.00418973 | 0.76 | 4.45E-01 | 1.00E+00 |
| Non-statin users | Linoleic Acid | 23449 | GRS (1 SD) | -0.0140919 | 0.00216256 | -6.52 | 7.24E-11 | 1.65E-08 |
| Non-statin users | Linoleic Acid | 23449 | Adjusted GRS (1 SD) | 0.00173258 | 0.00214781 | 0.81 | 4.20E-01 | 1.00E+00 |
| All | Linoleic Acid | 23449 | Statin dose (1 SD) | -0.1259021 | 0.00832079 | -15.13 | 4.30E-51 | 9.79E-49 |
| All | Linoleic Acid | 23449 | GRS (1 SD) | -0.0042288 | 0.00198947 | -2.13 | 3.35E-02 | 1.00E+00 |
| All | Linoleic Acid | 23449 | Adjusted GRS (1 SD) | 0.00442604 | 0.00197502 | 2.24 | 2.50E-02 | 1.00E+00 |
| Statin users | Docosahexaenoic Acid | 23450 | Statin dose (1 SD) | -3.048E-05 | 0.00086761 | -0.04 | 9.72E-01 | 1.00E+00 |
| Statin users | Docosahexaenoic Acid | 23450 | GRS (1 SD) | -0.0004062 | 0.00054702 | -0.74 | 4.58E-01 | 1.00E+00 |
| Statin users | Docosahexaenoic Acid | 23450 | Adjusted GRS (1 SD) | 0.0001317 | 0.00053995 | 0.24 | 8.07E-01 | 1.00E+00 |
| Non-statin users | Docosahexaenoic Acid | 23450 | GRS (1 SD) | -0.0007922 | 0.00026817 | -2.95 | 3.14E-03 | 7.15E-01 |
| Non-statin users | Docosahexaenoic Acid | 23450 | Adjusted GRS (1 SD) | 0.0001174 | 0.00026629 | 0.44 | 6.59E-01 | 1.00E+00 |
| All | Docosahexaenoic Acid | 23450 | Statin dose (1 SD) | -0.0015385 | 0.00099424 | -1.55 | 1.22E-01 | 1.00E+00 |
| All | Docosahexaenoic Acid | 23450 | GRS (1 SD) | -0.0005738 | 0.00023375 | -2.45 | 1.41E-02 | 1.00E+00 |
| All | Docosahexaenoic Acid | 23450 | Adjusted GRS (1 SD) | 0.00027739 | 0.00023206 | 1.20 | 2.32E-01 | 1.00E+00 |
| Statin users | Omega-3 Fatty Acids to TFA [%] | 23451 | Statin dose (1 SD) | 0.05371971 | 0.01705059 | 3.15 | 1.64E-03 | 3.73E-01 |
| Statin users | Omega-3 Fatty Acids to TFA [%] | 23451 | GRS (1 SD) | 0.00894826 | 0.01072422 | 0.83 | 4.04E-01 | 1.00E+00 |

| collective | metabolite | field | variable | Estimate | Std. Error | t value | p value | p value Bonferroni |
|---|---|---|---|---|---|---|---|---|
| Statin users | Omega-3 Fatty Acids to TFA [%] | 23451 | Adjusted GRS (1 SD) | 0.00300957 | 0.01058567 | 0.28 | 7.76E-01 | 1.00E+00 |
| Non-statin users | Omega-3 Fatty Acids to TFA [%] | 23451 | GRS (1 SD) | 0.0131171 | 0.00511859 | 2.56 | 1.04E-02 | 1.00E+00 |
| Non-statin users | Omega-3 Fatty Acids to TFA [%] | 23451 | Adjusted GRS (1 SD) | 0.00122407 | 0.00508264 | 0.24 | 8.10E-01 | 1.00E+00 |
| All | Omega-3 Fatty Acids to TFA [%] | 23451 | Statin dose (1 SD) | 0.07573192 | 0.01873687 | 4.04 | 5.35E-05 | 1.22E-02 |
| All | Omega-3 Fatty Acids to TFA [%] | 23451 | GRS (1 SD) | 0.00900554 | 0.004441 | 2.03 | 4.26E-02 | 1.00E+00 |
| All | Omega-3 Fatty Acids to TFA [%] | 23451 | Adjusted GRS (1 SD) | 0.00160246 | 0.00440883 | 0.36 | 7.16E-01 | 1.00E+00 |
| Statin users | Omega-6 Fatty Acids to TFA [%] | 23452 | Statin dose (1 SD) | -0.1798302 | 0.04647356 | -3.87 | 1.10E-04 | 2.51E-02 |
| Statin users | Omega-6 Fatty Acids to TFA [%] | 23452 | GRS (1 SD) | -0.2434684 | 0.02871525 | -8.48 | 2.44E-17 | 5.56E-15 |
| Statin users | Omega-6 Fatty Acids to TFA [%] | 23452 | Adjusted GRS (1 SD) | -0.0309544 | 0.02839554 | -1.09 | 2.76E-01 | 1.00E+00 |
| Non-statin users | Omega-6 Fatty Acids to TFA [%] | 23452 | GRS (1 SD) | -0.1277065 | 0.01133931 | -11.26 | 2.11E-29 | 4.81E-27 |
| Non-statin users | Omega-6 Fatty Acids to TFA [%] | 23452 | Adjusted GRS (1 SD) | -0.0115665 | 0.01126731 | -1.03 | 3.05E-01 | 1.00E+00 |
| All | Omega-6 Fatty Acids to TFA [%] | 23452 | Statin dose (1 SD) | -0.1965334 | 0.0497761 | -3.95 | 7.93E-05 | 1.81E-02 |
| All | Omega-6 Fatty Acids to TFA [%] | 23452 | GRS (1 SD) | -0.133994 | 0.01039341 | -12.89 | 5.29E-38 | 1.21E-35 |
| All | Omega-6 Fatty Acids to TFA [%] | 23452 | Adjusted GRS (1 SD) | -0.0113195 | 0.01032523 | -1.10 | 2.73E-01 | 1.00E+00 |
| Statin users | PUFA to TFA [%] | 23453 | Statin dose (1 SD) | -0.1261102 | 0.04855468 | -2.60 | 9.42E-03 | 1.00E+00 |
| Statin users | PUFA to TFA [%] | 23453 | GRS (1 SD) | -0.2345198 | 0.02991776 | -7.84 | 4.78E-15 | 1.09E-12 |
| Statin users | PUFA to TFA [%] | 23453 | Adjusted GRS (1 SD) | -0.0279459 | 0.02957694 | -0.94 | 3.45E-01 | 1.00E+00 |
| Non-statin users | PUFA to TFA [%] | 23453 | GRS (1 SD) | -0.1145892 | 0.01178788 | -9.72 | 2.52E-22 | 5.74E-20 |
| Non-statin users | PUFA to TFA [%] | 23453 | Adjusted GRS (1 SD) | -0.0103412 | 0.0117109 | -0.88 | 3.77E-01 | 1.00E+00 |
| All | PUFA to TFA [%] | 23453 | Statin dose (1 SD) | -0.120801 | 0.05194657 | -2.33 | 2.01E-02 | 1.00E+00 |
| All | PUFA to TFA [%] | 23453 | GRS (1 SD) | -0.1249884 | 0.01076244 | -11.61 | 3.67E-31 | 8.36E-29 |
| All | PUFA to TFA [%] | 23453 | Adjusted GRS (1 SD) | -0.0097166 | 0.01069042 | -0.91 | 3.63E-01 | 1.00E+00 |
| Statin users | MUFA to TFA [%] | 23454 | Statin dose (1 SD) | 0.09185968 | 0.0339197 | 2.71 | 6.78E-03 | 1.00E+00 |
| Statin users | MUFA to TFA [%] | 23454 | GRS (1 SD) | 0.17992248 | 0.0211619 | 8.50 | 1.99E-17 | 4.55E-15 |
| Statin users | MUFA to TFA [%] | 23454 | Adjusted GRS (1 SD) | 0.02762067 | 0.02092621 | 1.32 | 1.87E-01 | 1.00E+00 |
| Non-statin users | MUFA to TFA [%] | 23454 | GRS (1 SD) | 0.08736258 | 0.00830899 | 10.51 | 7.70E-26 | 1.75E-23 |
| Non-statin users | MUFA to TFA [%] | 23454 | Adjusted GRS (1 SD) | 0.01080927 | 0.00825544 | 1.31 | 1.90E-01 | 1.00E+00 |
| All | MUFA to TFA [%] | 23454 | Statin dose (1 SD) | 0.10558467 | 0.03602336 | 2.93 | 3.39E-03 | 7.72E-01 |

| collective | metabolite | field | variable | Estimate | Std. Error | t value | p value | p value Bonferroni |
|---|---|---|---|---|---|---|---|---|
| All | MUFA to TFA [%] | 23454 | GRS (1 SD) | 0.09209121 | 0.00760793 | 12.10 | 1.05E-33 | 2.38E-31 |
| All | MUFA to TFA [%] | 23454 | Adjusted GRS (1 SD) | 0.00956644 | 0.00755737 | 1.27 | 2.06E-01 | 1.00E+00 |
| Statin users | Saturated Fatty Acids to TFA [%] | 23455 | Statin dose (1 SD) | 0.03425673 | 0.02495615 | 1.37 | 1.70E-01 | 1.00E+00 |
| Statin users | Saturated Fatty Acids to TFA [%] | 23455 | GRS (1 SD) | 0.05460078 | 0.01539463 | 3.55 | 3.91E-04 | 8.91E-02 |
| Statin users | Saturated Fatty Acids to TFA [%] | 23455 | Adjusted GRS (1 SD) | 0.000326 | 0.01520044 | 0.02 | 9.83E-01 | 1.00E+00 |
| Non-statin users | Saturated Fatty Acids to TFA [%] | 23455 | GRS (1 SD) | 0.02722703 | 0.00634205 | 4.29 | 1.76E-05 | 4.02E-03 |
| Non-statin users | Saturated Fatty Acids to TFA [%] | 23455 | Adjusted GRS (1 SD) | -0.0004668 | 0.00629794 | -0.07 | 9.41E-01 | 1.00E+00 |
| All | Saturated Fatty Acids to TFA [%] | 23455 | Statin dose (1 SD) | 0.01522555 | 0.02679248 | 0.57 | 5.70E-01 | 1.00E+00 |
| All | Saturated Fatty Acids to TFA [%] | 23455 | GRS (1 SD) | 0.03289821 | 0.00568122 | 5.79 | 7.03E-09 | 1.60E-06 |
| All | Saturated Fatty Acids to TFA [%] | 23455 | Adjusted GRS (1 SD) | 0.0001512 | 0.00564078 | 0.03 | 9.79E-01 | 1.00E+00 |
| Statin users | Linoleic Acid to TFA [%] | 23456 | Statin dose (1 SD) | -0.4146238 | 0.03830062 | -10.83 | 4.17E-27 | 9.52E-25 |
| Statin users | Linoleic Acid to TFA [%] | 23456 | GRS (1 SD) | -0.135136 | 0.02381668 | -5.67 | 1.41E-08 | 3.23E-06 |
| Statin users | Linoleic Acid to TFA [%] | 23456 | Adjusted GRS (1 SD) | -0.0035073 | 0.02352814 | -0.15 | 8.82E-01 | 1.00E+00 |
| Non-statin users | Linoleic Acid to TFA [%] | 23456 | GRS (1 SD) | -0.1241402 | 0.01063331 | -11.67 | 1.81E-31 | 4.13E-29 |
| Non-statin users | Linoleic Acid to TFA [%] | 23456 | Adjusted GRS (1 SD) | -0.0092551 | 0.01056637 | -0.88 | 3.81E-01 | 1.00E+00 |
| All | Linoleic Acid to TFA [%] | 23456 | Statin dose (1 SD) | -0.5886435 | 0.04272525 | -13.78 | 9.47E-43 | 2.16E-40 |
| All | Linoleic Acid to TFA [%] | 23456 | GRS (1 SD) | -0.0967464 | 0.00984713 | -9.82 | 8.98E-23 | 2.05E-20 |
| All | Linoleic Acid to TFA [%] | 23456 | Adjusted GRS (1 SD) | 0.00032191 | 0.00977967 | 0.03 | 9.74E-01 | 1.00E+00 |
| Statin users | Docosahexaenoic Acid to TFA [%] | 23457 | Statin dose (1 SD) | 0.02028116 | 0.00772266 | 2.63 | 8.65E-03 | 1.00E+00 |
| Statin users | Docosahexaenoic Acid to TFA [%] | 23457 | GRS (1 SD) | -0.0198473 | 0.00483157 | -4.11 | 4.01E-05 | 9.14E-03 |
| Statin users | Docosahexaenoic Acid to TFA [%] | 23457 | Adjusted GRS (1 SD) | -0.0022753 | 0.00477112 | -0.48 | 6.33E-01 | 1.00E+00 |
| Non-statin users | Docosahexaenoic Acid to TFA [%] | 23457 | GRS (1 SD) | -0.006174 | 0.00220409 | -2.80 | 5.09E-03 | 1.00E+00 |
| Non-statin users | Docosahexaenoic Acid to TFA [%] | 23457 | Adjusted GRS (1 SD) | -0.000734 | 0.00218863 | -0.34 | 7.37E-01 | 1.00E+00 |
| All | Docosahexaenoic Acid to TFA [%] | 23457 | Statin dose (1 SD) | 0.03096679 | 0.00847477 | 3.65 | 2.60E-04 | 5.92E-02 |
| All | Docosahexaenoic Acid to TFA [%] | 23457 | GRS (1 SD) | -0.0090928 | 0.00193143 | -4.71 | 2.51E-06 | 5.72E-04 |
| All | Docosahexaenoic Acid to TFA [%] | 23457 | Adjusted GRS (1 SD) | -0.0006691 | 0.00191758 | -0.35 | 7.27E-01 | 1.00E+00 |
| Statin users | PUFA to MUFA ratio | 23458 | Statin dose (1 SD) | -0.0071212 | 0.00422466 | -1.69 | 9.19E-02 | 1.00E+00 |
| Statin users | PUFA to MUFA ratio | 23458 | GRS (1 SD) | -0.0215826 | 0.00263163 | -8.20 | 2.53E-16 | 5.76E-14 |

| collective | metabolite | field | variable | Estimate | Std. Error | t value | p value | p value Bonferroni |
|---|---|---|---|---|---|---|---|---|
| Statin users | PUFA to MUFA ratio | 23458 | Adjusted GRS (1 SD) | -0.0033594 | 0.00260198 | -1.29 | 1.97E-01 | 1.00E+00 |
| Non-statin users | PUFA to MUFA ratio | 23458 | GRS (1 SD) | -0.0111374 | 0.00111067 | -10.03 | 1.19E-23 | 2.71E-21 |
| Non-statin users | PUFA to MUFA ratio | 23458 | Adjusted GRS (1 SD) | -0.0012615 | 0.00110345 | -1.14 | 2.53E-01 | 1.00E+00 |
| All | PUFA to MUFA ratio | 23458 | Statin dose (1 SD) | -0.0071465 | 0.00448993 | -1.59 | 1.11E-01 | 1.00E+00 |
| All | PUFA to MUFA ratio | 23458 | GRS (1 SD) | -0.0116954 | 0.00099368 | -11.77 | 5.82E-32 | 1.33E-29 |
| All | PUFA to MUFA ratio | 23458 | Adjusted GRS (1 SD) | -0.0011933 | 0.00098704 | -1.21 | 2.27E-01 | 1.00E+00 |
| Statin users | Omega-6 Fatty Acids to Omega-3 Fatty Acids ratio | 23459 | Statin dose (1 SD) | -0.1593449 | 0.03505706 | -4.55 | 5.57E-06 | 1.27E-03 |
| Statin users | Omega-6 Fatty Acids to Omega-3 Fatty Acids ratio | 23459 | GRS (1 SD) | -0.0626289 | 0.02214924 | -2.83 | 4.69E-03 | 1.00E+00 |
| Statin users | Omega-6 Fatty Acids to Omega-3 Fatty Acids ratio | 23459 | Adjusted GRS (1 SD) | -0.0023511 | 0.02186725 | -0.11 | 9.14E-01 | 1.00E+00 |
| Non-statin users | Omega-6 Fatty Acids to Omega-3 Fatty Acids ratio | 23459 | GRS (1 SD) | -0.0690089 | 0.01485204 | -4.65 | 3.38E-06 | 7.71E-04 |
| Non-statin users | Omega-6 Fatty Acids to Omega-3 Fatty Acids ratio | 23459 | Adjusted GRS (1 SD) | -0.0072353 | 0.01474898 | -0.49 | 6.24E-01 | 1.00E+00 |
| All | Omega-6 Fatty Acids to Omega-3 Fatty Acids ratio | 23459 | Statin dose (1 SD) | -0.2371118 | 0.04012819 | -5.91 | 3.57E-09 | 8.14E-07 |
| All | Omega-6 Fatty Acids to Omega-3 Fatty Acids ratio | 23459 | GRS (1 SD) | -0.0532631 | 0.01245361 | -4.28 | 1.90E-05 | 4.32E-03 |
| All | Omega-6 Fatty Acids to Omega-3 Fatty Acids ratio | 23459 | Adjusted GRS (1 SD) | -0.0026891 | 0.01236415 | -0.22 | 8.28E-01 | 1.00E+00 |
| Statin users | Concentration of Chylomicrons and XL VLDL | 23481 | Statin dose (1 SD) | -1.278E-08 | 1.8335E-08 | -0.70 | 4.86E-01 | 1.00E+00 |
| Statin users | Concentration of Chylomicrons and XL VLDL | 23481 | GRS (1 SD) | 1.2421E-07 | 1.1342E-08 | 10.95 | 7.93E-28 | 1.81E-25 |
| Statin users | Concentration of Chylomicrons and XL VLDL | 23481 | Adjusted GRS (1 SD) | 1.6668E-08 | 1.123E-08 | 1.48 | 1.38E-01 | 1.00E+00 |

| collective | metabolite | field | variable | Estimate | Std. Error | t value | p value | p value Bonferroni |
|---|---|---|---|---|---|---|---|---|
| Non-statin users | Concentration of Chylomicrons and XL VLDL | 23481 | GRS (1 SD) | 7.1402E-08 | 4.81E-09 | 14.84 | 8.67E-50 | 1.98E-47 |
| Non-statin users | Concentration of Chylomicrons and XL VLDL | 23481 | Adjusted GRS (1 SD) | 5.1201E-09 | 4.7821E-09 | 1.07 | 2.84E-01 | 1.00E+00 |
| All | Concentration of Chylomicrons and XL VLDL | 23481 | Statin dose (1 SD) | -5.076E-08 | 2.0482E-08 | -2.48 | 1.32E-02 | 1.00E+00 |
| All | Concentration of Chylomicrons and XL VLDL | 23481 | GRS (1 SD) | 8.0916E-08 | 4.373E-09 | 18.50 | 2.49E-76 | 5.68E-74 |
| All | Concentration of Chylomicrons and XL VLDL | 23481 | Adjusted GRS (1 SD) | 7.2557E-09 | 4.3476E-09 | 1.67 | 9.51E-02 | 1.00E+00 |
| Statin users | Total Lipids in Chylomicrons and XL VLDL | 23482 | Statin dose (1 SD) | -0.0019398 | 0.00249848 | -0.78 | 4.38E-01 | 1.00E+00 |
| Statin users | Total Lipids in Chylomicrons and XL VLDL | 23482 | GRS (1 SD) | 0.01678103 | 0.00154068 | 10.89 | 1.51E-27 | 3.45E-25 |
| Statin users | Total Lipids in Chylomicrons and XL VLDL | 23482 | Adjusted GRS (1 SD) | 0.00216041 | 0.00152536 | 1.42 | 1.57E-01 | 1.00E+00 |
| Non-statin users | Total Lipids in Chylomicrons and XL VLDL | 23482 | GRS (1 SD) | 0.0095614 | 0.00064645 | 14.79 | 1.93E-49 | 4.40E-47 |
| Non-statin users | Total Lipids in Chylomicrons and XL VLDL | 23482 | Adjusted GRS (1 SD) | 0.00070027 | 0.00064269 | 1.09 | 2.76E-01 | 1.00E+00 |
| All | Total Lipids in Chylomicrons and XL VLDL | 23482 | Statin dose (1 SD) | -0.0070668 | 0.00278439 | -2.54 | 1.12E-02 | 1.00E+00 |
| All | Total Lipids in Chylomicrons and XL VLDL | 23482 | GRS (1 SD) | 0.01087887 | 0.00058902 | 18.47 | 4.67E-76 | 1.06E-73 |
| All | Total Lipids in Chylomicrons and XL VLDL | 23482 | Adjusted GRS (1 SD) | 0.00096056 | 0.0005856 | 1.64 | 1.01E-01 | 1.00E+00 |
| Statin users | Phospholipids in Chylomicrons and XL VLDL | 23483 | Statin dose (1 SD) | -0.0003101 | 0.0003787 | -0.82 | 4.13E-01 | 1.00E+00 |
| Statin users | Phospholipids in Chylomicrons and XL VLDL | 23483 | GRS (1 SD) | 0.00273852 | 0.00023486 | 11.66 | 2.58E-31 | 5.89E-29 |
| Statin users | Phospholipids in Chylomicrons and XL VLDL | 23483 | Adjusted GRS (1 SD) | 0.00034757 | 0.00023262 | 1.49 | 1.35E-01 | 1.00E+00 |
| Non-statin users | Phospholipids in Chylomicrons and XL VLDL | 23483 | GRS (1 SD) | 0.00164955 | 0.00010106 | 16.32 | 8.26E-60 | 1.88E-57 |
| Non-statin users | Phospholipids in Chylomicrons and XL VLDL | 23483 | Adjusted GRS (1 SD) | 9.7791E-05 | 0.0001005 | 0.97 | 3.31E-01 | 1.00E+00 |

| collective | metabolite | field | variable | Estimate | Std. Error | t value | p value | p value Bonferroni |
|---|---|---|---|---|---|---|---|---|
| All | Phospholipids in Chylomicrons and XL VLDL | 23483 | Statin dose (1 SD) | -0.0010804 | 0.00042404 | -2.55 | 1.09E-02 | 1.00E+00 |
| All | Phospholipids in Chylomicrons and XL VLDL | 23483 | GRS (1 SD) | 0.00184657 | 9.1588E-05 | 20.16 | 3.03E-90 | 6.92E-88 |
| All | Phospholipids in Chylomicrons and XL VLDL | 23483 | Adjusted GRS (1 SD) | 0.00014445 | 9.1082E-05 | 1.59 | 1.13E-01 | 1.00E+00 |
| Statin users | Cholesterol in Chylomicrons and XL VLDL | 23484 | Statin dose (1 SD) | -0.0008574 | 0.0004797 | -1.79 | 7.39E-02 | 1.00E+00 |
| Statin users | Cholesterol in Chylomicrons and XL VLDL | 23484 | GRS (1 SD) | 0.00337338 | 0.0002986 | 11.30 | 1.68E-29 | 3.82E-27 |
| Statin users | Cholesterol in Chylomicrons and XL VLDL | 23484 | Adjusted GRS (1 SD) | 0.00044227 | 0.0002957 | 1.50 | 1.35E-01 | 1.00E+00 |
| Non-statin users | Cholesterol in Chylomicrons and XL VLDL | 23484 | GRS (1 SD) | 0.00206013 | 0.00013682 | 15.06 | 3.57E-51 | 8.14E-49 |
| Non-statin users | Cholesterol in Chylomicrons and XL VLDL | 23484 | Adjusted GRS (1 SD) | 0.00015011 | 0.00013603 | 1.10 | 2.70E-01 | 1.00E+00 |
| All | Cholesterol in Chylomicrons and XL VLDL | 23484 | Statin dose (1 SD) | -0.002304 | 0.00054584 | -4.22 | 2.46E-05 | 5.60E-03 |
| All | Cholesterol in Chylomicrons and XL VLDL | 23484 | GRS (1 SD) | 0.0023486 | 0.00012263 | 19.15 | 1.24E-81 | 2.82E-79 |
| All | Cholesterol in Chylomicrons and XL VLDL | 23484 | Adjusted GRS (1 SD) | 0.00021732 | 0.00012193 | 1.78 | 7.47E-02 | 1.00E+00 |
| Statin users | CE in Chylomicrons and XL VLDL | 23485 | Statin dose (1 SD) | -0.0005641 | 0.00025524 | -2.21 | 2.71E-02 | 1.00E+00 |
| Statin users | CE in Chylomicrons and XL VLDL | 23485 | GRS (1 SD) | 0.00173692 | 0.00015923 | 10.91 | 1.27E-27 | 2.89E-25 |
| Statin users | CE in Chylomicrons and XL VLDL | 23485 | Adjusted GRS (1 SD) | 0.00023909 | 0.00015765 | 1.52 | 1.29E-01 | 1.00E+00 |
| Non-statin users | CE in Chylomicrons and XL VLDL | 23485 | GRS (1 SD) | 0.00105429 | 7.5519E-05 | 13.96 | 3.02E-44 | 6.89E-42 |
| Non-statin users | CE in Chylomicrons and XL VLDL | 23485 | Adjusted GRS (1 SD) | 8.9829E-05 | 7.507E-05 | 1.20 | 2.31E-01 | 1.00E+00 |
| All | CE in Chylomicrons and XL VLDL | 23485 | Statin dose (1 SD) | -0.0014737 | 0.00029363 | -5.02 | 5.29E-07 | 1.21E-04 |
| All | CE in Chylomicrons and XL VLDL | 23485 | GRS (1 SD) | 0.00121964 | 6.7386E-05 | 18.10 | 4.06E-73 | 9.25E-71 |
| All | CE in Chylomicrons and XL VLDL | 23485 | Adjusted GRS (1 SD) | 0.0001273 | 6.699E-05 | 1.90 | 5.74E-02 | 1.00E+00 |
| Statin users | FC in Chylomicrons and XL VLDL | 23486 | Statin dose (1 SD) | -0.0002933 | 0.00022684 | -1.29 | 1.96E-01 | 1.00E+00 |
| Statin users | FC in Chylomicrons and XL VLDL | 23486 | GRS (1 SD) | 0.00163647 | 0.00014091 | 11.61 | 4.46E-31 | 1.02E-28 |
| Statin users | FC in Chylomicrons and XL VLDL | 23486 | Adjusted GRS (1 SD) | 0.00020319 | 0.00013957 | 1.46 | 1.45E-01 | 1.00E+00 |
| Non-statin users | FC in Chylomicrons and XL VLDL | 23486 | GRS (1 SD) | 0.00100584 | 6.1997E-05 | 16.22 | 4.16E-59 | 9.48E-57 |
| Non-statin users | FC in Chylomicrons and XL VLDL | 23486 | Adjusted GRS (1 SD) | 6.0286E-05 | 6.1652E-05 | 0.98 | 3.28E-01 | 1.00E+00 |
| All | FC in Chylomicrons and XL VLDL | 23486 | Statin dose (1 SD) | -0.0008303 | 0.00025517 | -3.25 | 1.14E-03 | 2.60E-01 |

| collective | metabolite | field | variable | Estimate | Std. Error | t value | p value | p value Bonferroni |
|---|---|---|---|---|---|---|---|---|
| All | FC in Chylomicrons and XL VLDL | 23486 | GRS (1 SD) | 0.00112896 | 5.5912E-05 | 20.19 | 1.66E-90 | 3.77E-88 |
| All | FC in Chylomicrons and XL VLDL | 23486 | Adjusted GRS (1 SD) | 9.0024E-05 | 5.5603E-05 | 1.62 | 1.05E-01 | 1.00E+00 |
| Statin users | TG in Chylomicrons and XL VLDL | 23487 | Statin dose (1 SD) | -0.0007723 | 0.00166025 | -0.47 | 6.42E-01 | 1.00E+00 |
| Statin users | TG in Chylomicrons and XL VLDL | 23487 | GRS (1 SD) | 0.01066915 | 0.00101989 | 10.46 | 1.52E-25 | 3.48E-23 |
| Statin users | TG in Chylomicrons and XL VLDL | 23487 | Adjusted GRS (1 SD) | 0.00137059 | 0.00100952 | 1.36 | 1.75E-01 | 1.00E+00 |
| Non-statin users | TG in Chylomicrons and XL VLDL | 23487 | GRS (1 SD) | 0.00585173 | 0.00041428 | 14.12 | 2.99E-45 | 6.81E-43 |
| Non-statin users | TG in Chylomicrons and XL VLDL | 23487 | Adjusted GRS (1 SD) | 0.00045238 | 0.00041183 | 1.10 | 2.72E-01 | 1.00E+00 |
| All | TG in Chylomicrons and XL VLDL | 23487 | Statin dose (1 SD) | -0.0036823 | 0.00183794 | -2.00 | 4.52E-02 | 1.00E+00 |
| All | TG in Chylomicrons and XL VLDL | 23487 | GRS (1 SD) | 0.00668372 | 0.00038005 | 17.59 | 3.85E-69 | 8.78E-67 |
| All | TG in Chylomicrons and XL VLDL | 23487 | Adjusted GRS (1 SD) | 0.00059881 | 0.00037779 | 1.59 | 1.13E-01 | 1.00E+00 |
| Statin users | Concentration of Very Large VLDL | 23488 | Statin dose (1 SD) | -2.089E-08 | 2.6085E-08 | -0.80 | 4.23E-01 | 1.00E+00 |
| Statin users | Concentration of Very Large VLDL | 23488 | GRS (1 SD) | 1.3474E-07 | 1.6232E-08 | 8.30 | 1.10E-16 | 2.50E-14 |
| Statin users | Concentration of Very Large VLDL | 23488 | Adjusted GRS (1 SD) | 2.5855E-08 | 1.605E-08 | 1.61 | 1.07E-01 | 1.00E+00 |
| Non-statin users | Concentration of Very Large VLDL | 23488 | GRS (1 SD) | 5.5568E-08 | 7.2069E-09 | 7.71 | 1.27E-14 | 2.89E-12 |
| Non-statin users | Concentration of Very Large VLDL | 23488 | Adjusted GRS (1 SD) | 8.8899E-09 | 7.1586E-09 | 1.24 | 2.14E-01 | 1.00E+00 |
| All | Concentration of Very Large VLDL | 23488 | Statin dose (1 SD) | -1.021E-07 | 2.9438E-08 | -3.47 | 5.25E-04 | 1.20E-01 |
| All | Concentration of Very Large VLDL | 23488 | GRS (1 SD) | 7.1616E-08 | 6.4892E-09 | 11.04 | 2.64E-28 | 6.02E-26 |
| All | Concentration of Very Large VLDL | 23488 | Adjusted GRS (1 SD) | 1.281E-08 | 6.4455E-09 | 1.99 | 4.69E-02 | 1.00E+00 |
| Statin users | Total Lipids in Very Large VLDL | 23489 | Statin dose (1 SD) | -0.0015572 | 0.00155758 | -1.00 | 3.17E-01 | 1.00E+00 |
| Statin users | Total Lipids in Very Large VLDL | 23489 | GRS (1 SD) | 0.00809137 | 0.00096802 | 8.36 | 6.77E-17 | 1.54E-14 |
| Statin users | Total Lipids in Very Large VLDL | 23489 | Adjusted GRS (1 SD) | 0.00155585 | 0.00095719 | 1.63 | 1.04E-01 | 1.00E+00 |
| Non-statin users | Total Lipids in Very Large VLDL | 23489 | GRS (1 SD) | 0.00339073 | 0.00042452 | 7.99 | 1.40E-15 | 3.18E-13 |
| Non-statin users | Total Lipids in Very Large VLDL | 23489 | Adjusted GRS (1 SD) | 0.00053907 | 0.00042168 | 1.28 | 2.01E-01 | 1.00E+00 |
| All | Total Lipids in Very Large VLDL | 23489 | Statin dose (1 SD) | -0.0063245 | 0.0017521 | -3.61 | 3.08E-04 | 7.03E-02 |
| All | Total Lipids in Very Large VLDL | 23489 | GRS (1 SD) | 0.00435143 | 0.00038311 | 11.36 | 7.01E-30 | 1.60E-27 |
| All | Total Lipids in Very Large VLDL | 23489 | Adjusted GRS (1 SD) | 0.00077056 | 0.00038054 | 2.02 | 4.29E-02 | 1.00E+00 |
| Statin users | Phospholipids in Very Large VLDL | 23490 | Statin dose (1 SD) | -0.0003941 | 0.00029429 | -1.34 | 1.81E-01 | 1.00E+00 |
| Statin users | Phospholipids in Very Large VLDL | 23490 | GRS (1 SD) | 0.00153881 | 0.00018329 | 8.40 | 4.96E-17 | 1.13E-14 |

| collective | metabolite | field | variable | Estimate | Std. Error | t value | p value | p value Bonferroni |
|---|---|---|---|---|---|---|---|---|
| Statin users | Phospholipids in Very Large VLDL | 23490 | Adjusted GRS (1 SD) | 0.00029348 | 0.00018124 | 1.62 | 1.05E-01 | 1.00E+00 |
| Non-statin users | Phospholipids in Very Large VLDL | 23490 | GRS (1 SD) | 0.00062314 | 8.2885E-05 | 7.52 | 5.61E-14 | 1.28E-11 |
| Non-statin users | Phospholipids in Very Large VLDL | 23490 | Adjusted GRS (1 SD) | 0.00010146 | 8.2327E-05 | 1.23 | 2.18E-01 | 1.00E+00 |
| All | Phospholipids in Very Large VLDL | 23490 | Statin dose (1 SD) | -0.0014795 | 0.00033479 | -4.42 | 1.00E-05 | 2.28E-03 |
| All | Phospholipids in Very Large VLDL | 23490 | GRS (1 SD) | 0.00083061 | 7.4501E-05 | 11.15 | 7.49E-29 | 1.71E-26 |
| All | Phospholipids in Very Large VLDL | 23490 | Adjusted GRS (1 SD) | 0.00015275 | 7.3999E-05 | 2.06 | 3.90E-02 | 1.00E+00 |
| Statin users | Cholesterol in Very Large VLDL | 23491 | Statin dose (1 SD) | -0.0008318 | 0.0002967 | -2.80 | 5.07E-03 | 1.00E+00 |
| Statin users | Cholesterol in Very Large VLDL | 23491 | GRS (1 SD) | 0.00123279 | 0.00018624 | 6.62 | 3.70E-11 | 8.44E-09 |
| Statin users | Cholesterol in Very Large VLDL | 23491 | Adjusted GRS (1 SD) | 0.00029251 | 0.00018403 | 1.59 | 1.12E-01 | 1.00E+00 |
| Non-statin users | Cholesterol in Very Large VLDL | 23491 | GRS (1 SD) | 0.00028126 | 9.2122E-05 | 3.05 | 2.27E-03 | 5.16E-01 |
| Non-statin users | Cholesterol in Very Large VLDL | 23491 | Adjusted GRS (1 SD) | 0.00010955 | 9.1478E-05 | 1.20 | 2.31E-01 | 1.00E+00 |
| All | Cholesterol in Very Large VLDL | 23491 | Statin dose (1 SD) | -0.00248 | 0.00035029 | -7.08 | 1.55E-12 | 3.54E-10 |
| All | Cholesterol in Very Large VLDL | 23491 | GRS (1 SD) | 0.00056225 | 8.2239E-05 | 6.84 | 8.14E-12 | 1.86E-09 |
| All | Cholesterol in Very Large VLDL | 23491 | Adjusted GRS (1 SD) | 0.00018183 | 8.1657E-05 | 2.23 | 2.60E-02 | 1.00E+00 |
| Statin users | CE in Very Large VLDL | 23492 | Statin dose (1 SD) | -0.0005484 | 0.00014257 | -3.85 | 1.21E-04 | 2.76E-02 |
| Statin users | CE in Very Large VLDL | 23492 | GRS (1 SD) | 0.00042561 | 9.0015E-05 | 4.73 | 2.28E-06 | 5.20E-04 |
| Statin users | CE in Very Large VLDL | 23492 | Adjusted GRS (1 SD) | 0.00013335 | 8.8899E-05 | 1.50 | 1.34E-01 | 1.00E+00 |
| Non-statin users | CE in Very Large VLDL | 23492 | GRS (1 SD) | -2.462E-05 | 4.7473E-05 | -0.52 | 6.04E-01 | 1.00E+00 |
| Non-statin users | CE in Very Large VLDL | 23492 | Adjusted GRS (1 SD) | 5.4404E-05 | 4.7139E-05 | 1.15 | 2.48E-01 | 1.00E+00 |
| All | CE in Very Large VLDL | 23492 | Statin dose (1 SD) | -0.0015244 | 0.00017386 | -8.77 | 2.15E-18 | 4.91E-16 |
| All | CE in Very Large VLDL | 23492 | GRS (1 SD) | 0.00013282 | 4.2335E-05 | 3.14 | 1.71E-03 | 3.89E-01 |
| All | CE in Very Large VLDL | 23492 | Adjusted GRS (1 SD) | 9.528E-05 | 4.2029E-05 | 2.27 | 2.34E-02 | 1.00E+00 |
| Statin users | FC in Very Large VLDL | 23493 | Statin dose (1 SD) | -0.0002835 | 0.00015913 | -1.78 | 7.49E-02 | 1.00E+00 |
| Statin users | FC in Very Large VLDL | 23493 | GRS (1 SD) | 0.00080718 | 9.9364E-05 | 8.12 | 4.80E-16 | 1.09E-13 |
| Statin users | FC in Very Large VLDL | 23493 | Adjusted GRS (1 SD) | 0.00015915 | 9.8242E-05 | 1.62 | 1.05E-01 | 1.00E+00 |
| Non-statin users | FC in Very Large VLDL | 23493 | GRS (1 SD) | 0.00030588 | 4.5945E-05 | 6.66 | 2.80E-11 | 6.39E-09 |
| Non-statin users | FC in Very Large VLDL | 23493 | Adjusted GRS (1 SD) | 5.514E-05 | 4.5633E-05 | 1.21 | 2.27E-01 | 1.00E+00 |
| All | FC in Very Large VLDL | 23493 | Statin dose (1 SD) | -0.0009556 | 0.00018273 | -5.23 | 1.74E-07 | 3.96E-05 |

| collective | metabolite | field | variable | Estimate | Std. Error | t value | p value | p value Bonferroni |
|---|---|---|---|---|---|---|---|---|
| All | FC in Very Large VLDL | 23493 | GRS (1 SD) | 0.00042943 | 4.1208E-05 | 10.42 | 2.04E-25 | 4.64E-23 |
| All | FC in Very Large VLDL | 23493 | Adjusted GRS (1 SD) | 8.6549E-05 | 4.0927E-05 | 2.11 | 3.45E-02 | 1.00E+00 |
| Statin users | TG in Very Large VLDL | 23494 | Statin dose (1 SD) | -0.0003313 | 0.00098294 | -0.34 | 7.36E-01 | 1.00E+00 |
| Statin users | TG in Very Large VLDL | 23494 | GRS (1 SD) | 0.00531976 | 0.000609 | 8.74 | 2.63E-18 | 5.99E-16 |
| Statin users | TG in Very Large VLDL | 23494 | Adjusted GRS (1 SD) | 0.00096986 | 0.00060229 | 1.61 | 1.07E-01 | 1.00E+00 |
| Non-statin users | TG in Very Large VLDL | 23494 | GRS (1 SD) | 0.00248634 | 0.00025589 | 9.72 | 2.64E-22 | 6.01E-20 |
| Non-statin users | TG in Very Large VLDL | 23494 | Adjusted GRS (1 SD) | 0.00032806 | 0.00025423 | 1.29 | 1.97E-01 | 1.00E+00 |
| All | TG in Very Large VLDL | 23494 | Statin dose (1 SD) | -0.002365 | 0.00109191 | -2.17 | 3.03E-02 | 1.00E+00 |
| All | TG in Very Large VLDL | 23494 | GRS (1 SD) | 0.00295856 | 0.00023273 | 12.71 | 5.32E-37 | 1.21E-34 |
| All | TG in Very Large VLDL | 23494 | Adjusted GRS (1 SD) | 0.00043598 | 0.0002312 | 1.89 | 5.93E-02 | 1.00E+00 |
| Statin users | Concentration of Large VLDL | 23495 | Statin dose (1 SD) | -8.533E-08 | 6.1671E-08 | -1.38 | 1.67E-01 | 1.00E+00 |
| Statin users | Concentration of Large VLDL | 23495 | GRS (1 SD) | 2.7641E-07 | 3.852E-08 | 7.18 | 7.45E-13 | 1.70E-10 |
| Statin users | Concentration of Large VLDL | 23495 | Adjusted GRS (1 SD) | 6.333E-08 | 3.8071E-08 | 1.66 | 9.62E-02 | 1.00E+00 |
| Non-statin users | Concentration of Large VLDL | 23495 | GRS (1 SD) | 9.0567E-08 | 1.7583E-08 | 5.15 | 2.60E-07 | 5.92E-05 |
| Non-statin users | Concentration of Large VLDL | 23495 | Adjusted GRS (1 SD) | 2.262E-08 | 1.7462E-08 | 1.30 | 1.95E-01 | 1.00E+00 |
| All | Concentration of Large VLDL | 23495 | Statin dose (1 SD) | -3.174E-07 | 7.0283E-08 | -4.52 | 6.39E-06 | 1.46E-03 |
| All | Concentration of Large VLDL | 23495 | GRS (1 SD) | 1.3247E-07 | 1.5766E-08 | 8.40 | 4.43E-17 | 1.01E-14 |
| All | Concentration of Large VLDL | 23495 | Adjusted GRS (1 SD) | 3.3496E-08 | 1.5656E-08 | 2.14 | 3.24E-02 | 1.00E+00 |
| Statin users | Total Lipids in Large VLDL | 23496 | Statin dose (1 SD) | -0.0032078 | 0.00198434 | -1.62 | 1.06E-01 | 1.00E+00 |
| Statin users | Total Lipids in Large VLDL | 23496 | GRS (1 SD) | 0.00850255 | 0.00123784 | 6.87 | 6.67E-12 | 1.52E-09 |
| Statin users | Total Lipids in Large VLDL | 23496 | Adjusted GRS (1 SD) | 0.00205726 | 0.00122326 | 1.68 | 9.26E-02 | 1.00E+00 |
| Non-statin users | Total Lipids in Large VLDL | 23496 | GRS (1 SD) | 0.00243518 | 0.00055848 | 4.36 | 1.30E-05 | 2.96E-03 |
| Non-statin users | Total Lipids in Large VLDL | 23496 | Adjusted GRS (1 SD) | 0.00076583 | 0.0005546 | 1.38 | 1.67E-01 | 1.00E+00 |
| All | Total Lipids in Large VLDL | 23496 | Statin dose (1 SD) | -0.0106061 | 0.00224988 | -4.71 | 2.46E-06 | 5.62E-04 |
| All | Total Lipids in Large VLDL | 23496 | GRS (1 SD) | 0.00378564 | 0.00050128 | 7.55 | 4.32E-14 | 9.85E-12 |
| All | Total Lipids in Large VLDL | 23496 | Adjusted GRS (1 SD) | 0.00110466 | 0.00049776 | 2.22 | 2.65E-02 | 1.00E+00 |
| Statin users | Phospholipids in Large VLDL | 23497 | Statin dose (1 SD) | -0.0004705 | 0.00043398 | -1.08 | 2.78E-01 | 1.00E+00 |
| Statin users | Phospholipids in Large VLDL | 23497 | GRS (1 SD) | 0.00199026 | 0.00027145 | 7.33 | 2.36E-13 | 5.38E-11 |

| collective | metabolite | field | variable | Estimate | Std. Error | t value | p value | p value Bonferroni |
|---|---|---|---|---|---|---|---|---|
| Statin users | Phospholipids in Large VLDL | 23497 | Adjusted GRS (1 SD) | 0.00045869 | 0.0002683 | 1.71 | 8.74E-02 | 1.00E+00 |
| Non-statin users | Phospholipids in Large VLDL | 23497 | GRS (1 SD) | 0.00068471 | 0.00012537 | 5.46 | 4.74E-08 | 1.08E-05 |
| Non-statin users | Phospholipids in Large VLDL | 23497 | Adjusted GRS (1 SD) | 0.00016048 | 0.00012451 | 1.29 | 1.97E-01 | 1.00E+00 |
| All | Phospholipids in Large VLDL | 23497 | Statin dose (1 SD) | -0.0020916 | 0.00049469 | -4.23 | 2.38E-05 | 5.43E-03 |
| All | Phospholipids in Large VLDL | 23497 | GRS (1 SD) | 0.00097658 | 0.00011205 | 8.72 | 2.94E-18 | 6.70E-16 |
| All | Phospholipids in Large VLDL | 23497 | Adjusted GRS (1 SD) | 0.00023806 | 0.00011128 | 2.14 | 3.24E-02 | 1.00E+00 |
| Statin users | Cholesterol in Large VLDL | 23498 | Statin dose (1 SD) | -0.0012088 | 0.00048792 | -2.48 | 1.33E-02 | 1.00E+00 |
| Statin users | Cholesterol in Large VLDL | 23498 | GRS (1 SD) | 0.00173702 | 0.00030694 | 5.66 | 1.54E-08 | 3.52E-06 |
| Statin users | Cholesterol in Large VLDL | 23498 | Adjusted GRS (1 SD) | 0.0004888 | 0.00030321 | 1.61 | 1.07E-01 | 1.00E+00 |
| Non-statin users | Cholesterol in Large VLDL | 23498 | GRS (1 SD) | 0.00022729 | 0.00015325 | 1.48 | 1.38E-01 | 1.00E+00 |
| Non-statin users | Cholesterol in Large VLDL | 23498 | Adjusted GRS (1 SD) | 0.00019211 | 0.00015217 | 1.26 | 2.07E-01 | 1.00E+00 |
| All | Cholesterol in Large VLDL | 23498 | Statin dose (1 SD) | -0.0039209 | 0.00057617 | -6.81 | 1.07E-11 | 2.45E-09 |
| All | Cholesterol in Large VLDL | 23498 | GRS (1 SD) | 0.00067481 | 0.00013635 | 4.95 | 7.46E-07 | 1.70E-04 |
| All | Cholesterol in Large VLDL | 23498 | Adjusted GRS (1 SD) | 0.00031263 | 0.00013537 | 2.31 | 2.09E-02 | 1.00E+00 |
| Statin users | CE in Large VLDL | 23499 | Statin dose (1 SD) | -0.0007654 | 0.00022711 | -3.37 | 7.55E-04 | 1.72E-01 |
| Statin users | CE in Large VLDL | 23499 | GRS (1 SD) | 0.00059988 | 0.00014356 | 4.18 | 2.95E-05 | 6.72E-03 |
| Statin users | CE in Large VLDL | 23499 | Adjusted GRS (1 SD) | 0.00021991 | 0.00014176 | 1.55 | 1.21E-01 | 1.00E+00 |
| Non-statin users | CE in Large VLDL | 23499 | GRS (1 SD) | -7.588E-05 | 7.5725E-05 | -1.00 | 3.16E-01 | 1.00E+00 |
| Non-statin users | CE in Large VLDL | 23499 | Adjusted GRS (1 SD) | 9.3988E-05 | 7.5192E-05 | 1.25 | 2.11E-01 | 1.00E+00 |
| All | CE in Large VLDL | 23499 | Statin dose (1 SD) | -0.0023213 | 0.00027665 | -8.39 | 5.55E-17 | 1.27E-14 |
| All | CE in Large VLDL | 23499 | GRS (1 SD) | 0.00016784 | 6.7429E-05 | 2.49 | 1.28E-02 | 1.00E+00 |
| All | CE in Large VLDL | 23499 | Adjusted GRS (1 SD) | 0.00016195 | 6.6941E-05 | 2.42 | 1.56E-02 | 1.00E+00 |
| Statin users | FC in Large VLDL | 23500 | Statin dose (1 SD) | -0.0004434 | 0.00026749 | -1.66 | 9.75E-02 | 1.00E+00 |
| Statin users | FC in Large VLDL | 23500 | GRS (1 SD) | 0.00113712 | 0.00016752 | 6.79 | 1.17E-11 | 2.67E-09 |
| Statin users | FC in Large VLDL | 23500 | Adjusted GRS (1 SD) | 0.00026888 | 0.00016554 | 1.62 | 1.04E-01 | 1.00E+00 |
| Non-statin users | FC in Large VLDL | 23500 | GRS (1 SD) | 0.00030319 | 7.9289E-05 | 3.82 | 1.31E-04 | 3.00E-02 |
| Non-statin users | FC in Large VLDL | 23500 | Adjusted GRS (1 SD) | 9.8128E-05 | 7.8737E-05 | 1.25 | 2.13E-01 | 1.00E+00 |
| All | FC in Large VLDL | 23500 | Statin dose (1 SD) | -0.0015995 | 0.00030801 | -5.19 | 2.11E-07 | 4.82E-05 |

| collective | metabolite | field | variable | Estimate | Std. Error | t value | p value | p value Bonferroni |
|---|---|---|---|---|---|---|---|---|
| All | FC in Large VLDL | 23500 | GRS (1 SD) | 0.00050698 | 7.0736E-05 | 7.17 | 7.70E-13 | 1.76E-10 |
| All | FC in Large VLDL | 23500 | Adjusted GRS (1 SD) | 0.00015069 | 7.0238E-05 | 2.15 | 3.19E-02 | 1.00E+00 |
| Statin users | TG in Large VLDL | 23501 | Statin dose (1 SD) | -0.0015285 | 0.0010887 | -1.40 | 1.60E-01 | 1.00E+00 |
| Statin users | TG in Large VLDL | 23501 | GRS (1 SD) | 0.00477528 | 0.00067619 | 7.06 | 1.70E-12 | 3.87E-10 |
| Statin users | TG in Large VLDL | 23501 | Adjusted GRS (1 SD) | 0.00110977 | 0.00066827 | 1.66 | 9.68E-02 | 1.00E+00 |
| Non-statin users | TG in Large VLDL | 23501 | GRS (1 SD) | 0.00152316 | 0.00028921 | 5.27 | 1.39E-07 | 3.18E-05 |
| Non-statin users | TG in Large VLDL | 23501 | Adjusted GRS (1 SD) | 0.00041323 | 0.00028722 | 1.44 | 1.50E-01 | 1.00E+00 |
| All | TG in Large VLDL | 23501 | Statin dose (1 SD) | -0.0045936 | 0.00121479 | -3.78 | 1.57E-04 | 3.58E-02 |
| All | TG in Large VLDL | 23501 | GRS (1 SD) | 0.00213422 | 0.00026165 | 8.16 | 3.48E-16 | 7.93E-14 |
| All | TG in Large VLDL | 23501 | Adjusted GRS (1 SD) | 0.00055395 | 0.00025983 | 2.13 | 3.30E-02 | 1.00E+00 |
| Statin users | Concentration of Medium VLDL | 23502 | Statin dose (1 SD) | -5.618E-07 | 1.1646E-07 | -4.82 | 1.44E-06 | 3.28E-04 |
| Statin users | Concentration of Medium VLDL | 23502 | GRS (1 SD) | 5.0907E-08 | 7.3703E-08 | 0.69 | 4.90E-01 | 1.00E+00 |
| Statin users | Concentration of Medium VLDL | 23502 | Adjusted GRS (1 SD) | 8.2723E-08 | 7.275E-08 | 1.14 | 2.56E-01 | 1.00E+00 |
| Non-statin users | Concentration of Medium VLDL | 23502 | GRS (1 SD) | -3.541E-07 | 3.9311E-08 | -9.01 | 2.17E-19 | 4.95E-17 |
| Non-statin users | Concentration of Medium VLDL | 23502 | Adjusted GRS (1 SD) | 3.7792E-08 | 3.9052E-08 | 0.97 | 3.33E-01 | 1.00E+00 |
| All | Concentration of Medium VLDL | 23502 | Statin dose (1 SD) | -1.574E-06 | 1.4813E-07 | -10.62 | 3.27E-26 | 7.46E-24 |
| All | Concentration of Medium VLDL | 23502 | GRS (1 SD) | -1.882E-07 | 3.5838E-08 | -5.25 | 1.52E-07 | 3.46E-05 |
| All | Concentration of Medium VLDL | 23502 | Adjusted GRS (1 SD) | 8.1041E-08 | 3.5582E-08 | 2.28 | 2.28E-02 | 1.00E+00 |
| Statin users | Total Lipids in Medium VLDL | 23503 | Statin dose (1 SD) | -0.0083444 | 0.00207773 | -4.02 | 5.98E-05 | 1.36E-02 |
| Statin users | Total Lipids in Medium VLDL | 23503 | GRS (1 SD) | 0.00311818 | 0.00130931 | 2.38 | 1.72E-02 | 1.00E+00 |
| Statin users | Total Lipids in Medium VLDL | 23503 | Adjusted GRS (1 SD) | 0.00179537 | 0.00129254 | 1.39 | 1.65E-01 | 1.00E+00 |
| Non-statin users | Total Lipids in Medium VLDL | 23503 | GRS (1 SD) | -0.0040069 | 0.00065939 | -6.08 | 1.23E-09 | 2.81E-07 |
| Non-statin users | Total Lipids in Medium VLDL | 23503 | Adjusted GRS (1 SD) | 0.00076953 | 0.00065488 | 1.18 | 2.40E-01 | 1.00E+00 |
| All | Total Lipids in Medium VLDL | 23503 | Statin dose (1 SD) | -0.0235308 | 0.00253675 | -9.28 | 2.17E-20 | 4.95E-18 |
| All | Total Lipids in Medium VLDL | 23503 | GRS (1 SD) | -0.0014785 | 0.00059701 | -2.48 | 1.33E-02 | 1.00E+00 |
| All | Total Lipids in Medium VLDL | 23503 | Adjusted GRS (1 SD) | 0.00142897 | 0.00059268 | 2.41 | 1.59E-02 | 1.00E+00 |
| Statin users | Phospholipids in Medium VLDL | 23504 | Statin dose (1 SD) | -0.0022167 | 0.00044941 | -4.93 | 8.30E-07 | 1.89E-04 |
| Statin users | Phospholipids in Medium VLDL | 23504 | GRS (1 SD) | 2.3772E-05 | 0.00028481 | 0.08 | 9.33E-01 | 1.00E+00 |

| collective | metabolite | field | variable | Estimate | Std. Error | t value | p value | p value Bonferroni |
|---|---|---|---|---|---|---|---|---|
| Statin users | Phospholipids in Medium VLDL | 23504 | Adjusted GRS (1 SD) | 0.00032592 | 0.00028112 | 1.16 | 2.46E-01 | 1.00E+00 |
| Non-statin users | Phospholipids in Medium VLDL | 23504 | GRS (1 SD) | -0.0015307 | 0.00015189 | -10.08 | 7.16E-24 | 1.63E-21 |
| Non-statin users | Phospholipids in Medium VLDL | 23504 | Adjusted GRS (1 SD) | 0.00014606 | 0.00015091 | 0.97 | 3.33E-01 | 1.00E+00 |
| All | Phospholipids in Medium VLDL | 23504 | Statin dose (1 SD) | -0.006277 | 0.00057651 | -10.89 | 1.95E-27 | 4.46E-25 |
| All | Phospholipids in Medium VLDL | 23504 | GRS (1 SD) | -0.0008716 | 0.00013912 | -6.26 | 3.75E-10 | 8.55E-08 |
| All | Phospholipids in Medium VLDL | 23504 | Adjusted GRS (1 SD) | 0.00032052 | 0.00013814 | 2.32 | 2.03E-02 | 1.00E+00 |
| Statin users | Cholesterol in Medium VLDL | 23505 | Statin dose (1 SD) | -0.0038904 | 0.00055876 | -6.96 | 3.63E-12 | 8.28E-10 |
| Statin users | Cholesterol in Medium VLDL | 23505 | GRS (1 SD) | -0.0020076 | 0.00035673 | -5.63 | 1.85E-08 | 4.22E-06 |
| Statin users | Cholesterol in Medium VLDL | 23505 | Adjusted GRS (1 SD) | 0.00011039 | 0.00035241 | 0.31 | 7.54E-01 | 1.00E+00 |
| Non-statin users | Cholesterol in Medium VLDL | 23505 | GRS (1 SD) | -0.0037617 | 0.00020404 | -18.44 | 9.40E-76 | 2.14E-73 |
| Non-statin users | Cholesterol in Medium VLDL | 23505 | Adjusted GRS (1 SD) | 0.00010364 | 0.000203 | 0.51 | 6.10E-01 | 1.00E+00 |
| All | Cholesterol in Medium VLDL | 23505 | Statin dose (1 SD) | -0.0101829 | 0.00077141 | -13.20 | 2.03E-39 | 4.63E-37 |
| All | Cholesterol in Medium VLDL | 23505 | GRS (1 SD) | -0.0027467 | 0.00019218 | -14.29 | 2.68E-46 | 6.12E-44 |
| All | Cholesterol in Medium VLDL | 23505 | Adjusted GRS (1 SD) | 0.00036079 | 0.00019095 | 1.89 | 5.88E-02 | 1.00E+00 |
| Statin users | CE in Medium VLDL | 23506 | Statin dose (1 SD) | -0.0023912 | 0.00032272 | -7.41 | 1.41E-13 | 3.21E-11 |
| Statin users | CE in Medium VLDL | 23506 | GRS (1 SD) | -0.0016996 | 0.00020619 | -8.24 | 1.79E-16 | 4.09E-14 |
| Statin users | CE in Medium VLDL | 23506 | Adjusted GRS (1 SD) | -3.328E-05 | 0.00020389 | -0.16 | 8.70E-01 | 1.00E+00 |
| Non-statin users | CE in Medium VLDL | 23506 | GRS (1 SD) | -0.0025657 | 0.00011648 | -22.03 | 3.19E-107 | 7.27E-105 |
| Non-statin users | CE in Medium VLDL | 23506 | Adjusted GRS (1 SD) | 2.3822E-05 | 0.00011598 | 0.21 | 8.37E-01 | 1.00E+00 |
| All | CE in Medium VLDL | 23506 | Statin dose (1 SD) | -0.0060414 | 0.00044745 | -13.50 | 3.83E-41 | 8.73E-39 |
| All | CE in Medium VLDL | 23506 | GRS (1 SD) | -0.001973 | 0.00011129 | -17.73 | 3.10E-70 | 7.08E-68 |
| All | CE in Medium VLDL | 23506 | Adjusted GRS (1 SD) | 0.00017119 | 0.00011063 | 1.55 | 1.22E-01 | 1.00E+00 |
| Statin users | FC in Medium VLDL | 23507 | Statin dose (1 SD) | -0.0014992 | 0.00026525 | -5.65 | 1.64E-08 | 3.75E-06 |
| Statin users | FC in Medium VLDL | 23507 | GRS (1 SD) | -0.000308 | 0.00016844 | -1.83 | 6.75E-02 | 1.00E+00 |
| Statin users | FC in Medium VLDL | 23507 | Adjusted GRS (1 SD) | 0.00014369 | 0.00016628 | 0.86 | 3.88E-01 | 1.00E+00 |
| Non-statin users | FC in Medium VLDL | 23507 | GRS (1 SD) | -0.0011961 | 9.3517E-05 | -12.79 | 2.02E-37 | 4.60E-35 |
| Non-statin users | FC in Medium VLDL | 23507 | Adjusted GRS (1 SD) | 7.9832E-05 | 9.2945E-05 | 0.86 | 3.90E-01 | 1.00E+00 |
| All | FC in Medium VLDL | 23507 | Statin dose (1 SD) | -0.0041415 | 0.00035101 | -11.80 | 6.81E-32 | 1.55E-29 |

| collective | metabolite | field | variable | Estimate | Std. Error | t value | p value | p value Bonferroni |
|---|---|---|---|---|---|---|---|---|
| All | FC in Medium VLDL | 23507 | GRS (1 SD) | -0.0007737 | 8.6255E-05 | -8.97 | 3.01E-19 | 6.86E-17 |
| All | FC in Medium VLDL | 23507 | Adjusted GRS (1 SD) | 0.00018961 | 8.5657E-05 | 2.21 | 2.69E-02 | 1.00E+00 |
| Statin users | TG in Medium VLDL | 23508 | Statin dose (1 SD) | -0.0022373 | 0.00130212 | -1.72 | 8.58E-02 | 1.00E+00 |
| Statin users | TG in Medium VLDL | 23508 | GRS (1 SD) | 0.005102 | 0.0008151 | 6.26 | 3.95E-10 | 9.00E-08 |
| Statin users | TG in Medium VLDL | 23508 | Adjusted GRS (1 SD) | 0.00135906 | 0.00080533 | 1.69 | 9.15E-02 | 1.00E+00 |
| Non-statin users | TG in Medium VLDL | 23508 | GRS (1 SD) | 0.0012855 | 0.0003669 | 3.50 | 4.59E-04 | 1.05E-01 |
| Non-statin users | TG in Medium VLDL | 23508 | Adjusted GRS (1 SD) | 0.00051986 | 0.00036434 | 1.43 | 1.54E-01 | 1.00E+00 |
| All | TG in Medium VLDL | 23508 | Statin dose (1 SD) | -0.0070709 | 0.00147825 | -4.78 | 1.75E-06 | 3.99E-04 |
| All | TG in Medium VLDL | 23508 | GRS (1 SD) | 0.00213979 | 0.00032931 | 6.50 | 8.18E-11 | 1.87E-08 |
| All | TG in Medium VLDL | 23508 | Adjusted GRS (1 SD) | 0.00074769 | 0.00032697 | 2.29 | 2.22E-02 | 1.00E+00 |
| Statin users | Concentration of Small VLDL | 23509 | Statin dose (1 SD) | -1.368E-07 | 1.315E-07 | -1.04 | 2.98E-01 | 1.00E+00 |
| Statin users | Concentration of Small VLDL | 23509 | GRS (1 SD) | 3.0762E-07 | 8.3413E-08 | 3.69 | 2.27E-04 | 5.17E-02 |
| Statin users | Concentration of Small VLDL | 23509 | Adjusted GRS (1 SD) | 1.3119E-07 | 8.236E-08 | 1.59 | 1.11E-01 | 1.00E+00 |
| Non-statin users | Concentration of Small VLDL | 23509 | GRS (1 SD) | -6.604E-08 | 4.1626E-08 | -1.59 | 1.13E-01 | 1.00E+00 |
| Non-statin users | Concentration of Small VLDL | 23509 | Adjusted GRS (1 SD) | 4.8434E-08 | 4.1333E-08 | 1.17 | 2.41E-01 | 1.00E+00 |
| All | Concentration of Small VLDL | 23509 | Statin dose (1 SD) | -9.036E-07 | 1.5796E-07 | -5.72 | 1.10E-08 | 2.51E-06 |
| All | Concentration of Small VLDL | 23509 | GRS (1 SD) | 5.5493E-08 | 3.7178E-08 | 1.49 | 1.36E-01 | 1.00E+00 |
| All | Concentration of Small VLDL | 23509 | Adjusted GRS (1 SD) | 8.6083E-08 | 3.6908E-08 | 2.33 | 1.97E-02 | 1.00E+00 |
| Statin users | Total Lipids in Small VLDL | 23510 | Statin dose (1 SD) | -0.0021872 | 0.00131413 | -1.66 | 9.61E-02 | 1.00E+00 |
| Statin users | Total Lipids in Small VLDL | 23510 | GRS (1 SD) | 0.00278467 | 0.00083332 | 3.34 | 8.34E-04 | 1.90E-01 |
| Statin users | Total Lipids in Small VLDL | 23510 | Adjusted GRS (1 SD) | 0.00121054 | 0.00082276 | 1.47 | 1.41E-01 | 1.00E+00 |
| Non-statin users | Total Lipids in Small VLDL | 23510 | GRS (1 SD) | -0.0010262 | 0.00042312 | -2.43 | 1.53E-02 | 1.00E+00 |
| Non-statin users | Total Lipids in Small VLDL | 23510 | Adjusted GRS (1 SD) | 0.00047453 | 0.00042015 | 1.13 | 2.59E-01 | 1.00E+00 |
| All | Total Lipids in Small VLDL | 23510 | Statin dose (1 SD) | -0.0105058 | 0.00159303 | -6.59 | 4.50E-11 | 1.03E-08 |
| All | Total Lipids in Small VLDL | 23510 | GRS (1 SD) | 0.00028207 | 0.00037801 | 0.75 | 4.56E-01 | 1.00E+00 |
| All | Total Lipids in Small VLDL | 23510 | Adjusted GRS (1 SD) | 0.00086969 | 0.00037526 | 2.32 | 2.05E-02 | 1.00E+00 |
| Statin users | Phospholipids in Small VLDL | 23511 | Statin dose (1 SD) | -0.0009247 | 0.00028469 | -3.25 | 1.17E-03 | 2.66E-01 |
| Statin users | Phospholipids in Small VLDL | 23511 | GRS (1 SD) | -5.138E-05 | 0.00018113 | -0.28 | 7.77E-01 | 1.00E+00 |

| collective | metabolite | field | variable | Estimate | Std. Error | t value | p value | p value Bonferroni |
|---|---|---|---|---|---|---|---|---|
| Statin users | Phospholipids in Small VLDL | 23511 | Adjusted GRS (1 SD) | 0.00019005 | 0.00017879 | 1.06 | 2.88E-01 | 1.00E+00 |
| Non-statin users | Phospholipids in Small VLDL | 23511 | GRS (1 SD) | -0.0009361 | 9.6533E-05 | -9.70 | 3.17E-22 | 7.22E-20 |
| Non-statin users | Phospholipids in Small VLDL | 23511 | Adjusted GRS (1 SD) | 8.5086E-05 | 9.5905E-05 | 0.89 | 3.75E-01 | 1.00E+00 |
| All | Phospholipids in Small VLDL | 23511 | Statin dose (1 SD) | -0.0033171 | 0.00036206 | -9.16 | 6.23E-20 | 1.42E-17 |
| All | Phospholipids in Small VLDL | 23511 | GRS (1 SD) | -0.0005587 | 8.7505E-05 | -6.39 | 1.72E-10 | 3.91E-08 |
| All | Phospholipids in Small VLDL | 23511 | Adjusted GRS (1 SD) | 0.00019026 | 8.6884E-05 | 2.19 | 2.85E-02 | 1.00E+00 |
| Statin users | Cholesterol in Small VLDL | 23512 | Statin dose (1 SD) | -0.0016469 | 0.00046668 | -3.53 | 4.20E-04 | 9.57E-02 |
| Statin users | Cholesterol in Small VLDL | 23512 | GRS (1 SD) | -0.0005335 | 0.00029798 | -1.79 | 7.34E-02 | 1.00E+00 |
| Statin users | Cholesterol in Small VLDL | 23512 | Adjusted GRS (1 SD) | 0.00026474 | 0.00029416 | 0.90 | 3.68E-01 | 1.00E+00 |
| Non-statin users | Cholesterol in Small VLDL | 23512 | GRS (1 SD) | -0.0018129 | 0.00016574 | -10.94 | 7.86E-28 | 1.79E-25 |
| Non-statin users | Cholesterol in Small VLDL | 23512 | Adjusted GRS (1 SD) | 0.00011678 | 0.00016469 | 0.71 | 4.78E-01 | 1.00E+00 |
| All | Cholesterol in Small VLDL | 23512 | Statin dose (1 SD) | -0.0059004 | 0.00060969 | -9.68 | 4.80E-22 | 1.09E-19 |
| All | Cholesterol in Small VLDL | 23512 | GRS (1 SD) | -0.0011706 | 0.00015048 | -7.78 | 7.36E-15 | 1.68E-12 |
| All | Cholesterol in Small VLDL | 23512 | Adjusted GRS (1 SD) | 0.00030289 | 0.00014943 | 2.03 | 4.27E-02 | 1.00E+00 |
| Statin users | CE in Small VLDL | 23513 | Statin dose (1 SD) | -0.0008635 | 0.00030093 | -2.87 | 4.13E-03 | 9.41E-01 |
| Statin users | CE in Small VLDL | 23513 | GRS (1 SD) | -0.0001658 | 0.00019228 | -0.86 | 3.89E-01 | 1.00E+00 |
| Statin users | CE in Small VLDL | 23513 | Adjusted GRS (1 SD) | 0.00019115 | 0.0001898 | 1.01 | 3.14E-01 | 1.00E+00 |
| Non-statin users | CE in Small VLDL | 23513 | GRS (1 SD) | -0.0009428 | 0.00010731 | -8.78 | 1.59E-18 | 3.63E-16 |
| Non-statin users | CE in Small VLDL | 23513 | Adjusted GRS (1 SD) | 7.5967E-05 | 0.00010661 | 0.71 | 4.76E-01 | 1.00E+00 |
| All | CE in Small VLDL | 23513 | Statin dose (1 SD) | -0.003467 | 0.00038947 | -8.90 | 6.57E-19 | 1.50E-16 |
| All | CE in Small VLDL | 23513 | GRS (1 SD) | -0.0005579 | 9.6648E-05 | -5.77 | 7.83E-09 | 1.78E-06 |
| All | CE in Small VLDL | 23513 | Adjusted GRS (1 SD) | 0.00019238 | 9.596E-05 | 2.00 | 4.50E-02 | 1.00E+00 |
| Statin users | FC in Small VLDL | 23514 | Statin dose (1 SD) | -0.0007835 | 0.00016994 | -4.61 | 4.08E-06 | 9.30E-04 |
| Statin users | FC in Small VLDL | 23514 | GRS (1 SD) | -0.0003677 | 0.00010834 | -3.39 | 6.90E-04 | 1.57E-01 |
| Statin users | FC in Small VLDL | 23514 | Adjusted GRS (1 SD) | 7.3587E-05 | 0.00010698 | 0.69 | 4.92E-01 | 1.00E+00 |
| Non-statin users | FC in Small VLDL | 23514 | GRS (1 SD) | -0.0008702 | 5.969E-05 | -14.58 | 4.39E-48 | 1.00E-45 |
| Non-statin users | FC in Small VLDL | 23514 | Adjusted GRS (1 SD) | 4.0825E-05 | 5.9341E-05 | 0.69 | 4.91E-01 | 1.00E+00 |
| All | FC in Small VLDL | 23514 | Statin dose (1 SD) | -0.0024335 | 0.00022446 | -10.84 | 3.22E-27 | 7.33E-25 |

| collective | metabolite | field | variable | Estimate | Std. Error | t value | p value | p value Bonferroni |
|---|---|---|---|---|---|---|---|---|
| All | FC in Small VLDL | 23514 | GRS (1 SD) | -0.0006127 | 5.4945E-05 | -11.15 | 7.29E-29 | 1.66E-26 |
| All | FC in Small VLDL | 23514 | Adjusted GRS (1 SD) | 0.00011052 | 5.4575E-05 | 2.03 | 4.29E-02 | 1.00E+00 |
| Statin users | TG in Small VLDL | 23515 | Statin dose (1 SD) | 0.00038453 | 0.000673 | 0.57 | 5.68E-01 | 1.00E+00 |
| Statin users | TG in Small VLDL | 23515 | GRS (1 SD) | 0.00336954 | 0.00042396 | 7.95 | 2.00E-15 | 4.56E-13 |
| Statin users | TG in Small VLDL | 23515 | Adjusted GRS (1 SD) | 0.00075575 | 0.00041913 | 1.80 | 7.14E-02 | 1.00E+00 |
| Non-statin users | TG in Small VLDL | 23515 | GRS (1 SD) | 0.00172289 | 0.00019354 | 8.90 | 5.59E-19 | 1.27E-16 |
| Non-statin users | TG in Small VLDL | 23515 | Adjusted GRS (1 SD) | 0.00027264 | 0.00019226 | 1.42 | 1.56E-01 | 1.00E+00 |
| All | TG in Small VLDL | 23515 | Statin dose (1 SD) | -0.0012882 | 0.00076733 | -1.68 | 9.32E-02 | 1.00E+00 |
| All | TG in Small VLDL | 23515 | GRS (1 SD) | 0.00201143 | 0.0001731 | 11.62 | 3.38E-31 | 7.72E-29 |
| All | TG in Small VLDL | 23515 | Adjusted GRS (1 SD) | 0.00037651 | 0.00017194 | 2.19 | 2.85E-02 | 1.00E+00 |
| Statin users | Concentration of Very Small VLDL | 23516 | Statin dose (1 SD) | -4.177E-07 | 1.2548E-07 | -3.33 | 8.76E-04 | 2.00E-01 |
| Statin users | Concentration of Very Small VLDL | 23516 | GRS (1 SD) | -2.11E-07 | 8.0103E-08 | -2.63 | 8.46E-03 | 1.00E+00 |
| Statin users | Concentration of Very Small VLDL | 23516 | Adjusted GRS (1 SD) | 5.8792E-08 | 7.9082E-08 | 0.74 | 4.57E-01 | 1.00E+00 |
| Non-statin users | Concentration of Very Small VLDL | 23516 | GRS (1 SD) | -5.361E-07 | 4.327E-08 | -12.39 | 3.17E-35 | 7.23E-33 |
| Non-statin users | Concentration of Very Small VLDL | 23516 | Adjusted GRS (1 SD) | 2.8933E-08 | 4.3003E-08 | 0.67 | 5.01E-01 | 1.00E+00 |
| All | Concentration of Very Small VLDL | 23516 | Statin dose (1 SD) | -1.624E-06 | 1.6491E-07 | -9.85 | 9.03E-23 | 2.06E-20 |
| All | Concentration of Very Small VLDL | 23516 | GRS (1 SD) | -3.548E-07 | 3.982E-08 | -8.91 | 5.12E-19 | 1.17E-16 |
| All | Concentration of Very Small VLDL | 23516 | Adjusted GRS (1 SD) | 8.4475E-08 | 3.9544E-08 | 2.14 | 3.27E-02 | 1.00E+00 |
| Statin users | Total Lipids in Very Small VLDL | 23517 | Statin dose (1 SD) | -0.0025705 | 0.00082136 | -3.13 | 1.76E-03 | 4.01E-01 |
| Statin users | Total Lipids in Very Small VLDL | 23517 | GRS (1 SD) | -0.0008537 | 0.00052329 | -1.63 | 1.03E-01 | 1.00E+00 |
| Statin users | Total Lipids in Very Small VLDL | 23517 | Adjusted GRS (1 SD) | 0.0004222 | 0.00051656 | 0.82 | 4.14E-01 | 1.00E+00 |
| Non-statin users | Total Lipids in Very Small VLDL | 23517 | GRS (1 SD) | -0.0028048 | 0.00027889 | -10.06 | 8.80E-24 | 2.01E-21 |
| Non-statin users | Total Lipids in Very Small VLDL | 23517 | Adjusted GRS (1 SD) | 0.00025668 | 0.00027708 | 0.93 | 3.54E-01 | 1.00E+00 |
| All | Total Lipids in Very Small VLDL | 23517 | Statin dose (1 SD) | -0.0101554 | 0.00106556 | -9.53 | 1.97E-21 | 4.50E-19 |
| All | Total Lipids in Very Small VLDL | 23517 | GRS (1 SD) | -0.0017045 | 0.00025556 | -6.67 | 2.58E-11 | 5.88E-09 |
| All | Total Lipids in Very Small VLDL | 23517 | Adjusted GRS (1 SD) | 0.00059351 | 0.00025375 | 2.34 | 1.93E-02 | 1.00E+00 |
| Statin users | Phospholipids in Very Small VLDL | 23518 | Statin dose (1 SD) | -0.0005331 | 0.00024975 | -2.13 | 3.28E-02 | 1.00E+00 |
| Statin users | Phospholipids in Very Small VLDL | 23518 | GRS (1 SD) | -0.0001393 | 0.00015931 | -0.87 | 3.82E-01 | 1.00E+00 |

| collective | metabolite | field | variable | Estimate | Std. Error | t value | p value | p value Bonferroni |
|---|---|---|---|---|---|---|---|---|
| Statin users | Phospholipids in Very Small VLDL | 23518 | Adjusted GRS (1 SD) | 0.00014977 | 0.00015726 | 0.95 | 3.41E-01 | 1.00E+00 |
| Non-statin users | Phospholipids in Very Small VLDL | 23518 | GRS (1 SD) | -0.0006741 | 8.3687E-05 | -8.05 | 8.07E-16 | 1.84E-13 |
| Non-statin users | Phospholipids in Very Small VLDL | 23518 | Adjusted GRS (1 SD) | 8.9721E-05 | 8.3128E-05 | 1.08 | 2.80E-01 | 1.00E+00 |
| All | Phospholipids in Very Small VLDL | 23518 | Statin dose (1 SD) | -0.002699 | 0.00032137 | -8.40 | 5.21E-17 | 1.19E-14 |
| All | Phospholipids in Very Small VLDL | 23518 | GRS (1 SD) | -0.0003733 | 7.6284E-05 | -4.89 | 9.94E-07 | 2.27E-04 |
| All | Phospholipids in Very Small VLDL | 23518 | Adjusted GRS (1 SD) | 0.0001839 | 7.5737E-05 | 2.43 | 1.52E-02 | 1.00E+00 |
| Statin users | Cholesterol in Very Small VLDL | 23519 | Statin dose (1 SD) | -0.0026473 | 0.00042801 | -6.19 | 6.54E-10 | 1.49E-07 |
| Statin users | Cholesterol in Very Small VLDL | 23519 | GRS (1 SD) | -0.0016548 | 0.000273 | -6.06 | 1.37E-09 | 3.13E-07 |
| Statin users | Cholesterol in Very Small VLDL | 23519 | Adjusted GRS (1 SD) | 5.2928E-05 | 0.00026973 | 0.20 | 8.44E-01 | 1.00E+00 |
| Non-statin users | Cholesterol in Very Small VLDL | 23519 | GRS (1 SD) | -0.0026387 | 0.00014885 | -17.73 | 3.44E-70 | 7.84E-68 |
| Non-statin users | Cholesterol in Very Small VLDL | 23519 | Adjusted GRS (1 SD) | 8.0435E-05 | 0.00014807 | 0.54 | 5.87E-01 | 1.00E+00 |
| All | Cholesterol in Very Small VLDL | 23519 | Statin dose (1 SD) | -0.0073785 | 0.00057843 | -12.76 | 6.03E-37 | 1.37E-34 |
| All | Cholesterol in Very Small VLDL | 23519 | GRS (1 SD) | -0.0019284 | 0.00014109 | -13.67 | 1.68E-42 | 3.84E-40 |
| All | Cholesterol in Very Small VLDL | 23519 | Adjusted GRS (1 SD) | 0.00028327 | 0.00014017 | 2.02 | 4.33E-02 | 1.00E+00 |
| Statin users | CE in Very Small VLDL | 23520 | Statin dose (1 SD) | -0.0021252 | 0.00030268 | -7.02 | 2.40E-12 | 5.46E-10 |
| Statin users | CE in Very Small VLDL | 23520 | GRS (1 SD) | -0.0013531 | 0.00019315 | -7.01 | 2.54E-12 | 5.80E-10 |
| Statin users | CE in Very Small VLDL | 23520 | Adjusted GRS (1 SD) | 5.9126E-06 | 0.0001909 | 0.03 | 9.75E-01 | 1.00E+00 |
| Non-statin users | CE in Very Small VLDL | 23520 | GRS (1 SD) | -0.0020663 | 0.00010451 | -19.77 | 8.28E-87 | 1.89E-84 |
| Non-statin users | CE in Very Small VLDL | 23520 | Adjusted GRS (1 SD) | 3.9362E-05 | 0.00010401 | 0.38 | 7.05E-01 | 1.00E+00 |
| All | CE in Very Small VLDL | 23520 | Statin dose (1 SD) | -0.0055314 | 0.00040955 | -13.51 | 3.62E-41 | 8.25E-39 |
| All | CE in Very Small VLDL | 23520 | GRS (1 SD) | -0.0015416 | 0.00010004 | -15.41 | 1.61E-53 | 3.68E-51 |
| All | CE in Very Small VLDL | 23520 | Adjusted GRS (1 SD) | 0.00018651 | 9.9419E-05 | 1.88 | 6.07E-02 | 1.00E+00 |
| Statin users | FC in Very Small VLDL | 23521 | Statin dose (1 SD) | -0.0005221 | 0.00013294 | -3.93 | 8.68E-05 | 1.98E-02 |
| Statin users | FC in Very Small VLDL | 23521 | GRS (1 SD) | -0.0003017 | 8.4777E-05 | -3.56 | 3.74E-04 | 8.52E-02 |
| Statin users | FC in Very Small VLDL | 23521 | Adjusted GRS (1 SD) | 4.7046E-05 | 8.3709E-05 | 0.56 | 5.74E-01 | 1.00E+00 |
| Non-statin users | FC in Very Small VLDL | 23521 | GRS (1 SD) | -0.0005724 | 4.62E-05 | -12.39 | 3.18E-35 | 7.25E-33 |
| Non-statin users | FC in Very Small VLDL | 23521 | Adjusted GRS (1 SD) | 4.1088E-05 | 4.5915E-05 | 0.89 | 3.71E-01 | 1.00E+00 |
| All | FC in Very Small VLDL | 23521 | Statin dose (1 SD) | -0.001847 | 0.00017651 | -10.46 | 1.77E-25 | 4.03E-23 |

| collective | metabolite | field | variable | Estimate | Std. Error | t value | p value | p value Bonferroni |
|---|---|---|---|---|---|---|---|---|
| All | FC in Very Small VLDL | 23521 | GRS (1 SD) | -0.0003869 | 4.2739E-05 | -9.05 | 1.43E-19 | 3.25E-17 |
| All | FC in Very Small VLDL | 23521 | Adjusted GRS (1 SD) | 9.6777E-05 | 4.2443E-05 | 2.28 | 2.26E-02 | 1.00E+00 |
| Statin users | TG in Very Small VLDL | 23522 | Statin dose (1 SD) | 0.00060989 | 0.00023302 | 2.62 | 8.88E-03 | 1.00E+00 |
| Statin users | TG in Very Small VLDL | 23522 | GRS (1 SD) | 0.00094029 | 0.00014825 | 6.34 | 2.31E-10 | 5.26E-08 |
| Statin users | TG in Very Small VLDL | 23522 | Adjusted GRS (1 SD) | 0.00021942 | 0.00014648 | 1.50 | 1.34E-01 | 1.00E+00 |
| Non-statin users | TG in Very Small VLDL | 23522 | GRS (1 SD) | 0.00050795 | 6.9352E-05 | 7.32 | 2.42E-13 | 5.53E-11 |
| Non-statin users | TG in Very Small VLDL | 23522 | Adjusted GRS (1 SD) | 8.6514E-05 | 6.8885E-05 | 1.26 | 2.09E-01 | 1.00E+00 |
| All | TG in Very Small VLDL | 23522 | Statin dose (1 SD) | -7.796E-05 | 0.00027117 | -0.29 | 7.74E-01 | 1.00E+00 |
| All | TG in Very Small VLDL | 23522 | GRS (1 SD) | 0.0005972 | 6.1884E-05 | 9.65 | 5.00E-22 | 1.14E-19 |
| All | TG in Very Small VLDL | 23522 | Adjusted GRS (1 SD) | 0.00012633 | 6.1459E-05 | 2.06 | 3.98E-02 | 1.00E+00 |
| Statin users | Concentration of IDL | 23523 | Statin dose (1 SD) | -5.066E-06 | 6.4589E-07 | -7.84 | 4.98E-15 | 1.14E-12 |
| Statin users | Concentration of IDL | 23523 | GRS (1 SD) | -3.68E-06 | 4.1507E-07 | -8.87 | 8.16E-19 | 1.86E-16 |
| Statin users | Concentration of IDL | 23523 | Adjusted GRS (1 SD) | 1.862E-08 | 4.1054E-07 | 0.05 | 9.64E-01 | 1.00E+00 |
| Non-statin users | Concentration of IDL | 23523 | GRS (1 SD) | -5.951E-06 | 2.3329E-07 | -25.51 | 5.23E-143 | 1.19E-140 |
| Non-statin users | Concentration of IDL | 23523 | Adjusted GRS (1 SD) | -5.879E-09 | 2.325E-07 | -0.03 | 9.80E-01 | 1.00E+00 |
| All | Concentration of IDL | 23523 | Statin dose (1 SD) | -1.261E-05 | 8.9292E-07 | -14.12 | 8.27E-45 | 1.89E-42 |
| All | Concentration of IDL | 23523 | GRS (1 SD) | -4.682E-06 | 2.2234E-07 | -21.06 | 3.03E-98 | 6.90E-96 |
| All | Concentration of IDL | 23523 | Adjusted GRS (1 SD) | 3.3091E-07 | 2.2114E-07 | 1.50 | 1.35E-01 | 1.00E+00 |
| Statin users | Total Lipids in IDL | 23524 | Statin dose (1 SD) | -0.0189885 | 0.00252624 | -7.52 | 6.29E-14 | 1.43E-11 |
| Statin users | Total Lipids in IDL | 23524 | GRS (1 SD) | -0.0144584 | 0.00162485 | -8.90 | 6.16E-19 | 1.40E-16 |
| Statin users | Total Lipids in IDL | 23524 | Adjusted GRS (1 SD) | -0.0005319 | 0.00160715 | -0.33 | 7.41E-01 | 1.00E+00 |
| Non-statin users | Total Lipids in IDL | 23524 | GRS (1 SD) | -0.0222345 | 0.00085467 | -26.02 | 1.22E-148 | 2.78E-146 |
| Non-statin users | Total Lipids in IDL | 23524 | Adjusted GRS (1 SD) | 0.00011126 | 0.00085192 | 0.13 | 8.96E-01 | 1.00E+00 |
| All | Total Lipids in IDL | 23524 | Statin dose (1 SD) | -0.0469831 | 0.00338028 | -13.90 | 1.80E-43 | 4.11E-41 |
| All | Total Lipids in IDL | 23524 | GRS (1 SD) | -0.0176092 | 0.00082103 | -21.45 | 7.55E-102 | 1.72E-99 |
| All | Total Lipids in IDL | 23524 | Adjusted GRS (1 SD) | 0.00127097 | 0.00081667 | 1.56 | 1.20E-01 | 1.00E+00 |
| Statin users | Phospholipids in IDL | 23525 | Statin dose (1 SD) | -0.0040747 | 0.00057211 | -7.12 | 1.16E-12 | 2.65E-10 |
| Statin users | Phospholipids in IDL | 23525 | GRS (1 SD) | -0.0031102 | 0.00036757 | -8.46 | 2.83E-17 | 6.44E-15 |

| collective | metabolite | field | variable | Estimate | Std. Error | t value | p value | p value Bonferroni |
|---|---|---|---|---|---|---|---|---|
| Statin users | Phospholipids in IDL | 23525 | Adjusted GRS (1 SD) | -7.344E-05 | 0.0003635 | -0.20 | 8.40E-01 | 1.00E+00 |
| Non-statin users | Phospholipids in IDL | 23525 | GRS (1 SD) | -0.0046678 | 0.00019533 | -23.90 | 8.46E-126 | 1.93E-123 |
| Non-statin users | Phospholipids in IDL | 23525 | Adjusted GRS (1 SD) | 4.9024E-05 | 0.00019459 | 0.25 | 8.01E-01 | 1.00E+00 |
| All | Phospholipids in IDL | 23525 | Statin dose (1 SD) | -0.0105168 | 0.00077466 | -13.58 | 1.43E-41 | 3.25E-39 |
| All | Phospholipids in IDL | 23525 | GRS (1 SD) | -0.0036552 | 0.00018768 | -19.48 | 2.40E-84 | 5.48E-82 |
| All | Phospholipids in IDL | 23525 | Adjusted GRS (1 SD) | 0.00031211 | 0.00018662 | 1.67 | 9.44E-02 | 1.00E+00 |
| Statin users | Cholesterol in IDL | 23526 | Statin dose (1 SD) | -0.0155034 | 0.00187884 | -8.25 | 1.84E-16 | 4.19E-14 |
| Statin users | Cholesterol in IDL | 23526 | GRS (1 SD) | -0.0120243 | 0.00120894 | -9.95 | 2.98E-23 | 6.79E-21 |
| Statin users | Cholesterol in IDL | 23526 | Adjusted GRS (1 SD) | -0.0006602 | 0.00119637 | -0.55 | 5.81E-01 | 1.00E+00 |
| Non-statin users | Cholesterol in IDL | 23526 | GRS (1 SD) | -0.01775 | 0.00063062 | -28.15 | 1.54E-173 | 3.51E-171 |
| Non-statin users | Cholesterol in IDL | 23526 | Adjusted GRS (1 SD) | -3.405E-05 | 0.00062899 | -0.05 | 9.57E-01 | 1.00E+00 |
| All | Cholesterol in IDL | 23526 | Statin dose (1 SD) | -0.0359114 | 0.00249491 | -14.39 | 1.85E-46 | 4.22E-44 |
| All | Cholesterol in IDL | 23526 | GRS (1 SD) | -0.0142792 | 0.00060878 | -23.46 | 2.21E-121 | 5.04E-119 |
| All | Cholesterol in IDL | 23526 | Adjusted GRS (1 SD) | 0.0008084 | 0.00060578 | 1.33 | 1.82E-01 | 1.00E+00 |
| Statin users | CE in IDL | 23527 | Statin dose (1 SD) | -0.0117417 | 0.00139723 | -8.40 | 5.15E-17 | 1.18E-14 |
| Statin users | CE in IDL | 23527 | GRS (1 SD) | -0.0087647 | 0.00089941 | -9.74 | 2.18E-22 | 4.98E-20 |
| Statin users | CE in IDL | 23527 | Adjusted GRS (1 SD) | -0.0004017 | 0.00088998 | -0.45 | 6.52E-01 | 1.00E+00 |
| Non-statin users | CE in IDL | 23527 | GRS (1 SD) | -0.0133787 | 0.00046918 | -28.51 | 4.98E-178 | 1.13E-175 |
| Non-statin users | CE in IDL | 23527 | Adjusted GRS (1 SD) | -1.049E-05 | 0.00046803 | -0.02 | 9.82E-01 | 1.00E+00 |
| All | CE in IDL | 23527 | Statin dose (1 SD) | -0.0268676 | 0.00184989 | -14.52 | 2.92E-47 | 6.67E-45 |
| All | CE in IDL | 23527 | GRS (1 SD) | -0.0107458 | 0.00045199 | -23.77 | 1.22E-124 | 2.78E-122 |
| All | CE in IDL | 23527 | Adjusted GRS (1 SD) | 0.00062839 | 0.0004498 | 1.40 | 1.62E-01 | 1.00E+00 |
| Statin users | FC in IDL | 23528 | Statin dose (1 SD) | -0.0037617 | 0.00049659 | -7.57 | 4.03E-14 | 9.19E-12 |
| Statin users | FC in IDL | 23528 | GRS (1 SD) | -0.0032597 | 0.00031896 | -10.22 | 1.87E-24 | 4.25E-22 |
| Statin users | FC in IDL | 23528 | Adjusted GRS (1 SD) | -0.0002586 | 0.00031569 | -0.82 | 4.13E-01 | 1.00E+00 |
| Non-statin users | FC in IDL | 23528 | GRS (1 SD) | -0.0043713 | 0.00016618 | -26.30 | 6.55E-152 | 1.49E-149 |
| Non-statin users | FC in IDL | 23528 | Adjusted GRS (1 SD) | -2.359E-05 | 0.00016566 | -0.14 | 8.87E-01 | 1.00E+00 |
| All | FC in IDL | 23528 | Statin dose (1 SD) | -0.0090435 | 0.00065928 | -13.72 | 2.13E-42 | 4.86E-40 |

| collective | metabolite | field | variable | Estimate | Std. Error | t value | p value | p value Bonferroni |
|---|---|---|---|---|---|---|---|---|
| All | FC in IDL | 23528 | GRS (1 SD) | -0.0035335 | 0.00016043 | -22.03 | 2.72E-107 | 6.19E-105 |
| All | FC in IDL | 23528 | Adjusted GRS (1 SD) | 0.00017997 | 0.00015959 | 1.13 | 2.59E-01 | 1.00E+00 |
| Statin users | TG in IDL | 23529 | Statin dose (1 SD) | 0.00058957 | 0.00027437 | 2.15 | 3.17E-02 | 1.00E+00 |
| Statin users | TG in IDL | 23529 | GRS (1 SD) | 0.00067621 | 0.00017484 | 3.87 | 1.10E-04 | 2.51E-02 |
| Statin users | TG in IDL | 23529 | Adjusted GRS (1 SD) | 0.00020181 | 0.00017264 | 1.17 | 2.42E-01 | 1.00E+00 |
| Non-statin users | TG in IDL | 23529 | GRS (1 SD) | 0.00018317 | 8.5306E-05 | 2.15 | 3.18E-02 | 1.00E+00 |
| Non-statin users | TG in IDL | 23529 | Adjusted GRS (1 SD) | 9.6232E-05 | 8.4707E-05 | 1.14 | 2.56E-01 | 1.00E+00 |
| All | TG in IDL | 23529 | Statin dose (1 SD) | -0.000555 | 0.00032604 | -1.70 | 8.87E-02 | 1.00E+00 |
| All | TG in IDL | 23529 | GRS (1 SD) | 0.00032513 | 7.5771E-05 | 4.29 | 1.78E-05 | 4.06E-03 |
| All | TG in IDL | 23529 | Adjusted GRS (1 SD) | 0.00015046 | 7.5227E-05 | 2.00 | 4.55E-02 | 1.00E+00 |
| Statin users | Concentration of Large LDL | 23530 | Statin dose (1 SD) | -8.231E-06 | 1.5569E-06 | -5.29 | 1.28E-07 | 2.92E-05 |
| Statin users | Concentration of Large LDL | 23530 | GRS (1 SD) | -6.347E-06 | 9.9617E-07 | -6.37 | 1.91E-10 | 4.36E-08 |
| Statin users | Concentration of Large LDL | 23530 | Adjusted GRS (1 SD) | 3.2354E-07 | 9.8434E-07 | 0.33 | 7.42E-01 | 1.00E+00 |
| Non-statin users | Concentration of Large LDL | 23530 | GRS (1 SD) | -1.173E-05 | 5.4934E-07 | -21.36 | 6.31E-101 | 1.44E-98 |
| Non-statin users | Concentration of Large LDL | 23530 | Adjusted GRS (1 SD) | 2.9486E-07 | 5.4689E-07 | 0.54 | 5.90E-01 | 1.00E+00 |
| All | Concentration of Large LDL | 23530 | Statin dose (1 SD) | -2.505E-05 | 2.1135E-06 | -11.85 | 3.69E-32 | 8.41E-30 |
| All | Concentration of Large LDL | 23530 | GRS (1 SD) | -9.041E-06 | 5.1404E-07 | -17.59 | 3.69E-69 | 8.41E-67 |
| All | Concentration of Large LDL | 23530 | Adjusted GRS (1 SD) | 9.3301E-07 | 5.1098E-07 | 1.83 | 6.79E-02 | 1.00E+00 |
| Statin users | Total Lipids in Large LDL | 23531 | Statin dose (1 SD) | -0.0206543 | 0.00344843 | -5.99 | 2.20E-09 | 5.03E-07 |
| Statin users | Total Lipids in Large LDL | 23531 | GRS (1 SD) | -0.0173759 | 0.00221511 | -7.84 | 4.58E-15 | 1.04E-12 |
| Statin users | Total Lipids in Large LDL | 23531 | Adjusted GRS (1 SD) | -0.0004318 | 0.00218999 | -0.20 | 8.44E-01 | 1.00E+00 |
| Non-statin users | Total Lipids in Large LDL | 23531 | GRS (1 SD) | -0.0284102 | 0.00116862 | -24.31 | 4.05E-130 | 9.24E-128 |
| Non-statin users | Total Lipids in Large LDL | 23531 | Adjusted GRS (1 SD) | 4.1139E-05 | 0.00116429 | 0.04 | 9.72E-01 | 1.00E+00 |
| All | Total Lipids in Large LDL | 23531 | Statin dose (1 SD) | -0.0543698 | 0.00449418 | -12.10 | 1.97E-33 | 4.49E-31 |
| All | Total Lipids in Large LDL | 23531 | GRS (1 SD) | -0.0228006 | 0.00109273 | -20.87 | 1.65E-96 | 3.75E-94 |
| All | Total Lipids in Large LDL | 23531 | Adjusted GRS (1 SD) | 0.00140966 | 0.00108682 | 1.30 | 1.95E-01 | 1.00E+00 |
| Statin users | Phospholipids in Large LDL | 23532 | Statin dose (1 SD) | -0.005047 | 0.00075052 | -6.72 | 1.89E-11 | 4.31E-09 |
| Statin users | Phospholipids in Large LDL | 23532 | GRS (1 SD) | -0.0041961 | 0.00048176 | -8.71 | 3.28E-18 | 7.48E-16 |

| collective | metabolite | field | variable | Estimate | Std. Error | t value | p value | p value Bonferroni |
|---|---|---|---|---|---|---|---|---|
| Statin users | Phospholipids in Large LDL | 23532 | Adjusted GRS (1 SD) | -0.0002151 | 0.00047647 | -0.45 | 6.52E-01 | 1.00E+00 |
| Non-statin users | Phospholipids in Large LDL | 23532 | GRS (1 SD) | -0.006446 | 0.00025472 | -25.31 | 8.69E-141 | 1.98E-138 |
| Non-statin users | Phospholipids in Large LDL | 23532 | Adjusted GRS (1 SD) | -1.099E-07 | 0.00025385 | 0.00 | 1.00E+00 | 1.00E+00 |
| All | Phospholipids in Large LDL | 23532 | Statin dose (1 SD) | -0.0125457 | 0.00098337 | -12.76 | 5.89E-37 | 1.34E-34 |
| All | Phospholipids in Large LDL | 23532 | GRS (1 SD) | -0.005196 | 0.00023967 | -21.68 | 5.07E-104 | 1.16E-101 |
| All | Phospholipids in Large LDL | 23532 | Adjusted GRS (1 SD) | 0.00029413 | 0.00023841 | 1.23 | 2.17E-01 | 1.00E+00 |
| Statin users | Cholesterol in Large LDL | 23533 | Statin dose (1 SD) | -0.0162477 | 0.00258017 | -6.30 | 3.21E-10 | 7.31E-08 |
| Statin users | Cholesterol in Large LDL | 23533 | GRS (1 SD) | -0.0136393 | 0.00165727 | -8.23 | 1.99E-16 | 4.54E-14 |
| Statin users | Cholesterol in Large LDL | 23533 | Adjusted GRS (1 SD) | -0.0003786 | 0.00163874 | -0.23 | 8.17E-01 | 1.00E+00 |
| Non-statin users | Cholesterol in Large LDL | 23533 | GRS (1 SD) | -0.0218756 | 0.00087498 | -25.00 | 1.80E-137 | 4.11E-135 |
| Non-statin users | Cholesterol in Large LDL | 23533 | Adjusted GRS (1 SD) | -4.85E-05 | 0.00087191 | -0.06 | 9.56E-01 | 1.00E+00 |
| All | Cholesterol in Large LDL | 23533 | Statin dose (1 SD) | -0.0412962 | 0.00335926 | -12.29 | 1.86E-34 | 4.24E-32 |
| All | Cholesterol in Large LDL | 23533 | GRS (1 SD) | -0.0176677 | 0.00081909 | -21.57 | 5.48E-103 | 1.25E-100 |
| All | Cholesterol in Large LDL | 23533 | Adjusted GRS (1 SD) | 0.00097893 | 0.00081477 | 1.20 | 2.30E-01 | 1.00E+00 |
| Statin users | CE in Large LDL | 23534 | Statin dose (1 SD) | -0.011486 | 0.00191526 | -6.00 | 2.10E-09 | 4.80E-07 |
| Statin users | CE in Large LDL | 23534 | GRS (1 SD) | -0.0091081 | 0.00122882 | -7.41 | 1.29E-13 | 2.95E-11 |
| Statin users | CE in Large LDL | 23534 | Adjusted GRS (1 SD) | -9.999E-05 | 0.00121468 | -0.08 | 9.34E-01 | 1.00E+00 |
| Non-statin users | CE in Large LDL | 23534 | GRS (1 SD) | -0.015308 | 0.00064698 | -23.66 | 2.24E-123 | 5.10E-121 |
| Non-statin users | CE in Large LDL | 23534 | Adjusted GRS (1 SD) | 1.2659E-05 | 0.00064447 | 0.02 | 9.84E-01 | 1.00E+00 |
| All | CE in Large LDL | 23534 | Statin dose (1 SD) | -0.0296056 | 0.00247647 | -11.95 | 1.08E-32 | 2.47E-30 |
| All | CE in Large LDL | 23534 | GRS (1 SD) | -0.0122786 | 0.00060225 | -20.39 | 3.11E-92 | 7.08E-90 |
| All | CE in Large LDL | 23534 | Adjusted GRS (1 SD) | 0.00076526 | 0.00059895 | 1.28 | 2.01E-01 | 1.00E+00 |
| Statin users | FC in Large LDL | 23535 | Statin dose (1 SD) | -0.0047618 | 0.00069208 | -6.88 | 6.46E-12 | 1.47E-09 |
| Statin users | FC in Large LDL | 23535 | GRS (1 SD) | -0.0045312 | 0.00044523 | -10.18 | 2.89E-24 | 6.58E-22 |
| Statin users | FC in Large LDL | 23535 | Adjusted GRS (1 SD) | -0.0002787 | 0.00044066 | -0.63 | 5.27E-01 | 1.00E+00 |
| Non-statin users | FC in Large LDL | 23535 | GRS (1 SD) | -0.0065676 | 0.00023463 | -27.99 | 1.18E-171 | 2.70E-169 |
| Non-statin users | FC in Large LDL | 23535 | Adjusted GRS (1 SD) | -6.12E-05 | 0.00023402 | -0.26 | 7.94E-01 | 1.00E+00 |
| All | FC in Large LDL | 23535 | Statin dose (1 SD) | -0.0116908 | 0.00090892 | -12.86 | 1.57E-37 | 3.58E-35 |

| collective | metabolite | field | variable | Estimate | Std. Error | t value | p value | p value Bonferroni |
|---|---|---|---|---|---|---|---|---|
| All | FC in Large LDL | 23535 | GRS (1 SD) | -0.0053891 | 0.00022263 | -24.21 | 3.91E-129 | 8.91E-127 |
| All | FC in Large LDL | 23535 | Adjusted GRS (1 SD) | 0.0002136 | 0.00022156 | 0.96 | 3.35E-01 | 1.00E+00 |
| Statin users | TG in Large LDL | 23536 | Statin dose (1 SD) | 0.00064073 | 0.00026925 | 2.38 | 1.74E-02 | 1.00E+00 |
| Statin users | TG in Large LDL | 23536 | GRS (1 SD) | 0.00045967 | 0.00017203 | 2.67 | 7.54E-03 | 1.00E+00 |
| Statin users | TG in Large LDL | 23536 | Adjusted GRS (1 SD) | 0.00016225 | 0.00016983 | 0.96 | 3.39E-01 | 1.00E+00 |
| Non-statin users | TG in Large LDL | 23536 | GRS (1 SD) | -8.864E-05 | 8.3249E-05 | -1.06 | 2.87E-01 | 1.00E+00 |
| Non-statin users | TG in Large LDL | 23536 | Adjusted GRS (1 SD) | 8.989E-05 | 8.2663E-05 | 1.09 | 2.77E-01 | 1.00E+00 |
| All | TG in Large LDL | 23536 | Statin dose (1 SD) | -0.0005275 | 0.00032124 | -1.64 | 1.01E-01 | 1.00E+00 |
| All | TG in Large LDL | 23536 | GRS (1 SD) | 6.3183E-05 | 7.4307E-05 | 0.85 | 3.95E-01 | 1.00E+00 |
| All | TG in Large LDL | 23536 | Adjusted GRS (1 SD) | 0.00013673 | 7.3767E-05 | 1.85 | 6.38E-02 | 1.00E+00 |
| Statin users | Concentration of Medium LDL | 23537 | Statin dose (1 SD) | -3.93E-06 | 7.0128E-07 | -5.60 | 2.17E-08 | 4.94E-06 |
| Statin users | Concentration of Medium LDL | 23537 | GRS (1 SD) | -1.952E-06 | 4.4592E-07 | -4.38 | 1.21E-05 | 2.76E-03 |
| Statin users | Concentration of Medium LDL | 23537 | Adjusted GRS (1 SD) | 1.3773E-07 | 4.4038E-07 | 0.31 | 7.54E-01 | 1.00E+00 |
| Non-statin users | Concentration of Medium LDL | 23537 | GRS (1 SD) | -3.823E-06 | 2.4001E-07 | -15.93 | 4.99E-57 | 1.14E-54 |
| Non-statin users | Concentration of Medium LDL | 23537 | Adjusted GRS (1 SD) | 8.8823E-08 | 2.3867E-07 | 0.37 | 7.10E-01 | 1.00E+00 |
| All | Concentration of Medium LDL | 23537 | Statin dose (1 SD) | -1.051E-05 | 9.0934E-07 | -11.56 | 1.08E-30 | 2.46E-28 |
| All | Concentration of Medium LDL | 23537 | GRS (1 SD) | -2.802E-06 | 2.2177E-07 | -12.63 | 1.48E-36 | 3.38E-34 |
| All | Concentration of Medium LDL | 23537 | Adjusted GRS (1 SD) | 3.6235E-07 | 2.2032E-07 | 1.64 | 1.00E-01 | 1.00E+00 |
| Statin users | Total Lipids in Medium LDL | 23538 | Statin dose (1 SD) | -0.0076124 | 0.00158541 | -4.80 | 1.61E-06 | 3.66E-04 |
| Statin users | Total Lipids in Medium LDL | 23538 | GRS (1 SD) | -0.0050858 | 0.0010092 | -5.04 | 4.71E-07 | 1.07E-04 |
| Statin users | Total Lipids in Medium LDL | 23538 | Adjusted GRS (1 SD) | 0.00018167 | 0.00099683 | 0.18 | 8.55E-01 | 1.00E+00 |
| Non-statin users | Total Lipids in Medium LDL | 23538 | GRS (1 SD) | -0.0101848 | 0.00052373 | -19.45 | 4.67E-84 | 1.07E-81 |
| Non-statin users | Total Lipids in Medium LDL | 23538 | Adjusted GRS (1 SD) | 0.00016027 | 0.00052116 | 0.31 | 7.58E-01 | 1.00E+00 |
| All | Total Lipids in Medium LDL | 23538 | Statin dose (1 SD) | -0.0212938 | 0.0020026 | -10.63 | 2.99E-26 | 6.83E-24 |
| All | Total Lipids in Medium LDL | 23538 | GRS (1 SD) | -0.0079054 | 0.00048125 | -16.43 | 1.44E-60 | 3.28E-58 |
| All | Total Lipids in Medium LDL | 23538 | Adjusted GRS (1 SD) | 0.00071093 | 0.00047831 | 1.49 | 1.37E-01 | 1.00E+00 |
| Statin users | Phospholipids in Medium LDL | 23539 | Statin dose (1 SD) | -0.001853 | 0.00039765 | -4.66 | 3.22E-06 | 7.34E-04 |
| Statin users | Phospholipids in Medium LDL | 23539 | GRS (1 SD) | -0.0015009 | 0.00025346 | -5.92 | 3.24E-09 | 7.39E-07 |

| collective | metabolite | field | variable | Estimate | Std. Error | t value | p value | p value Bonferroni |
|---|---|---|---|---|---|---|---|---|
| Statin users | Phospholipids in Medium LDL | 23539 | Adjusted GRS (1 SD) | 2.8799E-05 | 0.00025041 | 0.12 | 9.08E-01 | 1.00E+00 |
| Non-statin users | Phospholipids in Medium LDL | 23539 | GRS (1 SD) | -0.0029454 | 0.00013346 | -22.07 | 1.21E-107 | 2.75E-105 |
| Non-statin users | Phospholipids in Medium LDL | 23539 | Adjusted GRS (1 SD) | 2.025E-05 | 0.00013289 | 0.15 | 8.79E-01 | 1.00E+00 |
| All | Phospholipids in Medium LDL | 23539 | Statin dose (1 SD) | -0.0053304 | 0.00050505 | -10.55 | 6.89E-26 | 1.57E-23 |
| All | Phospholipids in Medium LDL | 23539 | GRS (1 SD) | -0.002342 | 0.00012227 | -19.15 | 1.19E-81 | 2.72E-79 |
| All | Phospholipids in Medium LDL | 23539 | Adjusted GRS (1 SD) | 0.00016073 | 0.00012157 | 1.32 | 1.86E-01 | 1.00E+00 |
| Statin users | Cholesterol in Medium LDL | 23540 | Statin dose (1 SD) | -0.0060057 | 0.00112412 | -5.34 | 9.44E-08 | 2.15E-05 |
| Statin users | Cholesterol in Medium LDL | 23540 | GRS (1 SD) | -0.0039462 | 0.00071505 | -5.52 | 3.46E-08 | 7.89E-06 |
| Statin users | Cholesterol in Medium LDL | 23540 | Adjusted GRS (1 SD) | 5.9604E-05 | 0.00070638 | 0.08 | 9.33E-01 | 1.00E+00 |
| Non-statin users | Cholesterol in Medium LDL | 23540 | GRS (1 SD) | -0.007321 | 0.0003714 | -19.71 | 2.61E-86 | 5.95E-84 |
| Non-statin users | Cholesterol in Medium LDL | 23540 | Adjusted GRS (1 SD) | 0.00010003 | 0.0003696 | 0.27 | 7.87E-01 | 1.00E+00 |
| All | Cholesterol in Medium LDL | 23540 | Statin dose (1 SD) | -0.0157974 | 0.00142179 | -11.11 | 1.70E-28 | 3.88E-26 |
| All | Cholesterol in Medium LDL | 23540 | GRS (1 SD) | -0.0057078 | 0.00034237 | -16.67 | 2.50E-62 | 5.70E-60 |
| All | Cholesterol in Medium LDL | 23540 | Adjusted GRS (1 SD) | 0.0004918 | 0.00034029 | 1.45 | 1.48E-01 | 1.00E+00 |
| Statin users | CE in Medium LDL | 23541 | Statin dose (1 SD) | -0.0042518 | 0.00084762 | -5.02 | 5.40E-07 | 1.23E-04 |
| Statin users | CE in Medium LDL | 23541 | GRS (1 SD) | -0.0020875 | 0.00053807 | -3.88 | 1.05E-04 | 2.39E-02 |
| Statin users | CE in Medium LDL | 23541 | Adjusted GRS (1 SD) | 0.00015055 | 0.00053134 | 0.28 | 7.77E-01 | 1.00E+00 |
| Non-statin users | CE in Medium LDL | 23541 | GRS (1 SD) | -0.0045936 | 0.00027783 | -16.53 | 2.59E-61 | 5.90E-59 |
| Non-statin users | CE in Medium LDL | 23541 | Adjusted GRS (1 SD) | 0.00012213 | 0.0002763 | 0.44 | 6.58E-01 | 1.00E+00 |
| All | CE in Medium LDL | 23541 | Statin dose (1 SD) | -0.0113736 | 0.00106225 | -10.71 | 1.36E-26 | 3.11E-24 |
| All | CE in Medium LDL | 23541 | GRS (1 SD) | -0.0034341 | 0.00025489 | -13.47 | 2.42E-41 | 5.53E-39 |
| All | CE in Medium LDL | 23541 | Adjusted GRS (1 SD) | 0.00040985 | 0.00025324 | 1.62 | 1.06E-01 | 1.00E+00 |
| Statin users | FC in Medium LDL | 23542 | Statin dose (1 SD) | -0.001754 | 0.00030214 | -5.81 | 6.70E-09 | 1.53E-06 |
| Statin users | FC in Medium LDL | 23542 | GRS (1 SD) | -0.0018587 | 0.00019309 | -9.63 | 6.97E-22 | 1.59E-19 |
| Statin users | FC in Medium LDL | 23542 | Adjusted GRS (1 SD) | -9.099E-05 | 0.00019106 | -0.48 | 6.34E-01 | 1.00E+00 |
| Non-statin users | FC in Medium LDL | 23542 | GRS (1 SD) | -0.0027274 | 0.00010117 | -26.96 | 2.03E-159 | 4.64E-157 |
| Non-statin users | FC in Medium LDL | 23542 | Adjusted GRS (1 SD) | -2.208E-05 | 0.00010087 | -0.22 | 8.27E-01 | 1.00E+00 |
| All | FC in Medium LDL | 23542 | Statin dose (1 SD) | -0.0044238 | 0.0003862 | -11.45 | 3.61E-30 | 8.23E-28 |

| collective | metabolite | field | variable | Estimate | Std. Error | t value | p value | p value Bonferroni |
|---|---|---|---|---|---|---|---|---|
| All | FC in Medium LDL | 23542 | GRS (1 SD) | -0.0022737 | 9.3916E-05 | -24.21 | 3.68E-129 | 8.40E-127 |
| All | FC in Medium LDL | 23542 | Adjusted GRS (1 SD) | 8.1957E-05 | 9.3469E-05 | 0.88 | 3.81E-01 | 1.00E+00 |
| Statin users | TG in Medium LDL | 23543 | Statin dose (1 SD) | 0.00024638 | 0.00011475 | 2.15 | 3.18E-02 | 1.00E+00 |
| Statin users | TG in Medium LDL | 23543 | GRS (1 SD) | 0.00036131 | 7.2884E-05 | 4.96 | 7.21E-07 | 1.64E-04 |
| Statin users | TG in Medium LDL | 23543 | Adjusted GRS (1 SD) | 9.3302E-05 | 7.1986E-05 | 1.30 | 1.95E-01 | 1.00E+00 |
| Non-statin users | TG in Medium LDL | 23543 | GRS (1 SD) | 8.1584E-05 | 3.3152E-05 | 2.46 | 1.39E-02 | 1.00E+00 |
| Non-statin users | TG in Medium LDL | 23543 | Adjusted GRS (1 SD) | 3.9989E-05 | 3.2919E-05 | 1.21 | 2.24E-01 | 1.00E+00 |
| All | TG in Medium LDL | 23543 | Statin dose (1 SD) | -0.000166 | 0.00013436 | -1.24 | 2.17E-01 | 1.00E+00 |
| All | TG in Medium LDL | 23543 | GRS (1 SD) | 0.00014444 | 2.9958E-05 | 4.82 | 1.43E-06 | 3.25E-04 |
| All | TG in Medium LDL | 23543 | Adjusted GRS (1 SD) | 5.8394E-05 | 2.9744E-05 | 1.96 | 4.96E-02 | 1.00E+00 |
| Statin users | Concentration of Small LDL | 23544 | Statin dose (1 SD) | -1.811E-06 | 3.4425E-07 | -5.26 | 1.47E-07 | 3.36E-05 |
| Statin users | Concentration of Small LDL | 23544 | GRS (1 SD) | -7.459E-07 | 2.1955E-07 | -3.40 | 6.81E-04 | 1.55E-01 |
| Statin users | Concentration of Small LDL | 23544 | Adjusted GRS (1 SD) | 2.0042E-07 | 2.1678E-07 | 0.92 | 3.55E-01 | 1.00E+00 |
| Non-statin users | Concentration of Small LDL | 23544 | GRS (1 SD) | -1.995E-06 | 1.1768E-07 | -16.95 | 2.28E-64 | 5.20E-62 |
| Non-statin users | Concentration of Small LDL | 23544 | Adjusted GRS (1 SD) | 5.7812E-08 | 1.1704E-07 | 0.49 | 6.21E-01 | 1.00E+00 |
| All | Concentration of Small LDL | 23544 | Statin dose (1 SD) | -5.276E-06 | 4.5504E-07 | -11.60 | 7.23E-31 | 1.65E-28 |
| All | Concentration of Small LDL | 23544 | GRS (1 SD) | -1.427E-06 | 1.096E-07 | -13.02 | 9.94E-39 | 2.27E-36 |
| All | Concentration of Small LDL | 23544 | Adjusted GRS (1 SD) | 2.1035E-07 | 1.0888E-07 | 1.93 | 5.34E-02 | 1.00E+00 |
| Statin users | Total Lipids in Small LDL | 23545 | Statin dose (1 SD) | -0.0029284 | 0.00061411 | -4.77 | 1.89E-06 | 4.31E-04 |
| Statin users | Total Lipids in Small LDL | 23545 | GRS (1 SD) | -0.0022524 | 0.00039107 | -5.76 | 8.56E-09 | 1.95E-06 |
| Statin users | Total Lipids in Small LDL | 23545 | Adjusted GRS (1 SD) | 0.00017092 | 0.00038635 | 0.44 | 6.58E-01 | 1.00E+00 |
| Non-statin users | Total Lipids in Small LDL | 23545 | GRS (1 SD) | -0.0044996 | 0.00020416 | -22.04 | 2.34E-107 | 5.33E-105 |
| Non-statin users | Total Lipids in Small LDL | 23545 | Adjusted GRS (1 SD) | 7.8589E-05 | 0.00020328 | 0.39 | 6.99E-01 | 1.00E+00 |
| All | Total Lipids in Small LDL | 23545 | Statin dose (1 SD) | -0.0087883 | 0.00079835 | -11.01 | 5.27E-28 | 1.20E-25 |
| All | Total Lipids in Small LDL | 23545 | GRS (1 SD) | -0.0035145 | 0.00019004 | -18.49 | 2.96E-76 | 6.74E-74 |
| All | Total Lipids in Small LDL | 23545 | Adjusted GRS (1 SD) | 0.00031514 | 0.00018893 | 1.67 | 9.53E-02 | 1.00E+00 |
| Statin users | Phospholipids in Small LDL | 23546 | Statin dose (1 SD) | -0.000675 | 0.00017478 | -3.86 | 1.13E-04 | 2.59E-02 |
| Statin users | Phospholipids in Small LDL | 23546 | GRS (1 SD) | -0.0009985 | 0.0001111 | -8.99 | 2.77E-19 | 6.33E-17 |

| collective | metabolite | field | variable | Estimate | Std. Error | t value | p value | p value Bonferroni |
|---|---|---|---|---|---|---|---|---|
| Statin users | Phospholipids in Small LDL | 23546 | Adjusted GRS (1 SD) | 4.3708E-05 | 0.0001099 | 0.40 | 6.91E-01 | 1.00E+00 |
| Non-statin users | Phospholipids in Small LDL | 23546 | GRS (1 SD) | -0.0016371 | 5.7996E-05 | -28.23 | 1.61E-174 | 3.68E-172 |
| Non-statin users | Phospholipids in Small LDL | 23546 | Adjusted GRS (1 SD) | 1.6088E-05 | 5.7848E-05 | 0.28 | 7.81E-01 | 1.00E+00 |
| All | Phospholipids in Small LDL | 23546 | Statin dose (1 SD) | -0.0023543 | 0.00022881 | -10.29 | 1.08E-24 | 2.47E-22 |
| All | Phospholipids in Small LDL | 23546 | GRS (1 SD) | -0.0013545 | 5.4151E-05 | -25.01 | 1.03E-137 | 2.34E-135 |
| All | Phospholipids in Small LDL | 23546 | Adjusted GRS (1 SD) | 8.3846E-05 | 5.3901E-05 | 1.56 | 1.20E-01 | 1.00E+00 |
| Statin users | Cholesterol in Small LDL | 23547 | Statin dose (1 SD) | -0.002311 | 0.00041215 | -5.61 | 2.13E-08 | 4.86E-06 |
| Statin users | Cholesterol in Small LDL | 23547 | GRS (1 SD) | -0.0015849 | 0.00026271 | -6.03 | 1.64E-09 | 3.74E-07 |
| Statin users | Cholesterol in Small LDL | 23547 | Adjusted GRS (1 SD) | 6.0771E-05 | 0.00025956 | 0.23 | 8.15E-01 | 1.00E+00 |
| Non-statin users | Cholesterol in Small LDL | 23547 | GRS (1 SD) | -0.0029994 | 0.00013856 | -21.65 | 1.19E-103 | 2.72E-101 |
| Non-statin users | Cholesterol in Small LDL | 23547 | Adjusted GRS (1 SD) | 3.9103E-05 | 0.00013795 | 0.28 | 7.77E-01 | 1.00E+00 |
| All | Cholesterol in Small LDL | 23547 | Statin dose (1 SD) | -0.0062813 | 0.00053637 | -11.71 | 1.90E-31 | 4.33E-29 |
| All | Cholesterol in Small LDL | 23547 | GRS (1 SD) | -0.002337 | 0.00012906 | -18.11 | 3.47E-73 | 7.91E-71 |
| All | Cholesterol in Small LDL | 23547 | Adjusted GRS (1 SD) | 0.00019708 | 0.0001283 | 1.54 | 1.25E-01 | 1.00E+00 |
| Statin users | CE in Small LDL | 23548 | Statin dose (1 SD) | -0.0016742 | 0.0003116 | -5.37 | 7.98E-08 | 1.82E-05 |
| Statin users | CE in Small LDL | 23548 | GRS (1 SD) | -0.0006641 | 0.00019864 | -3.34 | 8.29E-04 | 1.89E-01 |
| Statin users | CE in Small LDL | 23548 | Adjusted GRS (1 SD) | 9.757E-05 | 0.00019613 | 0.50 | 6.19E-01 | 1.00E+00 |
| Non-statin users | CE in Small LDL | 23548 | GRS (1 SD) | -0.0017747 | 0.00010426 | -17.02 | 7.25E-65 | 1.65E-62 |
| Non-statin users | CE in Small LDL | 23548 | Adjusted GRS (1 SD) | 4.9246E-05 | 0.0001037 | 0.47 | 6.35E-01 | 1.00E+00 |
| All | CE in Small LDL | 23548 | Statin dose (1 SD) | -0.0046192 | 0.00040274 | -11.47 | 3.05E-30 | 6.95E-28 |
| All | CE in Small LDL | 23548 | GRS (1 SD) | -0.0012843 | 9.6621E-05 | -13.29 | 2.73E-40 | 6.23E-38 |
| All | CE in Small LDL | 23548 | Adjusted GRS (1 SD) | 0.00016818 | 9.5992E-05 | 1.75 | 7.98E-02 | 1.00E+00 |
| Statin users | FC in Small LDL | 23549 | Statin dose (1 SD) | -0.0006368 | 0.00011847 | -5.38 | 7.89E-08 | 1.80E-05 |
| Statin users | FC in Small LDL | 23549 | GRS (1 SD) | -0.0009208 | 7.5204E-05 | -12.24 | 2.43E-34 | 5.53E-32 |
| Statin users | FC in Small LDL | 23549 | Adjusted GRS (1 SD) | -3.681E-05 | 7.452E-05 | -0.49 | 6.21E-01 | 1.00E+00 |
| Non-statin users | FC in Small LDL | 23549 | GRS (1 SD) | -0.0012247 | 3.891E-05 | -31.47 | 3.08E-216 | 7.02E-214 |
| Non-statin users | FC in Small LDL | 23549 | Adjusted GRS (1 SD) | -1.015E-05 | 3.8854E-05 | -0.26 | 7.94E-01 | 1.00E+00 |
| All | FC in Small LDL | 23549 | Statin dose (1 SD) | -0.0016621 | 0.00015165 | -10.96 | 8.93E-28 | 2.04E-25 |

| collective | metabolite | field | variable | Estimate | Std. Error | t value | p value | p value Bonferroni |
|---|---|---|---|---|---|---|---|---|
| All | FC in Small LDL | 23549 | GRS (1 SD) | -0.0010527 | 3.6386E-05 | -28.93 | 2.12E-183 | 4.83E-181 |
| All | FC in Small LDL | 23549 | Adjusted GRS (1 SD) | 2.8895E-05 | 3.6251E-05 | 0.80 | 4.25E-01 | 1.00E+00 |
| Statin users | TG in Small LDL | 23550 | Statin dose (1 SD) | 5.7661E-05 | 6.6618E-05 | 0.87 | 3.87E-01 | 1.00E+00 |
| Statin users | TG in Small LDL | 23550 | GRS (1 SD) | 0.00033103 | 4.1808E-05 | 7.92 | 2.54E-15 | 5.80E-13 |
| Statin users | TG in Small LDL | 23550 | Adjusted GRS (1 SD) | 6.6454E-05 | 4.1332E-05 | 1.61 | 1.08E-01 | 1.00E+00 |
| Non-statin users | TG in Small LDL | 23550 | GRS (1 SD) | 0.00013694 | 1.8136E-05 | 7.55 | 4.37E-14 | 9.96E-12 |
| Non-statin users | TG in Small LDL | 23550 | Adjusted GRS (1 SD) | 2.3426E-05 | 1.8014E-05 | 1.30 | 1.93E-01 | 1.00E+00 |
| All | TG in Small LDL | 23550 | Statin dose (1 SD) | -0.0001527 | 7.6433E-05 | -2.00 | 4.57E-02 | 1.00E+00 |
| All | TG in Small LDL | 23550 | GRS (1 SD) | 0.00017698 | 1.6505E-05 | 10.72 | 8.18E-27 | 1.87E-24 |
| All | TG in Small LDL | 23550 | Adjusted GRS (1 SD) | 3.4231E-05 | 1.6393E-05 | 2.09 | 3.68E-02 | 1.00E+00 |
| Statin users | Concentration of Very Large HDL | 23551 | Statin dose (1 SD) | -1.547E-06 | 8.9386E-07 | -1.73 | 8.35E-02 | 1.00E+00 |
| Statin users | Concentration of Very Large HDL | 23551 | GRS (1 SD) | -1.84E-06 | 5.6746E-07 | -3.24 | 1.19E-03 | 2.71E-01 |
| Statin users | Concentration of Very Large HDL | 23551 | Adjusted GRS (1 SD) | 2.9895E-08 | 5.6029E-07 | 0.05 | 9.57E-01 | 1.00E+00 |
| Non-statin users | Concentration of Very Large HDL | 23551 | GRS (1 SD) | -1.474E-06 | 2.9566E-07 | -4.98 | 6.21E-07 | 1.42E-04 |
| Non-statin users | Concentration of Very Large HDL | 23551 | Adjusted GRS (1 SD) | 1.6737E-07 | 2.9363E-07 | 0.57 | 5.69E-01 | 1.00E+00 |
| All | Concentration of Very Large HDL | 23551 | Statin dose (1 SD) | -5.104E-06 | 9.8654E-07 | -5.17 | 2.35E-07 | 5.36E-05 |
| All | Concentration of Very Large HDL | 23551 | GRS (1 SD) | -1.009E-06 | 2.574E-07 | -3.92 | 8.90E-05 | 2.03E-02 |
| All | Concentration of Very Large HDL | 23551 | Adjusted GRS (1 SD) | 2.994E-07 | 2.5555E-07 | 1.17 | 2.41E-01 | 1.00E+00 |
| Statin users | Total Lipids in Very Large HDL | 23552 | Statin dose (1 SD) | -0.0012628 | 0.00075495 | -1.67 | 9.44E-02 | 1.00E+00 |
| Statin users | Total Lipids in Very Large HDL | 23552 | GRS (1 SD) | -0.0017029 | 0.0004783 | -3.56 | 3.71E-04 | 8.47E-02 |
| Statin users | Total Lipids in Very Large HDL | 23552 | Adjusted GRS (1 SD) | -8.688E-05 | 0.00047228 | -0.18 | 8.54E-01 | 1.00E+00 |
| Non-statin users | Total Lipids in Very Large HDL | 23552 | GRS (1 SD) | -0.0010309 | 0.00024871 | -4.15 | 3.40E-05 | 7.75E-03 |
| Non-statin users | Total Lipids in Very Large HDL | 23552 | Adjusted GRS (1 SD) | 0.00012201 | 0.00024699 | 0.49 | 6.21E-01 | 1.00E+00 |
| All | Total Lipids in Very Large HDL | 23552 | Statin dose (1 SD) | -0.0036 | 0.00082215 | -4.38 | 1.21E-05 | 2.75E-03 |
| All | Total Lipids in Very Large HDL | 23552 | GRS (1 SD) | -0.0007633 | 0.00021527 | -3.55 | 3.91E-04 | 8.93E-02 |
| All | Total Lipids in Very Large HDL | 23552 | Adjusted GRS (1 SD) | 0.00019518 | 0.00021372 | 0.91 | 3.61E-01 | 1.00E+00 |
| Statin users | Phospholipids in Very Large HDL | 23553 | Statin dose (1 SD) | -0.0006093 | 0.00042307 | -1.44 | 1.50E-01 | 1.00E+00 |
| Statin users | Phospholipids in Very Large HDL | 23553 | GRS (1 SD) | -0.0007567 | 0.00026758 | -2.83 | 4.69E-03 | 1.00E+00 |

| collective | metabolite | field | variable | Estimate | Std. Error | t value | p value | p value Bonferroni |
|---|---|---|---|---|---|---|---|---|
| Statin users | Phospholipids in Very Large HDL | 23553 | Adjusted GRS (1 SD) | -4.651E-05 | 0.00026418 | -0.18 | 8.60E-01 | 1.00E+00 |
| Non-statin users | Phospholipids in Very Large HDL | 23553 | GRS (1 SD) | -0.0003196 | 0.00013982 | -2.29 | 2.23E-02 | 1.00E+00 |
| Non-statin users | Phospholipids in Very Large HDL | 23553 | Adjusted GRS (1 SD) | 6.6454E-05 | 0.00013884 | 0.48 | 6.32E-01 | 1.00E+00 |
| All | Phospholipids in Very Large HDL | 23553 | Statin dose (1 SD) | -0.0016816 | 0.00045849 | -3.67 | 2.46E-04 | 5.61E-02 |
| All | Phospholipids in Very Large HDL | 23553 | GRS (1 SD) | -0.0002069 | 0.00012039 | -1.72 | 8.58E-02 | 1.00E+00 |
| All | Phospholipids in Very Large HDL | 23553 | Adjusted GRS (1 SD) | 0.00010017 | 0.00011952 | 0.84 | 4.02E-01 | 1.00E+00 |
| Statin users | Cholesterol in Very Large HDL | 23554 | Statin dose (1 SD) | -0.0006771 | 0.00033175 | -2.04 | 4.13E-02 | 1.00E+00 |
| Statin users | Cholesterol in Very Large HDL | 23554 | GRS (1 SD) | -0.0010863 | 0.00021066 | -5.16 | 2.54E-07 | 5.78E-05 |
| Statin users | Cholesterol in Very Large HDL | 23554 | Adjusted GRS (1 SD) | -7.752E-05 | 0.00020808 | -0.37 | 7.10E-01 | 1.00E+00 |
| Non-statin users | Cholesterol in Very Large HDL | 23554 | GRS (1 SD) | -0.0007764 | 0.00010813 | -7.18 | 7.02E-13 | 1.60E-10 |
| Non-statin users | Cholesterol in Very Large HDL | 23554 | Adjusted GRS (1 SD) | 4.3603E-05 | 0.00010741 | 0.41 | 6.85E-01 | 1.00E+00 |
| All | Cholesterol in Very Large HDL | 23554 | Statin dose (1 SD) | -0.0018203 | 0.00036251 | -5.02 | 5.23E-07 | 1.19E-04 |
| All | Cholesterol in Very Large HDL | 23554 | GRS (1 SD) | -0.0006429 | 9.4324E-05 | -6.82 | 9.40E-12 | 2.14E-09 |
| All | Cholesterol in Very Large HDL | 23554 | Adjusted GRS (1 SD) | 7.5415E-05 | 9.3659E-05 | 0.81 | 4.21E-01 | 1.00E+00 |
| Statin users | CE in Very Large HDL | 23555 | Statin dose (1 SD) | -0.0005071 | 0.00026373 | -1.92 | 5.46E-02 | 1.00E+00 |
| Statin users | CE in Very Large HDL | 23555 | GRS (1 SD) | -0.0008174 | 0.00016744 | -4.88 | 1.06E-06 | 2.42E-04 |
| Statin users | CE in Very Large HDL | 23555 | Adjusted GRS (1 SD) | -8.054E-05 | 0.00016538 | -0.49 | 6.26E-01 | 1.00E+00 |
| Non-statin users | CE in Very Large HDL | 23555 | GRS (1 SD) | -0.000554 | 8.628E-05 | -6.42 | 1.36E-10 | 3.11E-08 |
| Non-statin users | CE in Very Large HDL | 23555 | Adjusted GRS (1 SD) | 3.016E-05 | 8.5693E-05 | 0.35 | 7.25E-01 | 1.00E+00 |
| All | CE in Very Large HDL | 23555 | Statin dose (1 SD) | -0.0013314 | 0.0002871 | -4.64 | 3.58E-06 | 8.16E-04 |
| All | CE in Very Large HDL | 23555 | GRS (1 SD) | -0.0004573 | 7.5029E-05 | -6.10 | 1.09E-09 | 2.49E-07 |
| All | CE in Very Large HDL | 23555 | Adjusted GRS (1 SD) | 5.2117E-05 | 7.4497E-05 | 0.70 | 4.84E-01 | 1.00E+00 |
| Statin users | FC in Very Large HDL | 23556 | Statin dose (1 SD) | -0.00017 | 7.1551E-05 | -2.38 | 1.75E-02 | 1.00E+00 |
| Statin users | FC in Very Large HDL | 23556 | GRS (1 SD) | -0.0002689 | 4.5437E-05 | -5.92 | 3.30E-09 | 7.53E-07 |
| Statin users | FC in Very Large HDL | 23556 | Adjusted GRS (1 SD) | 3.0371E-06 | 4.4891E-05 | 0.07 | 9.46E-01 | 1.00E+00 |
| Non-statin users | FC in Very Large HDL | 23556 | GRS (1 SD) | -0.0002224 | 2.2817E-05 | -9.75 | 1.92E-22 | 4.38E-20 |
| Non-statin users | FC in Very Large HDL | 23556 | Adjusted GRS (1 SD) | 1.3447E-05 | 2.2669E-05 | 0.59 | 5.53E-01 | 1.00E+00 |
| All | FC in Very Large HDL | 23556 | Statin dose (1 SD) | -0.0004889 | 7.9436E-05 | -6.15 | 7.83E-10 | 1.79E-07 |

| collective | metabolite | field | variable | Estimate | Std. Error | t value | p value | p value Bonferroni |
|---|---|---|---|---|---|---|---|---|
| All | FC in Very Large HDL | 23556 | GRS (1 SD) | -0.0001856 | 2.0149E-05 | -9.21 | 3.31E-20 | 7.54E-18 |
| All | FC in Very Large HDL | 23556 | Adjusted GRS (1 SD) | 2.3303E-05 | 2.001E-05 | 1.16 | 2.44E-01 | 1.00E+00 |
| Statin users | TG in Very Large HDL | 23557 | Statin dose (1 SD) | 2.3603E-05 | 3.0231E-05 | 0.78 | 4.35E-01 | 1.00E+00 |
| Statin users | TG in Very Large HDL | 23557 | GRS (1 SD) | 0.00014008 | 1.8979E-05 | 7.38 | 1.63E-13 | 3.73E-11 |
| Statin users | TG in Very Large HDL | 23557 | Adjusted GRS (1 SD) | 3.7119E-05 | 1.8758E-05 | 1.98 | 4.78E-02 | 1.00E+00 |
| Non-statin users | TG in Very Large HDL | 23557 | GRS (1 SD) | 6.5033E-05 | 8.7467E-06 | 7.44 | 1.05E-13 | 2.40E-11 |
| Non-statin users | TG in Very Large HDL | 23557 | Adjusted GRS (1 SD) | 1.1946E-05 | 8.6878E-06 | 1.38 | 1.69E-01 | 1.00E+00 |
| All | TG in Very Large HDL | 23557 | Statin dose (1 SD) | -9.813E-05 | 3.5267E-05 | -2.78 | 5.40E-03 | 1.00E+00 |
| All | TG in Very Large HDL | 23557 | GRS (1 SD) | 8.6468E-05 | 7.8324E-06 | 11.04 | 2.54E-28 | 5.79E-26 |
| All | TG in Very Large HDL | 23557 | Adjusted GRS (1 SD) | 1.9592E-05 | 7.7795E-06 | 2.52 | 1.18E-02 | 1.00E+00 |
| Statin users | Concentration of Large HDL | 23558 | Statin dose (1 SD) | -1.282E-05 | 7.2961E-06 | -1.76 | 7.88E-02 | 1.00E+00 |
| Statin users | Concentration of Large HDL | 23558 | GRS (1 SD) | -8.776E-06 | 4.6207E-06 | -1.90 | 5.75E-02 | 1.00E+00 |
| Statin users | Concentration of Large HDL | 23558 | Adjusted GRS (1 SD) | -3.489E-06 | 4.5614E-06 | -0.76 | 4.44E-01 | 1.00E+00 |
| Non-statin users | Concentration of Large HDL | 23558 | GRS (1 SD) | -1.091E-07 | 2.3608E-06 | -0.05 | 9.63E-01 | 1.00E+00 |
| Non-statin users | Concentration of Large HDL | 23558 | Adjusted GRS (1 SD) | 5.4485E-07 | 2.3442E-06 | 0.23 | 8.16E-01 | 1.00E+00 |
| All | Concentration of Large HDL | 23558 | Statin dose (1 SD) | -2.305E-05 | 7.8587E-06 | -2.93 | 3.37E-03 | 7.68E-01 |
| All | Concentration of Large HDL | 23558 | GRS (1 SD) | 1.005E-06 | 2.0305E-06 | 0.49 | 6.21E-01 | 1.00E+00 |
| All | Concentration of Large HDL | 23558 | Adjusted GRS (1 SD) | 7.4766E-07 | 2.0158E-06 | 0.37 | 7.11E-01 | 1.00E+00 |
| Statin users | Total Lipids in Large HDL | 23559 | Statin dose (1 SD) | -0.0052457 | 0.00307274 | -1.71 | 8.78E-02 | 1.00E+00 |
| Statin users | Total Lipids in Large HDL | 23559 | GRS (1 SD) | -0.0036125 | 0.00194433 | -1.86 | 6.32E-02 | 1.00E+00 |
| Statin users | Total Lipids in Large HDL | 23559 | Adjusted GRS (1 SD) | -0.0016032 | 0.00191937 | -0.84 | 4.04E-01 | 1.00E+00 |
| Non-statin users | Total Lipids in Large HDL | 23559 | GRS (1 SD) | 0.00025133 | 0.00099593 | 0.25 | 8.01E-01 | 1.00E+00 |
| Non-statin users | Total Lipids in Large HDL | 23559 | Adjusted GRS (1 SD) | 0.00021129 | 0.00098892 | 0.21 | 8.31E-01 | 1.00E+00 |
| All | Total Lipids in Large HDL | 23559 | Statin dose (1 SD) | -0.0083193 | 0.00330907 | -2.51 | 1.20E-02 | 1.00E+00 |
| All | Total Lipids in Large HDL | 23559 | GRS (1 SD) | 0.00051442 | 0.00085434 | 0.60 | 5.47E-01 | 1.00E+00 |
| All | Total Lipids in Large HDL | 23559 | Adjusted GRS (1 SD) | 0.00023125 | 0.00084815 | 0.27 | 7.85E-01 | 1.00E+00 |
| Statin users | Phospholipids in Large HDL | 23560 | Statin dose (1 SD) | -0.0024689 | 0.00145497 | -1.70 | 8.98E-02 | 1.00E+00 |
| Statin users | Phospholipids in Large HDL | 23560 | GRS (1 SD) | -0.0013001 | 0.0009202 | -1.41 | 1.58E-01 | 1.00E+00 |

| collective | metabolite | field | variable | Estimate | Std. Error | t value | p value | p value Bonferroni |
|---|---|---|---|---|---|---|---|---|
| Statin users | Phospholipids in Large HDL | 23560 | Adjusted GRS (1 SD) | -0.0007988 | 0.00090835 | -0.88 | 3.79E-01 | 1.00E+00 |
| Non-statin users | Phospholipids in Large HDL | 23560 | GRS (1 SD) | 0.00049794 | 0.00046857 | 1.06 | 2.88E-01 | 1.00E+00 |
| Non-statin users | Phospholipids in Large HDL | 23560 | Adjusted GRS (1 SD) | 0.00010835 | 0.00046527 | 0.23 | 8.16E-01 | 1.00E+00 |
| All | Phospholipids in Large HDL | 23560 | Statin dose (1 SD) | -0.0033228 | 0.00156814 | -2.12 | 3.41E-02 | 1.00E+00 |
| All | Phospholipids in Large HDL | 23560 | GRS (1 SD) | 0.0005348 | 0.00040139 | 1.33 | 1.83E-01 | 1.00E+00 |
| All | Phospholipids in Large HDL | 23560 | Adjusted GRS (1 SD) | 9.1215E-05 | 0.00039849 | 0.23 | 8.19E-01 | 1.00E+00 |
| Statin users | Cholesterol in Large HDL | 23561 | Statin dose (1 SD) | -0.0027865 | 0.00159158 | -1.75 | 8.00E-02 | 1.00E+00 |
| Statin users | Cholesterol in Large HDL | 23561 | GRS (1 SD) | -0.0029725 | 0.00100881 | -2.95 | 3.22E-03 | 7.34E-01 |
| Statin users | Cholesterol in Large HDL | 23561 | Adjusted GRS (1 SD) | -0.0009333 | 0.00099599 | -0.94 | 3.49E-01 | 1.00E+00 |
| Non-statin users | Cholesterol in Large HDL | 23561 | GRS (1 SD) | -0.0007148 | 0.00051739 | -1.38 | 1.67E-01 | 1.00E+00 |
| Non-statin users | Cholesterol in Large HDL | 23561 | Adjusted GRS (1 SD) | 6.6843E-05 | 0.00051375 | 0.13 | 8.96E-01 | 1.00E+00 |
| All | Cholesterol in Large HDL | 23561 | Statin dose (1 SD) | -0.0047128 | 0.00171313 | -2.75 | 5.95E-03 | 1.00E+00 |
| All | Cholesterol in Large HDL | 23561 | GRS (1 SD) | -0.0005428 | 0.00044513 | -1.22 | 2.23E-01 | 1.00E+00 |
| All | Cholesterol in Large HDL | 23561 | Adjusted GRS (1 SD) | 7.5139E-05 | 0.00044191 | 0.17 | 8.65E-01 | 1.00E+00 |
| Statin users | CE in Large HDL | 23562 | Statin dose (1 SD) | -0.0020956 | 0.00124006 | -1.69 | 9.11E-02 | 1.00E+00 |
| Statin users | CE in Large HDL | 23562 | GRS (1 SD) | -0.0023525 | 0.00078606 | -2.99 | 2.77E-03 | 6.31E-01 |
| Statin users | CE in Large HDL | 23562 | Adjusted GRS (1 SD) | -0.0007679 | 0.00077607 | -0.99 | 3.22E-01 | 1.00E+00 |
| Non-statin users | CE in Large HDL | 23562 | GRS (1 SD) | -0.0004656 | 0.0004029 | -1.16 | 2.48E-01 | 1.00E+00 |
| Non-statin users | CE in Large HDL | 23562 | Adjusted GRS (1 SD) | 3.6408E-05 | 0.00040007 | 0.09 | 9.27E-01 | 1.00E+00 |
| All | CE in Large HDL | 23562 | Statin dose (1 SD) | -0.0033908 | 0.00133453 | -2.54 | 1.11E-02 | 1.00E+00 |
| All | CE in Large HDL | 23562 | GRS (1 SD) | -0.0003659 | 0.00034665 | -1.06 | 2.91E-01 | 1.00E+00 |
| All | CE in Large HDL | 23562 | Adjusted GRS (1 SD) | 3.1598E-05 | 0.00034414 | 0.09 | 9.27E-01 | 1.00E+00 |
| Statin users | FC in Large HDL | 23563 | Statin dose (1 SD) | -0.000691 | 0.00035599 | -1.94 | 5.23E-02 | 1.00E+00 |
| Statin users | FC in Large HDL | 23563 | GRS (1 SD) | -0.0006199 | 0.00022553 | -2.75 | 5.99E-03 | 1.00E+00 |
| Statin users | FC in Large HDL | 23563 | Adjusted GRS (1 SD) | -0.0001653 | 0.00022266 | -0.74 | 4.58E-01 | 1.00E+00 |
| Non-statin users | FC in Large HDL | 23563 | GRS (1 SD) | -0.0002492 | 0.00011563 | -2.16 | 3.11E-02 | 1.00E+00 |
| Non-statin users | FC in Large HDL | 23563 | Adjusted GRS (1 SD) | 3.0428E-05 | 0.00011482 | 0.26 | 7.91E-01 | 1.00E+00 |
| All | FC in Large HDL | 23563 | Statin dose (1 SD) | -0.0013221 | 0.00038392 | -3.44 | 5.77E-04 | 1.31E-01 |

| collective | metabolite | field | variable | Estimate | Std. Error | t value | p value | p value Bonferroni |
|---|---|---|---|---|---|---|---|---|
| All | FC in Large HDL | 23563 | GRS (1 SD) | -0.0001769 | 9.953E-05 | -1.78 | 7.56E-02 | 1.00E+00 |
| All | FC in Large HDL | 23563 | Adjusted GRS (1 SD) | 4.3544E-05 | 9.881E-05 | 0.44 | 6.59E-01 | 1.00E+00 |
| Statin users | TG in Large HDL | 23564 | Statin dose (1 SD) | 1.0025E-05 | 0.00013187 | 0.08 | 9.39E-01 | 1.00E+00 |
| Statin users | TG in Large HDL | 23564 | GRS (1 SD) | 0.00066 | 8.215E-05 | 8.03 | 9.96E-16 | 2.27E-13 |
| Statin users | TG in Large HDL | 23564 | Adjusted GRS (1 SD) | 0.00012882 | 8.122E-05 | 1.59 | 1.13E-01 | 1.00E+00 |
| Non-statin users | TG in Large HDL | 23564 | GRS (1 SD) | 0.00046824 | 3.9737E-05 | 11.78 | 5.02E-32 | 1.15E-29 |
| Non-statin users | TG in Large HDL | 23564 | Adjusted GRS (1 SD) | 3.6182E-05 | 3.9488E-05 | 0.92 | 3.60E-01 | 1.00E+00 |
| All | TG in Large HDL | 23564 | Statin dose (1 SD) | -0.0002836 | 0.00014865 | -1.91 | 5.65E-02 | 1.00E+00 |
| All | TG in Large HDL | 23564 | GRS (1 SD) | 0.00052246 | 3.4644E-05 | 15.08 | 2.43E-51 | 5.54E-49 |
| All | TG in Large HDL | 23564 | Adjusted GRS (1 SD) | 6.4977E-05 | 3.4426E-05 | 1.89 | 5.91E-02 | 1.00E+00 |
| Statin users | Concentration of Medium HDL | 23565 | Statin dose (1 SD) | -2.304E-05 | 1.0126E-05 | -2.28 | 2.29E-02 | 1.00E+00 |
| Statin users | Concentration of Medium HDL | 23565 | GRS (1 SD) | 1.1622E-05 | 6.3873E-06 | 1.82 | 6.88E-02 | 1.00E+00 |
| Statin users | Concentration of Medium HDL | 23565 | Adjusted GRS (1 SD) | -7.495E-06 | 6.3052E-06 | -1.19 | 2.35E-01 | 1.00E+00 |
| Non-statin users | Concentration of Medium HDL | 23565 | GRS (1 SD) | 1.6537E-05 | 2.8837E-06 | 5.73 | 9.80E-09 | 2.23E-06 |
| Non-statin users | Concentration of Medium HDL | 23565 | Adjusted GRS (1 SD) | 3.9547E-07 | 2.864E-06 | 0.14 | 8.90E-01 | 1.00E+00 |
| All | Concentration of Medium HDL | 23565 | Statin dose (1 SD) | -2.07E-05 | 1.0914E-05 | -1.90 | 5.78E-02 | 1.00E+00 |
| All | Concentration of Medium HDL | 23565 | GRS (1 SD) | 1.6517E-05 | 2.518E-06 | 6.56 | 5.42E-11 | 1.24E-08 |
| All | Concentration of Medium HDL | 23565 | Adjusted GRS (1 SD) | -7.941E-08 | 2.5002E-06 | -0.03 | 9.75E-01 | 1.00E+00 |
| Statin users | Total Lipids in Medium HDL | 23566 | Statin dose (1 SD) | -0.0050581 | 0.00249849 | -2.02 | 4.30E-02 | 1.00E+00 |
| Statin users | Total Lipids in Medium HDL | 23566 | GRS (1 SD) | 0.0038722 | 0.00157265 | 2.46 | 1.38E-02 | 1.00E+00 |
| Statin users | Total Lipids in Medium HDL | 23566 | Adjusted GRS (1 SD) | -0.0017468 | 0.00155254 | -1.13 | 2.61E-01 | 1.00E+00 |
| Non-statin users | Total Lipids in Medium HDL | 23566 | GRS (1 SD) | 0.00484802 | 0.0007016 | 6.91 | 4.88E-12 | 1.11E-09 |
| Non-statin users | Total Lipids in Medium HDL | 23566 | Adjusted GRS (1 SD) | 0.00015236 | 0.00069686 | 0.22 | 8.27E-01 | 1.00E+00 |
| All | Total Lipids in Medium HDL | 23566 | Statin dose (1 SD) | -0.0038585 | 0.00269856 | -1.43 | 1.53E-01 | 1.00E+00 |
| All | Total Lipids in Medium HDL | 23566 | GRS (1 SD) | 0.00474191 | 0.00061434 | 7.72 | 1.18E-14 | 2.70E-12 |
| All | Total Lipids in Medium HDL | 23566 | Adjusted GRS (1 SD) | 7.5977E-06 | 0.00061004 | 0.01 | 9.90E-01 | 1.00E+00 |
| Statin users | Phospholipids in Medium HDL | 23567 | Statin dose (1 SD) | -0.0022713 | 0.00113387 | -2.00 | 4.52E-02 | 1.00E+00 |
| Statin users | Phospholipids in Medium HDL | 23567 | GRS (1 SD) | 0.00245877 | 0.00071169 | 3.45 | 5.52E-04 | 1.26E-01 |

| collective | metabolite | field | variable | Estimate | Std. Error | t value | p value | p value Bonferroni |
|---|---|---|---|---|---|---|---|---|
| Statin users | Phospholipids in Medium HDL | 23567 | Adjusted GRS (1 SD) | -0.0007067 | 0.0007027 | -1.01 | 3.15E-01 | 1.00E+00 |
| Non-statin users | Phospholipids in Medium HDL | 23567 | GRS (1 SD) | 0.00266608 | 0.00031416 | 8.49 | 2.17E-17 | 4.94E-15 |
| Non-statin users | Phospholipids in Medium HDL | 23567 | Adjusted GRS (1 SD) | 0.00010261 | 0.00031208 | 0.33 | 7.42E-01 | 1.00E+00 |
| All | Phospholipids in Medium HDL | 23567 | Statin dose (1 SD) | -0.0016631 | 0.00122748 | -1.35 | 1.75E-01 | 1.00E+00 |
| All | Phospholipids in Medium HDL | 23567 | GRS (1 SD) | 0.00261495 | 0.00027596 | 9.48 | 2.70E-21 | 6.15E-19 |
| All | Phospholipids in Medium HDL | 23567 | Adjusted GRS (1 SD) | 3.5294E-05 | 0.00027407 | 0.13 | 8.98E-01 | 1.00E+00 |
| Statin users | Cholesterol in Medium HDL | 23568 | Statin dose (1 SD) | -0.0027912 | 0.0013188 | -2.12 | 3.43E-02 | 1.00E+00 |
| Statin users | Cholesterol in Medium HDL | 23568 | GRS (1 SD) | 7.3714E-05 | 0.00083505 | 0.09 | 9.30E-01 | 1.00E+00 |
| Statin users | Cholesterol in Medium HDL | 23568 | Adjusted GRS (1 SD) | -0.0012216 | 0.00082423 | -1.48 | 1.38E-01 | 1.00E+00 |
| Non-statin users | Cholesterol in Medium HDL | 23568 | GRS (1 SD) | 0.00127183 | 0.00038055 | 3.34 | 8.32E-04 | 1.90E-01 |
| Non-statin users | Cholesterol in Medium HDL | 23568 | Adjusted GRS (1 SD) | -1.02E-05 | 0.0003779 | -0.03 | 9.78E-01 | 1.00E+00 |
| All | Cholesterol in Medium HDL | 23568 | Statin dose (1 SD) | -0.0018989 | 0.00142282 | -1.33 | 1.82E-01 | 1.00E+00 |
| All | Cholesterol in Medium HDL | 23568 | GRS (1 SD) | 0.0011514 | 0.00033183 | 3.47 | 5.21E-04 | 1.19E-01 |
| All | Cholesterol in Medium HDL | 23568 | Adjusted GRS (1 SD) | -0.0001184 | 0.00032944 | -0.36 | 7.19E-01 | 1.00E+00 |
| Statin users | CE in Medium HDL | 23569 | Statin dose (1 SD) | -0.0021563 | 0.00106181 | -2.03 | 4.23E-02 | 1.00E+00 |
| Statin users | CE in Medium HDL | 23569 | GRS (1 SD) | 2.3922E-05 | 0.00067256 | 0.04 | 9.72E-01 | 1.00E+00 |
| Statin users | CE in Medium HDL | 23569 | Adjusted GRS (1 SD) | -0.0010321 | 0.00066384 | -1.55 | 1.20E-01 | 1.00E+00 |
| Non-statin users | CE in Medium HDL | 23569 | GRS (1 SD) | 0.00112463 | 0.0003061 | 3.67 | 2.39E-04 | 5.45E-02 |
| Non-statin users | CE in Medium HDL | 23569 | Adjusted GRS (1 SD) | -2.703E-05 | 0.00030397 | -0.09 | 9.29E-01 | 1.00E+00 |
| All | CE in Medium HDL | 23569 | Statin dose (1 SD) | -0.0011272 | 0.00114741 | -0.98 | 3.26E-01 | 1.00E+00 |
| All | CE in Medium HDL | 23569 | GRS (1 SD) | 0.00097938 | 0.00026711 | 3.67 | 2.46E-04 | 5.61E-02 |
| All | CE in Medium HDL | 23569 | Adjusted GRS (1 SD) | -0.0001292 | 0.00026519 | -0.49 | 6.26E-01 | 1.00E+00 |
| Statin users | FC in Medium HDL | 23570 | Statin dose (1 SD) | -0.000635 | 0.00026289 | -2.42 | 1.57E-02 | 1.00E+00 |
| Statin users | FC in Medium HDL | 23570 | GRS (1 SD) | 4.9769E-05 | 0.0001662 | 0.30 | 7.65E-01 | 1.00E+00 |
| Statin users | FC in Medium HDL | 23570 | Adjusted GRS (1 SD) | -0.0001896 | 0.00016405 | -1.16 | 2.48E-01 | 1.00E+00 |
| Non-statin users | FC in Medium HDL | 23570 | GRS (1 SD) | 0.00014723 | 7.6253E-05 | 1.93 | 5.35E-02 | 1.00E+00 |
| Non-statin users | FC in Medium HDL | 23570 | Adjusted GRS (1 SD) | 1.6856E-05 | 7.5718E-05 | 0.22 | 8.24E-01 | 1.00E+00 |
| All | FC in Medium HDL | 23570 | Statin dose (1 SD) | -0.0007717 | 0.00028306 | -2.73 | 6.42E-03 | 1.00E+00 |

| collective | metabolite | field | variable | Estimate | Std. Error | t value | p value | p value Bonferroni |
|---|---|---|---|---|---|---|---|---|
| All | FC in Medium HDL | 23570 | GRS (1 SD) | 0.00017203 | 6.6402E-05 | 2.59 | 9.58E-03 | 1.00E+00 |
| All | FC in Medium HDL | 23570 | Adjusted GRS (1 SD) | 1.0807E-05 | 6.5923E-05 | 0.16 | 8.70E-01 | 1.00E+00 |
| Statin users | TG in Medium HDL | 23571 | Statin dose (1 SD) | 4.4569E-06 | 0.00021984 | 0.02 | 9.84E-01 | 1.00E+00 |
| Statin users | TG in Medium HDL | 23571 | GRS (1 SD) | 0.00133966 | 0.00013734 | 9.75 | 1.99E-22 | 4.53E-20 |
| Statin users | TG in Medium HDL | 23571 | Adjusted GRS (1 SD) | 0.00018136 | 0.00013589 | 1.33 | 1.82E-01 | 1.00E+00 |
| Non-statin users | TG in Medium HDL | 23571 | GRS (1 SD) | 0.00091002 | 6.2558E-05 | 14.55 | 6.95E-48 | 1.59E-45 |
| Non-statin users | TG in Medium HDL | 23571 | Adjusted GRS (1 SD) | 5.9876E-05 | 6.2192E-05 | 0.96 | 3.36E-01 | 1.00E+00 |
| All | TG in Medium HDL | 23571 | Statin dose (1 SD) | -0.0002964 | 0.00024505 | -1.21 | 2.27E-01 | 1.00E+00 |
| All | TG in Medium HDL | 23571 | GRS (1 SD) | 0.00097547 | 5.5408E-05 | 17.61 | 2.76E-69 | 6.29E-67 |
| All | TG in Medium HDL | 23571 | Adjusted GRS (1 SD) | 9.0669E-05 | 5.5078E-05 | 1.65 | 9.97E-02 | 1.00E+00 |
| Statin users | Concentration of Small HDL | 23572 | Statin dose (1 SD) | -3.973E-05 | 1.5424E-05 | -2.58 | 1.00E-02 | 1.00E+00 |
| Statin users | Concentration of Small HDL | 23572 | GRS (1 SD) | 2.4406E-06 | 9.7426E-06 | 0.25 | 8.02E-01 | 1.00E+00 |
| Statin users | Concentration of Small HDL | 23572 | Adjusted GRS (1 SD) | -1.085E-05 | 9.6166E-06 | -1.13 | 2.59E-01 | 1.00E+00 |
| Non-statin users | Concentration of Small HDL | 23572 | GRS (1 SD) | -1.456E-05 | 4.324E-06 | -3.37 | 7.59E-04 | 1.73E-01 |
| Non-statin users | Concentration of Small HDL | 23572 | Adjusted GRS (1 SD) | -6.608E-07 | 4.2939E-06 | -0.15 | 8.78E-01 | 1.00E+00 |
| All | Concentration of Small HDL | 23572 | Statin dose (1 SD) | -5.654E-05 | 1.6642E-05 | -3.40 | 6.84E-04 | 1.56E-01 |
| All | Concentration of Small HDL | 23572 | GRS (1 SD) | -9.859E-06 | 3.8027E-06 | -2.59 | 9.52E-03 | 1.00E+00 |
| All | Concentration of Small HDL | 23572 | Adjusted GRS (1 SD) | -6.023E-07 | 3.7752E-06 | -0.16 | 8.73E-01 | 1.00E+00 |
| Statin users | Total Lipids in Small HDL | 23573 | Statin dose (1 SD) | -0.0040285 | 0.00190458 | -2.12 | 3.45E-02 | 1.00E+00 |
| Statin users | Total Lipids in Small HDL | 23573 | GRS (1 SD) | 0.00381389 | 0.00119718 | 3.19 | 1.45E-03 | 3.30E-01 |
| Statin users | Total Lipids in Small HDL | 23573 | Adjusted GRS (1 SD) | -0.0008627 | 0.00118203 | -0.73 | 4.66E-01 | 1.00E+00 |
| Non-statin users | Total Lipids in Small HDL | 23573 | GRS (1 SD) | 0.00163968 | 0.00051877 | 3.16 | 1.57E-03 | 3.59E-01 |
| Non-statin users | Total Lipids in Small HDL | 23573 | Adjusted GRS (1 SD) | 9.6278E-05 | 0.00051515 | 0.19 | 8.52E-01 | 1.00E+00 |
| All | Total Lipids in Small HDL | 23573 | Statin dose (1 SD) | -0.0050363 | 0.00205078 | -2.46 | 1.41E-02 | 1.00E+00 |
| All | Total Lipids in Small HDL | 23573 | GRS (1 SD) | 0.00202482 | 0.00045874 | 4.41 | 1.02E-05 | 2.32E-03 |
| All | Total Lipids in Small HDL | 23573 | Adjusted GRS (1 SD) | 9.3831E-05 | 0.00045546 | 0.21 | 8.37E-01 | 1.00E+00 |
| Statin users | Phospholipids in Small HDL | 23574 | Statin dose (1 SD) | -0.002542 | 0.00113202 | -2.25 | 2.48E-02 | 1.00E+00 |
| Statin users | Phospholipids in Small HDL | 23574 | GRS (1 SD) | 0.00301589 | 0.00070973 | 4.25 | 2.15E-05 | 4.91E-03 |

| collective | metabolite | field | variable | Estimate | Std. Error | t value | p value | p value Bonferroni |
|---|---|---|---|---|---|---|---|---|
| Statin users | Phospholipids in Small HDL | 23574 | Adjusted GRS (1 SD) | -0.000453 | 0.00070089 | -0.65 | 5.18E-01 | 1.00E+00 |
| Non-statin users | Phospholipids in Small HDL | 23574 | GRS (1 SD) | 0.00188818 | 0.00030383 | 6.21 | 5.17E-10 | 1.18E-07 |
| Non-statin users | Phospholipids in Small HDL | 23574 | Adjusted GRS (1 SD) | 9.5981E-05 | 0.00030176 | 0.32 | 7.50E-01 | 1.00E+00 |
| All | Phospholipids in Small HDL | 23574 | Statin dose (1 SD) | -0.0027516 | 0.00121929 | -2.26 | 2.40E-02 | 1.00E+00 |
| All | Phospholipids in Small HDL | 23574 | GRS (1 SD) | 0.00204107 | 0.00026943 | 7.58 | 3.61E-14 | 8.22E-12 |
| All | Phospholipids in Small HDL | 23574 | Adjusted GRS (1 SD) | 8.23E-05 | 0.00026755 | 0.31 | 7.58E-01 | 1.00E+00 |
| Statin users | Cholesterol in Small HDL | 23575 | Statin dose (1 SD) | -0.0017689 | 0.00072096 | -2.45 | 1.42E-02 | 1.00E+00 |
| Statin users | Cholesterol in Small HDL | 23575 | GRS (1 SD) | -0.0002975 | 0.00045629 | -0.65 | 5.14E-01 | 1.00E+00 |
| Statin users | Cholesterol in Small HDL | 23575 | Adjusted GRS (1 SD) | -0.0005954 | 0.00045039 | -1.32 | 1.86E-01 | 1.00E+00 |
| Non-statin users | Cholesterol in Small HDL | 23575 | GRS (1 SD) | -0.0008706 | 0.00020169 | -4.32 | 1.59E-05 | 3.62E-03 |
| Non-statin users | Cholesterol in Small HDL | 23575 | Adjusted GRS (1 SD) | -5.33E-05 | 0.00020029 | -0.27 | 7.90E-01 | 1.00E+00 |
| All | Cholesterol in Small HDL | 23575 | Statin dose (1 SD) | -0.0022968 | 0.00077693 | -2.96 | 3.12E-03 | 7.12E-01 |
| All | Cholesterol in Small HDL | 23575 | GRS (1 SD) | -0.0006984 | 0.00017732 | -3.94 | 8.19E-05 | 1.87E-02 |
| All | Cholesterol in Small HDL | 23575 | Adjusted GRS (1 SD) | -6.657E-05 | 0.00017605 | -0.38 | 7.05E-01 | 1.00E+00 |
| Statin users | CE in Small HDL | 23576 | Statin dose (1 SD) | -0.0014518 | 0.00055504 | -2.62 | 8.92E-03 | 1.00E+00 |
| Statin users | CE in Small HDL | 23576 | GRS (1 SD) | -0.0003516 | 0.00035123 | -1.00 | 3.17E-01 | 1.00E+00 |
| Statin users | CE in Small HDL | 23576 | Adjusted GRS (1 SD) | -0.0005302 | 0.00034668 | -1.53 | 1.26E-01 | 1.00E+00 |
| Non-statin users | CE in Small HDL | 23576 | GRS (1 SD) | -0.0006716 | 0.00015705 | -4.28 | 1.90E-05 | 4.34E-03 |
| Non-statin users | CE in Small HDL | 23576 | Adjusted GRS (1 SD) | -7.04E-05 | 0.00015596 | -0.45 | 6.52E-01 | 1.00E+00 |
| All | CE in Small HDL | 23576 | Statin dose (1 SD) | -0.0015335 | 0.00059918 | -2.56 | 1.05E-02 | 1.00E+00 |
| All | CE in Small HDL | 23576 | GRS (1 SD) | -0.0005833 | 0.00013764 | -4.24 | 2.26E-05 | 5.15E-03 |
| All | CE in Small HDL | 23576 | Adjusted GRS (1 SD) | -9.898E-05 | 0.00013666 | -0.72 | 4.69E-01 | 1.00E+00 |
| Statin users | FC in Small HDL | 23577 | Statin dose (1 SD) | -0.0003171 | 0.0001879 | -1.69 | 9.15E-02 | 1.00E+00 |
| Statin users | FC in Small HDL | 23577 | GRS (1 SD) | 5.4038E-05 | 0.000119 | 0.45 | 6.50E-01 | 1.00E+00 |
| Statin users | FC in Small HDL | 23577 | Adjusted GRS (1 SD) | -6.518E-05 | 0.00011746 | -0.55 | 5.79E-01 | 1.00E+00 |
| Non-statin users | FC in Small HDL | 23577 | GRS (1 SD) | -0.000199 | 5.2162E-05 | -3.81 | 1.37E-04 | 3.11E-02 |
| Non-statin users | FC in Small HDL | 23577 | Adjusted GRS (1 SD) | 1.7109E-05 | 5.1799E-05 | 0.33 | 7.41E-01 | 1.00E+00 |
| All | FC in Small HDL | 23577 | Statin dose (1 SD) | -0.0007633 | 0.00020524 | -3.72 | 2.01E-04 | 4.59E-02 |

| collective | metabolite | field | variable | Estimate | Std. Error | t value | p value | p value Bonferroni |
|---|---|---|---|---|---|---|---|---|
| All | FC in Small HDL | 23577 | GRS (1 SD) | -0.0001152 | 4.6267E-05 | -2.49 | 1.28E-02 | 1.00E+00 |
| All | FC in Small HDL | 23577 | Adjusted GRS (1 SD) | 3.2407E-05 | 4.5933E-05 | 0.71 | 4.80E-01 | 1.00E+00 |
| Statin users | TG in Small HDL | 23578 | Statin dose (1 SD) | 0.00028247 | 0.00019673 | 1.44 | 1.51E-01 | 1.00E+00 |
| Statin users | TG in Small HDL | 23578 | GRS (1 SD) | 0.0010957 | 0.0001238 | 8.85 | 9.47E-19 | 2.16E-16 |
| Statin users | TG in Small HDL | 23578 | Adjusted GRS (1 SD) | 0.00018587 | 0.00012245 | 1.52 | 1.29E-01 | 1.00E+00 |
| Non-statin users | TG in Small HDL | 23578 | GRS (1 SD) | 0.00062209 | 5.6402E-05 | 11.03 | 2.87E-28 | 6.54E-26 |
| Non-statin users | TG in Small HDL | 23578 | Adjusted GRS (1 SD) | 5.363E-05 | 5.6044E-05 | 0.96 | 3.39E-01 | 1.00E+00 |
| All | TG in Small HDL | 23578 | Statin dose (1 SD) | 1.2224E-05 | 0.00022013 | 0.06 | 9.56E-01 | 1.00E+00 |
| All | TG in Small HDL | 23578 | GRS (1 SD) | 0.00068225 | 5.0336E-05 | 13.55 | 8.09E-42 | 1.84E-39 |
| All | TG in Small HDL | 23578 | Adjusted GRS (1 SD) | 7.8157E-05 | 5.001E-05 | 1.56 | 1.18E-01 | 1.00E+00 |
| Statin users | Phospholipids to Total Lipids in Chylomicrons and XL VLDL [%] | 23579 | Statin dose (1 SD) | 0.24361102 | 0.04732164 | 5.15 | 2.70E-07 | 6.17E-05 |
| Statin users | Phospholipids to Total Lipids in Chylomicrons and XL VLDL [%] | 23579 | GRS (1 SD) | -0.1007092 | 0.03013922 | -3.34 | 8.35E-04 | 1.90E-01 |
| Statin users | Phospholipids to Total Lipids in Chylomicrons and XL VLDL [%] | 23579 | Adjusted GRS (1 SD) | -0.019731 | 0.02973941 | -0.66 | 5.07E-01 | 1.00E+00 |
| Non-statin users | Phospholipids to Total Lipids in Chylomicrons and XL VLDL [%] | 23579 | GRS (1 SD) | 0.08205577 | 0.01416793 | 5.79 | 6.99E-09 | 1.59E-06 |
| Non-statin users | Phospholipids to Total Lipids in Chylomicrons and XL VLDL [%] | 23579 | Adjusted GRS (1 SD) | -0.0149728 | 0.01407358 | -1.06 | 2.87E-01 | 1.00E+00 |
| All | Phospholipids to Total Lipids in Chylomicrons and XL VLDL [%] | 23579 | Statin dose (1 SD) | 0.34455679 | 0.04938284 | 6.98 | 3.23E-12 | 7.37E-10 |
| All | Phospholipids to Total Lipids in Chylomicrons and XL VLDL [%] | 23579 | GRS (1 SD) | 0.03576824 | 0.01214693 | 2.94 | 3.23E-03 | 7.37E-01 |
| All | Phospholipids to Total Lipids in Chylomicrons and XL VLDL [%] | 23579 | Adjusted GRS (1 SD) | -0.0173056 | 0.0120602 | -1.43 | 1.51E-01 | 1.00E+00 |
| Statin users | Cholesterol to Total Lipids in Chylomicrons and XL VLDL [%] | 23580 | Statin dose (1 SD) | 0.31704496 | 0.12962727 | 2.45 | 1.45E-02 | 1.00E+00 |

| collective | metabolite | field | variable | Estimate | Std. Error | t value | p value | p value Bonferroni |
|---|---|---|---|---|---|---|---|---|
| Statin users | Cholesterol to Total Lipids in Chylomicrons and XL VLDL [%] | 23580 | GRS (1 SD) | -0.8365194 | 0.08244314 | -10.15 | 3.96E-24 | 9.04E-22 |
| Statin users | Cholesterol to Total Lipids in Chylomicrons and XL VLDL [%] | 23580 | Adjusted GRS (1 SD) | -0.1326757 | 0.08154514 | -1.63 | 1.04E-01 | 1.00E+00 |
| Non-statin users | Cholesterol to Total Lipids in Chylomicrons and XL VLDL [%] | 23580 | GRS (1 SD) | -0.5404732 | 0.03864455 | -13.99 | 2.13E-44 | 4.86E-42 |
| Non-statin users | Cholesterol to Total Lipids in Chylomicrons and XL VLDL [%] | 23580 | Adjusted GRS (1 SD) | -0.034918 | 0.03842376 | -0.91 | 3.63E-01 | 1.00E+00 |
| All | Cholesterol to Total Lipids in Chylomicrons and XL VLDL [%] | 23580 | Statin dose (1 SD) | 0.34430924 | 0.13747236 | 2.50 | 1.23E-02 | 1.00E+00 |
| All | Cholesterol to Total Lipids in Chylomicrons and XL VLDL [%] | 23580 | GRS (1 SD) | -0.5578935 | 0.033171 | -16.82 | 2.13E-63 | 4.85E-61 |
| All | Cholesterol to Total Lipids in Chylomicrons and XL VLDL [%] | 23580 | Adjusted GRS (1 SD) | -0.0330561 | 0.03297407 | -1.00 | 3.16E-01 | 1.00E+00 |
| Statin users | CE to Total Lipids in Chylomicrons and XL VLDL [%] | 23581 | Statin dose (1 SD) | 0.09352698 | 0.08212643 | 1.14 | 2.55E-01 | 1.00E+00 |
| Statin users | CE to Total Lipids in Chylomicrons and XL VLDL [%] | 23581 | GRS (1 SD) | -0.4627891 | 0.0530243 | -8.73 | 2.81E-18 | 6.41E-16 |
| Statin users | CE to Total Lipids in Chylomicrons and XL VLDL [%] | 23581 | Adjusted GRS (1 SD) | -0.0518645 | 0.05241151 | -0.99 | 3.22E-01 | 1.00E+00 |
| Non-statin users | CE to Total Lipids in Chylomicrons and XL VLDL [%] | 23581 | GRS (1 SD) | -0.3982636 | 0.02774711 | -14.35 | 1.15E-46 | 2.62E-44 |
| Non-statin users | CE to Total Lipids in Chylomicrons and XL VLDL [%] | 23581 | Adjusted GRS (1 SD) | -0.0151721 | 0.02759035 | -0.55 | 5.82E-01 | 1.00E+00 |
| All | CE to Total Lipids in Chylomicrons and XL VLDL [%] | 23581 | Statin dose (1 SD) | 0.0243562 | 0.0891957 | 0.27 | 7.85E-01 | 1.00E+00 |
| All | CE to Total Lipids in Chylomicrons and XL VLDL [%] | 23581 | GRS (1 SD) | -0.3808276 | 0.02347381 | -16.22 | 4.01E-59 | 9.15E-57 |

| collective | metabolite | field | variable | Estimate | Std. Error | t value | p value | p value Bonferroni |
|---|---|---|---|---|---|---|---|---|
| All | CE to Total Lipids in Chylomicrons and XL VLDL [%] | 23581 | Adjusted GRS (1 SD) | -0.0080481 | 0.02333253 | -0.34 | 7.30E-01 | 1.00E+00 |
| Statin users | FC to Total Lipids in Chylomicrons and XL VLDL [%] | 23582 | Statin dose (1 SD) | 0.2235179 | 0.05836338 | 3.83 | 1.29E-04 | 2.95E-02 |
| Statin users | FC to Total Lipids in Chylomicrons and XL VLDL [%] | 23582 | GRS (1 SD) | -0.3737283 | 0.03716198 | -10.06 | 9.87E-24 | 2.25E-21 |
| Statin users | FC to Total Lipids in Chylomicrons and XL VLDL [%] | 23582 | Adjusted GRS (1 SD) | -0.080806 | 0.03675325 | -2.20 | 2.79E-02 | 1.00E+00 |
| Non-statin users | FC to Total Lipids in Chylomicrons and XL VLDL [%] | 23582 | GRS (1 SD) | -0.1422104 | 0.01423181 | -9.99 | 1.69E-23 | 3.86E-21 |
| Non-statin users | FC to Total Lipids in Chylomicrons and XL VLDL [%] | 23582 | Adjusted GRS (1 SD) | -0.0197475 | 0.01414247 | -1.40 | 1.63E-01 | 1.00E+00 |
| All | FC to Total Lipids in Chylomicrons and XL VLDL [%] | 23582 | Statin dose (1 SD) | 0.31995636 | 0.060154 | 5.32 | 1.07E-07 | 2.44E-05 |
| All | FC to Total Lipids in Chylomicrons and XL VLDL [%] | 23582 | GRS (1 SD) | -0.1770666 | 0.01268102 | -13.96 | 2.85E-44 | 6.49E-42 |
| All | FC to Total Lipids in Chylomicrons and XL VLDL [%] | 23582 | Adjusted GRS (1 SD) | -0.0250088 | 0.0126007 | -1.98 | 4.72E-02 | 1.00E+00 |
| Statin users | TG to Total Lipids in Chylomicrons and XL VLDL [%] | 23583 | Statin dose (1 SD) | -0.5606425 | 0.16058769 | -3.49 | 4.84E-04 | 1.10E-01 |
| Statin users | TG to Total Lipids in Chylomicrons and XL VLDL [%] | 23583 | GRS (1 SD) | 0.93722622 | 0.10159287 | 9.23 | 3.12E-20 | 7.12E-18 |
| Statin users | TG to Total Lipids in Chylomicrons and XL VLDL [%] | 23583 | Adjusted GRS (1 SD) | 0.15240425 | 0.10043923 | 1.52 | 1.29E-01 | 1.00E+00 |
| Non-statin users | TG to Total Lipids in Chylomicrons and XL VLDL [%] | 23583 | GRS (1 SD) | 0.45841822 | 0.04330548 | 10.59 | 3.60E-26 | 8.22E-24 |
| Non-statin users | TG to Total Lipids in Chylomicrons and XL VLDL [%] | 23583 | Adjusted GRS (1 SD) | 0.04989293 | 0.04303686 | 1.16 | 2.46E-01 | 1.00E+00 |

| collective | metabolite | field | variable | Estimate | Std. Error | t value | p value | p value Bonferroni |
|---|---|---|---|---|---|---|---|---|
| All | TG to Total Lipids in Chylomicrons and XL VLDL [%] | 23583 | Statin dose (1 SD) | -0.6888519 | 0.16756071 | -4.11 | 3.97E-05 | 9.06E-03 |
| All | TG to Total Lipids in Chylomicrons and XL VLDL [%] | 23583 | GRS (1 SD) | 0.52212569 | 0.03769463 | 13.85 | 1.35E-43 | 3.08E-41 |
| All | TG to Total Lipids in Chylomicrons and XL VLDL [%] | 23583 | Adjusted GRS (1 SD) | 0.05036308 | 0.03745571 | 1.34 | 1.79E-01 | 1.00E+00 |
| Statin users | Phospholipids to Total Lipids in Very Large VLDL [%] | 23584 | Statin dose (1 SD) | -0.111359 | 0.02984004 | -3.73 | 1.92E-04 | 4.37E-02 |
| Statin users | Phospholipids to Total Lipids in Very Large VLDL [%] | 23584 | GRS (1 SD) | 0.0787149 | 0.0191146 | 4.12 | 3.84E-05 | 8.75E-03 |
| Statin users | Phospholipids to Total Lipids in Very Large VLDL [%] | 23584 | Adjusted GRS (1 SD) | 0.02859339 | 0.01887317 | 1.52 | 1.30E-01 | 1.00E+00 |
| Non-statin users | Phospholipids to Total Lipids in Very Large VLDL [%] | 23584 | GRS (1 SD) | -0.0361345 | 0.00908556 | -3.98 | 6.98E-05 | 1.59E-02 |
| Non-statin users | Phospholipids to Total Lipids in Very Large VLDL [%] | 23584 | Adjusted GRS (1 SD) | 0.00072835 | 0.00901882 | 0.08 | 9.36E-01 | 1.00E+00 |
| All | Phospholipids to Total Lipids in Very Large VLDL [%] | 23584 | Statin dose (1 SD) | -0.23271 | 0.03164974 | -7.35 | 2.12E-13 | 4.82E-11 |
| All | Phospholipids to Total Lipids in Very Large VLDL [%] | 23584 | GRS (1 SD) | -0.0050239 | 0.00780649 | -0.64 | 5.20E-01 | 1.00E+00 |
| All | Phospholipids to Total Lipids in Very Large VLDL [%] | 23584 | Adjusted GRS (1 SD) | 0.00940702 | 0.0077473 | 1.21 | 2.25E-01 | 1.00E+00 |
| Statin users | Cholesterol to Total Lipids in Very Large VLDL [%] | 23585 | Statin dose (1 SD) | -0.1299414 | 0.07491552 | -1.73 | 8.29E-02 | 1.00E+00 |
| Statin users | Cholesterol to Total Lipids in Very Large VLDL [%] | 23585 | GRS (1 SD) | -0.5977085 | 0.04842169 | -12.34 | 7.15E-35 | 1.63E-32 |
| Statin users | Cholesterol to Total Lipids in Very Large VLDL [%] | 23585 | Adjusted GRS (1 SD) | -0.1031095 | 0.04797668 | -2.15 | 3.16E-02 | 1.00E+00 |

| collective | metabolite | field | variable | Estimate | Std. Error | t value | p value | p value Bonferroni |
|---|---|---|---|---|---|---|---|---|
| Non-statin users | Cholesterol to Total Lipids in Very Large VLDL [%] | 23585 | GRS (1 SD) | -0.5204837 | 0.02600227 | -20.02 | 6.22E-89 | 1.42E-86 |
| Non-statin users | Cholesterol to Total Lipids in Very Large VLDL [%] | 23585 | Adjusted GRS (1 SD) | -0.0348505 | 0.02586782 | -1.35 | 1.78E-01 | 1.00E+00 |
| All | Cholesterol to Total Lipids in Very Large VLDL [%] | 23585 | Statin dose (1 SD) | -0.3456552 | 0.08206367 | -4.21 | 2.56E-05 | 5.83E-03 |
| All | Cholesterol to Total Lipids in Very Large VLDL [%] | 23585 | GRS (1 SD) | -0.4767501 | 0.02235113 | -21.33 | 9.34E-101 | 2.13E-98 |
| All | Cholesterol to Total Lipids in Very Large VLDL [%] | 23585 | Adjusted GRS (1 SD) | -0.0266974 | 0.02222504 | -1.20 | 2.30E-01 | 1.00E+00 |
| Statin users | CE to Total Lipids in Very Large VLDL [%] | 23586 | Statin dose (1 SD) | -0.0814602 | 0.0628262 | -1.30 | 1.95E-01 | 1.00E+00 |
| Statin users | CE to Total Lipids in Very Large VLDL [%] | 23586 | GRS (1 SD) | -0.5202113 | 0.04073098 | -12.77 | 3.35E-37 | 7.63E-35 |
| Statin users | CE to Total Lipids in Very Large VLDL [%] | 23586 | Adjusted GRS (1 SD) | -0.0902428 | 0.04036755 | -2.24 | 2.54E-02 | 1.00E+00 |
| Non-statin users | CE to Total Lipids in Very Large VLDL [%] | 23586 | GRS (1 SD) | -0.4343635 | 0.02148849 | -20.21 | 1.20E-90 | 2.73E-88 |
| Non-statin users | CE to Total Lipids in Very Large VLDL [%] | 23586 | Adjusted GRS (1 SD) | -0.0260989 | 0.02137839 | -1.22 | 2.22E-01 | 1.00E+00 |
| All | CE to Total Lipids in Very Large VLDL [%] | 23586 | Statin dose (1 SD) | -0.2298855 | 0.06814218 | -3.37 | 7.45E-04 | 1.70E-01 |
| All | CE to Total Lipids in Very Large VLDL [%] | 23586 | GRS (1 SD) | -0.4048206 | 0.01842002 | -21.98 | 7.87E-107 | 1.79E-104 |
| All | CE to Total Lipids in Very Large VLDL [%] | 23586 | Adjusted GRS (1 SD) | -0.0217263 | 0.01831831 | -1.19 | 2.36E-01 | 1.00E+00 |
| Statin users | FC to Total Lipids in Very Large VLDL [%] | 23587 | Statin dose (1 SD) | -0.0484843 | 0.01649139 | -2.94 | 3.29E-03 | 7.51E-01 |
| Statin users | FC to Total Lipids in Very Large VLDL [%] | 23587 | GRS (1 SD) | -0.0774991 | 0.01071038 | -7.24 | 4.80E-13 | 1.09E-10 |
| Statin users | FC to Total Lipids in Very Large VLDL [%] | 23587 | Adjusted GRS (1 SD) | -0.0128691 | 0.01058514 | -1.22 | 2.24E-01 | 1.00E+00 |
| Non-statin users | FC to Total Lipids in Very Large VLDL [%] | 23587 | GRS (1 SD) | -0.0861226 | 0.00571357 | -15.07 | 2.81E-51 | 6.42E-49 |
| Non-statin users | FC to Total Lipids in Very Large VLDL [%] | 23587 | Adjusted GRS (1 SD) | -0.0087532 | 0.00567838 | -1.54 | 1.23E-01 | 1.00E+00 |
| All | FC to Total Lipids in Very Large VLDL [%] | 23587 | Statin dose (1 SD) | -0.1157701 | 0.01825686 | -6.34 | 2.39E-10 | 5.45E-08 |
| All | FC to Total Lipids in Very Large VLDL [%] | 23587 | GRS (1 SD) | -0.0719314 | 0.00494306 | -14.55 | 6.25E-48 | 1.43E-45 |
| All | FC to Total Lipids in Very Large VLDL [%] | 23587 | Adjusted GRS (1 SD) | -0.0049724 | 0.00491005 | -1.01 | 3.11E-01 | 1.00E+00 |
| Statin users | TG to Total Lipids in Very Large VLDL [%] | 23588 | Statin dose (1 SD) | 0.2413075 | 0.07672977 | 3.14 | 1.67E-03 | 3.80E-01 |

| collective | metabolite | field | variable | Estimate | Std. Error | t value | p value | p value Bonferroni |
|---|---|---|---|---|---|---|---|---|
| Statin users | TG to Total Lipids in Very Large VLDL [%] | 23588 | GRS (1 SD) | 0.51899191 | 0.0492004 | 10.55 | 6.08E-26 | 1.39E-23 |
| Statin users | TG to Total Lipids in Very Large VLDL [%] | 23588 | Adjusted GRS (1 SD) | 0.07451779 | 0.04869894 | 1.53 | 1.26E-01 | 1.00E+00 |
| Non-statin users | TG to Total Lipids in Very Large VLDL [%] | 23588 | GRS (1 SD) | 0.55662166 | 0.02627197 | 21.19 | 2.25E-99 | 5.12E-97 |
| Non-statin users | TG to Total Lipids in Very Large VLDL [%] | 23588 | Adjusted GRS (1 SD) | 0.03412493 | 0.02614332 | 1.31 | 1.92E-01 | 1.00E+00 |
| All | TG to Total Lipids in Very Large VLDL [%] | 23588 | Statin dose (1 SD) | 0.57837212 | 0.08648243 | 6.69 | 2.40E-11 | 5.48E-09 |
| All | TG to Total Lipids in Very Large VLDL [%] | 23588 | GRS (1 SD) | 0.48177632 | 0.02294337 | 21.00 | 1.03E-97 | 2.35E-95 |
| All | TG to Total Lipids in Very Large VLDL [%] | 23588 | Adjusted GRS (1 SD) | 0.01729252 | 0.02281265 | 0.76 | 4.48E-01 | 1.00E+00 |
| Statin users | Phospholipids to Total Lipids in Large VLDL [%] | 23589 | Statin dose (1 SD) | -0.0234403 | 0.04059591 | -0.58 | 5.64E-01 | 1.00E+00 |
| Statin users | Phospholipids to Total Lipids in Large VLDL [%] | 23589 | GRS (1 SD) | 0.11101882 | 0.02560726 | 4.34 | 1.46E-05 | 3.33E-03 |
| Statin users | Phospholipids to Total Lipids in Large VLDL [%] | 23589 | Adjusted GRS (1 SD) | 0.03643201 | 0.02528615 | 1.44 | 1.50E-01 | 1.00E+00 |
| Non-statin users | Phospholipids to Total Lipids in Large VLDL [%] | 23589 | GRS (1 SD) | 0.03602585 | 0.01168486 | 3.08 | 2.05E-03 | 4.67E-01 |
| Non-statin users | Phospholipids to Total Lipids in Large VLDL [%] | 23589 | Adjusted GRS (1 SD) | -0.0015466 | 0.01160364 | -0.13 | 8.94E-01 | 1.00E+00 |
| All | Phospholipids to Total Lipids in Large VLDL [%] | 23589 | Statin dose (1 SD) | -0.0865961 | 0.04219109 | -2.05 | 4.02E-02 | 1.00E+00 |
| All | Phospholipids to Total Lipids in Large VLDL [%] | 23589 | GRS (1 SD) | 0.05019456 | 0.01001516 | 5.01 | 5.40E-07 | 1.23E-04 |

| collective | metabolite | field | variable | Estimate | Std. Error | t value | p value | p value Bonferroni |
|---|---|---|---|---|---|---|---|---|
| All | Phospholipids to Total Lipids in Large VLDL [%] | 23589 | Adjusted GRS (1 SD) | 0.0060474 | 0.00994379 | 0.61 | 5.43E-01 | 1.00E+00 |
| Statin users | Cholesterol to Total Lipids in Large VLDL [%] | 23590 | Statin dose (1 SD) | -0.0272826 | 0.04944864 | -0.55 | 5.81E-01 | 1.00E+00 |
| Statin users | Cholesterol to Total Lipids in Large VLDL [%] | 23590 | GRS (1 SD) | -0.3226317 | 0.03112392 | -10.37 | 4.12E-25 | 9.39E-23 |
| Statin users | Cholesterol to Total Lipids in Large VLDL [%] | 23590 | Adjusted GRS (1 SD) | -0.0545737 | 0.03080301 | -1.77 | 7.65E-02 | 1.00E+00 |
| Non-statin users | Cholesterol to Total Lipids in Large VLDL [%] | 23590 | GRS (1 SD) | -0.2459778 | 0.01385866 | -17.75 | 2.33E-70 | 5.31E-68 |
| Non-statin users | Cholesterol to Total Lipids in Large VLDL [%] | 23590 | Adjusted GRS (1 SD) | -0.0157492 | 0.01378611 | -1.14 | 2.53E-01 | 1.00E+00 |
| All | Cholesterol to Total Lipids in Large VLDL [%] | 23590 | Statin dose (1 SD) | -0.2062998 | 0.05456371 | -3.78 | 1.57E-04 | 3.59E-02 |
| All | Cholesterol to Total Lipids in Large VLDL [%] | 23590 | GRS (1 SD) | -0.2251524 | 0.01243046 | -18.11 | 3.17E-73 | 7.23E-71 |
| All | Cholesterol to Total Lipids in Large VLDL [%] | 23590 | Adjusted GRS (1 SD) | -0.0098407 | 0.01235781 | -0.80 | 4.26E-01 | 1.00E+00 |
| Statin users | CE to Total Lipids in Large VLDL [%] | 23591 | Statin dose (1 SD) | -0.0388836 | 0.03761285 | -1.03 | 3.01E-01 | 1.00E+00 |
| Statin users | CE to Total Lipids in Large VLDL [%] | 23591 | GRS (1 SD) | -0.2761847 | 0.02362074 | -11.69 | 1.78E-31 | 4.05E-29 |
| Statin users | CE to Total Lipids in Large VLDL [%] | 23591 | Adjusted GRS (1 SD) | -0.0412821 | 0.02339478 | -1.76 | 7.76E-02 | 1.00E+00 |
| Non-statin users | CE to Total Lipids in Large VLDL [%] | 23591 | GRS (1 SD) | -0.2062725 | 0.01084058 | -19.03 | 1.46E-80 | 3.32E-78 |
| Non-statin users | CE to Total Lipids in Large VLDL [%] | 23591 | Adjusted GRS (1 SD) | -0.0123675 | 0.0107867 | -1.15 | 2.52E-01 | 1.00E+00 |
| All | CE to Total Lipids in Large VLDL [%] | 23591 | Statin dose (1 SD) | -0.1821509 | 0.04167855 | -4.37 | 1.25E-05 | 2.86E-03 |
| All | CE to Total Lipids in Large VLDL [%] | 23591 | GRS (1 SD) | -0.1894852 | 0.00977258 | -19.39 | 1.28E-83 | 2.92E-81 |
| All | CE to Total Lipids in Large VLDL [%] | 23591 | Adjusted GRS (1 SD) | -0.0070047 | 0.00971745 | -0.72 | 4.71E-01 | 1.00E+00 |
| Statin users | FC to Total Lipids in Large VLDL [%] | 23592 | Statin dose (1 SD) | 0.01159327 | 0.01654609 | 0.70 | 4.84E-01 | 1.00E+00 |
| Statin users | FC to Total Lipids in Large VLDL [%] | 23592 | GRS (1 SD) | -0.0464436 | 0.010517 | -4.42 | 1.01E-05 | 2.30E-03 |

| collective | metabolite | field | variable | Estimate | Std. Error | t value | p value | p value Bonferroni |
|---|---|---|---|---|---|---|---|---|
| Statin users | FC to Total Lipids in Large VLDL [%] | 23592 | Adjusted GRS (1 SD) | -0.0132871 | 0.01038543 | -1.28 | 2.01E-01 | 1.00E+00 |
| Non-statin users | FC to Total Lipids in Large VLDL [%] | 23592 | GRS (1 SD) | -0.0397052 | 0.00463167 | -8.57 | 1.03E-17 | 2.34E-15 |
| Non-statin users | FC to Total Lipids in Large VLDL [%] | 23592 | Adjusted GRS (1 SD) | -0.0033816 | 0.00460113 | -0.73 | 4.62E-01 | 1.00E+00 |
| All | FC to Total Lipids in Large VLDL [%] | 23592 | Statin dose (1 SD) | -0.0241554 | 0.01777455 | -1.36 | 1.74E-01 | 1.00E+00 |
| All | FC to Total Lipids in Large VLDL [%] | 23592 | GRS (1 SD) | -0.0356664 | 0.00402821 | -8.85 | 8.55E-19 | 1.95E-16 |
| All | FC to Total Lipids in Large VLDL [%] | 23592 | Adjusted GRS (1 SD) | -0.0028351 | 0.00400041 | -0.71 | 4.79E-01 | 1.00E+00 |
| Statin users | TG to Total Lipids in Large VLDL [%] | 23593 | Statin dose (1 SD) | 0.05073171 | 0.07128141 | 0.71 | 4.77E-01 | 1.00E+00 |
| Statin users | TG to Total Lipids in Large VLDL [%] | 23593 | GRS (1 SD) | 0.21161335 | 0.04489051 | 4.71 | 2.45E-06 | 5.58E-04 |
| Statin users | TG to Total Lipids in Large VLDL [%] | 23593 | Adjusted GRS (1 SD) | 0.01814332 | 0.0443337 | 0.41 | 6.82E-01 | 1.00E+00 |
| Non-statin users | TG to Total Lipids in Large VLDL [%] | 23593 | GRS (1 SD) | 0.20994868 | 0.01989381 | 10.55 | 5.07E-26 | 1.16E-23 |
| Non-statin users | TG to Total Lipids in Large VLDL [%] | 23593 | Adjusted GRS (1 SD) | 0.01729264 | 0.01976689 | 0.87 | 3.82E-01 | 1.00E+00 |
| All | TG to Total Lipids in Large VLDL [%] | 23593 | Statin dose (1 SD) | 0.29290057 | 0.07707977 | 3.80 | 1.46E-04 | 3.32E-02 |
| All | TG to Total Lipids in Large VLDL [%] | 23593 | GRS (1 SD) | 0.1749559 | 0.01747083 | 10.01 | 1.35E-23 | 3.08E-21 |
| All | TG to Total Lipids in Large VLDL [%] | 23593 | Adjusted GRS (1 SD) | 0.00379143 | 0.01735191 | 0.22 | 8.27E-01 | 1.00E+00 |
| Statin users | Phospholipids to Total Lipids in Medium VLDL [%] | 23594 | Statin dose (1 SD) | -0.1148336 | 0.02192928 | -5.24 | 1.68E-07 | 3.83E-05 |
| Statin users | Phospholipids to Total Lipids in Medium VLDL [%] | 23594 | GRS (1 SD) | -0.1612167 | 0.01381614 | -11.67 | 2.35E-31 | 5.35E-29 |
| Statin users | Phospholipids to Total Lipids in Medium VLDL [%] | 23594 | Adjusted GRS (1 SD) | -0.0090059 | 0.01368554 | -0.66 | 5.11E-01 | 1.00E+00 |
| Non-statin users | Phospholipids to Total Lipids in Medium VLDL [%] | 23594 | GRS (1 SD) | -0.1280962 | 0.00516107 | -24.82 | 1.61E-135 | 3.68E-133 |
| Non-statin users | Phospholipids to Total Lipids in Medium VLDL [%] | 23594 | Adjusted GRS (1 SD) | -0.0049267 | 0.00514266 | -0.96 | 3.38E-01 | 1.00E+00 |
| All | Phospholipids to Total Lipids in Medium VLDL [%] | 23594 | Statin dose (1 SD) | -0.2564926 | 0.02505095 | -10.24 | 1.81E-24 | 4.12E-22 |
| All | Phospholipids to Total Lipids in Medium VLDL [%] | 23594 | GRS (1 SD) | -0.1111074 | 0.00519295 | -21.40 | 2.29E-101 | 5.22E-99 |

| collective | metabolite | field | variable | Estimate | Std. Error | t value | p value | p value Bonferroni |
|---|---|---|---|---|---|---|---|---|
| All | Phospholipids to Total Lipids in Medium VLDL [%] | 23594 | Adjusted GRS (1 SD) | 0.00153738 | 0.00516539 | 0.30 | 7.66E-01 | 1.00E+00 |
| Statin users | Cholesterol to Total Lipids in Medium VLDL [%] | 23595 | Statin dose (1 SD) | -0.3306986 | 0.07805875 | -4.24 | 2.30E-05 | 5.24E-03 |
| Statin users | Cholesterol to Total Lipids in Medium VLDL [%] | 23595 | GRS (1 SD) | -0.681538 | 0.0493003 | -13.82 | 2.92E-43 | 6.66E-41 |
| Statin users | Cholesterol to Total Lipids in Medium VLDL [%] | 23595 | Adjusted GRS (1 SD) | -0.0814631 | 0.04890007 | -1.67 | 9.57E-02 | 1.00E+00 |
| Non-statin users | Cholesterol to Total Lipids in Medium VLDL [%] | 23595 | GRS (1 SD) | -0.4842221 | 0.01945264 | -24.89 | 2.68E-136 | 6.10E-134 |
| Non-statin users | Cholesterol to Total Lipids in Medium VLDL [%] | 23595 | Adjusted GRS (1 SD) | -0.0176976 | 0.01938364 | -0.91 | 3.61E-01 | 1.00E+00 |
| All | Cholesterol to Total Lipids in Medium VLDL [%] | 23595 | Statin dose (1 SD) | -0.7127646 | 0.08721578 | -8.17 | 3.43E-16 | 7.83E-14 |
| All | Cholesterol to Total Lipids in Medium VLDL [%] | 23595 | GRS (1 SD) | -0.4452897 | 0.01887081 | -23.60 | 8.07E-123 | 1.84E-120 |
| All | Cholesterol to Total Lipids in Medium VLDL [%] | 23595 | Adjusted GRS (1 SD) | -0.0055332 | 0.01877858 | -0.29 | 7.68E-01 | 1.00E+00 |
| Statin users | CE to Total Lipids in Medium VLDL [%] | 23596 | Statin dose (1 SD) | -0.237214 | 0.05852013 | -4.05 | 5.10E-05 | 1.16E-02 |
| Statin users | CE to Total Lipids in Medium VLDL [%] | 23596 | GRS (1 SD) | -0.5093273 | 0.03694682 | -13.79 | 4.98E-43 | 1.14E-40 |
| Statin users | CE to Total Lipids in Medium VLDL [%] | 23596 | Adjusted GRS (1 SD) | -0.0635426 | 0.03664566 | -1.73 | 8.29E-02 | 1.00E+00 |
| Non-statin users | CE to Total Lipids in Medium VLDL [%] | 23596 | GRS (1 SD) | -0.3597297 | 0.01467742 | -24.51 | 3.30E-132 | 7.51E-130 |
| Non-statin users | CE to Total Lipids in Medium VLDL [%] | 23596 | Adjusted GRS (1 SD) | -0.0135569 | 0.0146238 | -0.93 | 3.54E-01 | 1.00E+00 |
| All | CE to Total Lipids in Medium VLDL [%] | 23596 | Statin dose (1 SD) | -0.5024061 | 0.06493499 | -7.74 | 1.13E-14 | 2.57E-12 |
| All | CE to Total Lipids in Medium VLDL [%] | 23596 | GRS (1 SD) | -0.3327105 | 0.01412304 | -23.56 | 2.01E-122 | 4.58E-120 |
| All | CE to Total Lipids in Medium VLDL [%] | 23596 | Adjusted GRS (1 SD) | -0.0055163 | 0.0140539 | -0.39 | 6.95E-01 | 1.00E+00 |
| Statin users | FC to Total Lipids in Medium VLDL [%] | 23597 | Statin dose (1 SD) | -0.0934893 | 0.02043754 | -4.57 | 4.85E-06 | 1.11E-03 |
| Statin users | FC to Total Lipids in Medium VLDL [%] | 23597 | GRS (1 SD) | -0.172208 | 0.01289736 | -13.35 | 1.74E-40 | 3.96E-38 |

| collective | metabolite | field | variable | Estimate | Std. Error | t value | p value | p value Bonferroni |
|---|---|---|---|---|---|---|---|---|
| Statin users | FC to Total Lipids in Medium VLDL [%] | 23597 | Adjusted GRS (1 SD) | -0.0179177 | 0.01278873 | -1.40 | 1.61E-01 | 1.00E+00 |
| Non-statin users | FC to Total Lipids in Medium VLDL [%] | 23597 | GRS (1 SD) | -0.1244948 | 0.00499965 | -24.90 | 2.18E-136 | 4.97E-134 |
| Non-statin users | FC to Total Lipids in Medium VLDL [%] | 23597 | Adjusted GRS (1 SD) | -0.0041432 | 0.00498194 | -0.83 | 4.06E-01 | 1.00E+00 |
| All | FC to Total Lipids in Medium VLDL [%] | 23597 | Statin dose (1 SD) | -0.2103643 | 0.02318068 | -9.07 | 1.38E-19 | 3.14E-17 |
| All | FC to Total Lipids in Medium VLDL [%] | 23597 | GRS (1 SD) | -0.1125805 | 0.0049346 | -22.81 | 5.88E-115 | 1.34E-112 |
| All | FC to Total Lipids in Medium VLDL [%] | 23597 | Adjusted GRS (1 SD) | -1.853E-05 | 0.00490973 | 0.00 | 9.97E-01 | 1.00E+00 |
| Statin users | TG to Total Lipids in Medium VLDL [%] | 23598 | Statin dose (1 SD) | 0.44553472 | 0.09771456 | 4.56 | 5.21E-06 | 1.19E-03 |
| Statin users | TG to Total Lipids in Medium VLDL [%] | 23598 | GRS (1 SD) | 0.84275434 | 0.06174476 | 13.65 | 3.21E-42 | 7.32E-40 |
| Statin users | TG to Total Lipids in Medium VLDL [%] | 23598 | Adjusted GRS (1 SD) | 0.09046571 | 0.0612369 | 1.48 | 1.40E-01 | 1.00E+00 |
| Non-statin users | TG to Total Lipids in Medium VLDL [%] | 23598 | GRS (1 SD) | 0.61231741 | 0.02413399 | 25.37 | 1.68E-141 | 3.84E-139 |
| Non-statin users | TG to Total Lipids in Medium VLDL [%] | 23598 | Adjusted GRS (1 SD) | 0.02262361 | 0.02405165 | 0.94 | 3.47E-01 | 1.00E+00 |
| All | TG to Total Lipids in Medium VLDL [%] | 23598 | Statin dose (1 SD) | 0.96926016 | 0.1100495 | 8.81 | 1.52E-18 | 3.46E-16 |
| All | TG to Total Lipids in Medium VLDL [%] | 23598 | GRS (1 SD) | 0.55639697 | 0.02365236 | 23.52 | 4.46E-122 | 1.02E-119 |
| All | TG to Total Lipids in Medium VLDL [%] | 23598 | Adjusted GRS (1 SD) | 0.00399557 | 0.02353642 | 0.17 | 8.65E-01 | 1.00E+00 |
| Statin users | Phospholipids to Total Lipids in Small VLDL [%] | 23599 | Statin dose (1 SD) | -0.112206 | 0.02090741 | -5.37 | 8.26E-08 | 1.88E-05 |
| Statin users | Phospholipids to Total Lipids in Small VLDL [%] | 23599 | GRS (1 SD) | -0.1939358 | 0.01335131 | -14.53 | 1.48E-47 | 3.38E-45 |
| Statin users | Phospholipids to Total Lipids in Small VLDL [%] | 23599 | Adjusted GRS (1 SD) | -0.0249924 | 0.01324938 | -1.89 | 5.93E-02 | 1.00E+00 |
| Non-statin users | Phospholipids to Total Lipids in Small VLDL [%] | 23599 | GRS (1 SD) | -0.1801243 | 0.0063283 | -28.46 | 2.14E-177 | 4.88E-175 |
| Non-statin users | Phospholipids to Total Lipids in Small VLDL [%] | 23599 | Adjusted GRS (1 SD) | -0.0052936 | 0.00631264 | -0.84 | 4.02E-01 | 1.00E+00 |
| All | Phospholipids to Total Lipids in Small VLDL [%] | 23599 | Statin dose (1 SD) | -0.2383424 | 0.02437136 | -9.78 | 1.78E-22 | 4.06E-20 |
| All | Phospholipids to Total Lipids in Small VLDL [%] | 23599 | GRS (1 SD) | -0.1599165 | 0.00590713 | -27.07 | 6.66E-161 | 1.52E-158 |

| collective | metabolite | field | variable | Estimate | Std. Error | t value | p value | p value Bonferroni |
|---|---|---|---|---|---|---|---|---|
| All | Phospholipids to Total Lipids in Small VLDL [%] | 23599 | Adjusted GRS (1 SD) | -0.0016661 | 0.00588266 | -0.28 | 7.77E-01 | 1.00E+00 |
| Statin users | Cholesterol to Total Lipids in Small VLDL [%] | 23600 | Statin dose (1 SD) | -0.2140128 | 0.05066778 | -4.22 | 2.43E-05 | 5.54E-03 |
| Statin users | Cholesterol to Total Lipids in Small VLDL [%] | 23600 | GRS (1 SD) | -0.4434961 | 0.03208838 | -13.82 | 3.05E-43 | 6.96E-41 |
| Statin users | Cholesterol to Total Lipids in Small VLDL [%] | 23600 | Adjusted GRS (1 SD) | -0.0490324 | 0.03182814 | -1.54 | 1.23E-01 | 1.00E+00 |
| Non-statin users | Cholesterol to Total Lipids in Small VLDL [%] | 23600 | GRS (1 SD) | -0.3800496 | 0.01416478 | -26.83 | 6.18E-158 | 1.41E-155 |
| Non-statin users | Cholesterol to Total Lipids in Small VLDL [%] | 23600 | Adjusted GRS (1 SD) | -0.01333 | 0.01412252 | -0.94 | 3.45E-01 | 1.00E+00 |
| All | Cholesterol to Total Lipids in Small VLDL [%] | 23600 | Statin dose (1 SD) | -0.5238941 | 0.05903313 | -8.87 | 8.37E-19 | 1.91E-16 |
| All | Cholesterol to Total Lipids in Small VLDL [%] | 23600 | GRS (1 SD) | -0.3381424 | 0.01346611 | -25.11 | 8.90E-139 | 2.03E-136 |
| All | Cholesterol to Total Lipids in Small VLDL [%] | 23600 | Adjusted GRS (1 SD) | -0.0027596 | 0.0134045 | -0.21 | 8.37E-01 | 1.00E+00 |
| Statin users | CE to Total Lipids in Small VLDL [%] | 23601 | Statin dose (1 SD) | -0.0894685 | 0.03141224 | -2.85 | 4.41E-03 | 1.00E+00 |
| Statin users | CE to Total Lipids in Small VLDL [%] | 23601 | GRS (1 SD) | -0.2355324 | 0.0197919 | -11.90 | 1.53E-32 | 3.49E-30 |
| Statin users | CE to Total Lipids in Small VLDL [%] | 23601 | Adjusted GRS (1 SD) | -0.0231542 | 0.01960707 | -1.18 | 2.38E-01 | 1.00E+00 |
| Non-statin users | CE to Total Lipids in Small VLDL [%] | 23601 | GRS (1 SD) | -0.1930584 | 0.00858439 | -22.49 | 1.09E-111 | 2.48E-109 |
| Non-statin users | CE to Total Lipids in Small VLDL [%] | 23601 | Adjusted GRS (1 SD) | -0.0081926 | 0.00854844 | -0.96 | 3.38E-01 | 1.00E+00 |
| All | CE to Total Lipids in Small VLDL [%] | 23601 | Statin dose (1 SD) | -0.2636144 | 0.03601889 | -7.32 | 2.72E-13 | 6.20E-11 |
| All | CE to Total Lipids in Small VLDL [%] | 23601 | GRS (1 SD) | -0.1721091 | 0.00803402 | -21.42 | 1.29E-101 | 2.95E-99 |
| All | CE to Total Lipids in Small VLDL [%] | 23601 | Adjusted GRS (1 SD) | -0.0016619 | 0.00799143 | -0.21 | 8.35E-01 | 1.00E+00 |
| Statin users | FC to Total Lipids in Small VLDL [%] | 23602 | Statin dose (1 SD) | -0.1245467 | 0.02261094 | -5.51 | 3.75E-08 | 8.54E-06 |
| Statin users | FC to Total Lipids in Small VLDL [%] | 23602 | GRS (1 SD) | -0.2079619 | 0.01443378 | -14.41 | 8.05E-47 | 1.84E-44 |

| collective | metabolite | field | variable | Estimate | Std. Error | t value | p value | p value Bonferroni |
|---|---|---|---|---|---|---|---|---|
| Statin users | FC to Total Lipids in Small VLDL [%] | 23602 | Adjusted GRS (1 SD) | -0.025877 | 0.01432245 | -1.81 | 7.08E-02 | 1.00E+00 |
| Non-statin users | FC to Total Lipids in Small VLDL [%] | 23602 | GRS (1 SD) | -0.1869921 | 0.00669374 | -27.94 | 5.58E-171 | 1.27E-168 |
| Non-statin users | FC to Total Lipids in Small VLDL [%] | 23602 | Adjusted GRS (1 SD) | -0.0051364 | 0.00667606 | -0.77 | 4.42E-01 | 1.00E+00 |
| All | FC to Total Lipids in Small VLDL [%] | 23602 | Statin dose (1 SD) | -0.2602812 | 0.02627399 | -9.91 | 5.12E-23 | 1.17E-20 |
| All | FC to Total Lipids in Small VLDL [%] | 23602 | GRS (1 SD) | -0.1660337 | 0.00629922 | -26.36 | 1.17E-152 | 2.67E-150 |
| All | FC to Total Lipids in Small VLDL [%] | 23602 | Adjusted GRS (1 SD) | -0.0010965 | 0.0062721 | -0.17 | 8.61E-01 | 1.00E+00 |
| Statin users | TG to Total Lipids in Small VLDL [%] | 23603 | Statin dose (1 SD) | 0.32622518 | 0.07018508 | 4.65 | 3.41E-06 | 7.77E-04 |
| Statin users | TG to Total Lipids in Small VLDL [%] | 23603 | GRS (1 SD) | 0.63743504 | 0.04456183 | 14.30 | 3.53E-46 | 8.04E-44 |
| Statin users | TG to Total Lipids in Small VLDL [%] | 23603 | Adjusted GRS (1 SD) | 0.07402938 | 0.04421529 | 1.67 | 9.41E-02 | 1.00E+00 |
| Non-statin users | TG to Total Lipids in Small VLDL [%] | 23603 | GRS (1 SD) | 0.56017658 | 0.02008691 | 27.89 | 2.09E-170 | 4.77E-168 |
| Non-statin users | TG to Total Lipids in Small VLDL [%] | 23603 | Adjusted GRS (1 SD) | 0.01862472 | 0.02003352 | 0.93 | 3.53E-01 | 1.00E+00 |
| All | TG to Total Lipids in Small VLDL [%] | 23603 | Statin dose (1 SD) | 0.76224523 | 0.0820545 | 9.29 | 1.91E-20 | 4.36E-18 |
| All | TG to Total Lipids in Small VLDL [%] | 23603 | GRS (1 SD) | 0.49806102 | 0.01905 | 26.14 | 3.05E-150 | 6.95E-148 |
| All | TG to Total Lipids in Small VLDL [%] | 23603 | Adjusted GRS (1 SD) | 0.00442647 | 0.0189671 | 0.23 | 8.15E-01 | 1.00E+00 |
| Statin users | Phospholipids to Total Lipids in Very Small VLDL [%] | 23604 | Statin dose (1 SD) | 0.07009366 | 0.01125768 | 6.23 | 5.04E-10 | 1.15E-07 |
| Statin users | Phospholipids to Total Lipids in Very Small VLDL [%] | 23604 | GRS (1 SD) | 0.0401582 | 0.00717047 | 5.60 | 2.17E-08 | 4.94E-06 |
| Statin users | Phospholipids to Total Lipids in Very Small VLDL [%] | 23604 | Adjusted GRS (1 SD) | 0.00891458 | 0.00708337 | 1.26 | 2.08E-01 | 1.00E+00 |
| Non-statin users | Phospholipids to Total Lipids in Very Small VLDL [%] | 23604 | GRS (1 SD) | 0.03944969 | 0.00271071 | 14.55 | 6.33E-48 | 1.44E-45 |
| Non-statin users | Phospholipids to Total Lipids in Very Small VLDL [%] | 23604 | Adjusted GRS (1 SD) | 0.00325819 | 0.00269486 | 1.21 | 2.27E-01 | 1.00E+00 |
| All | Phospholipids to Total Lipids in Very Small VLDL [%] | 23604 | Statin dose (1 SD) | 0.0942542 | 0.01207272 | 7.81 | 6.50E-15 | 1.48E-12 |
| All | Phospholipids to Total Lipids in Very Small VLDL [%] | 23604 | GRS (1 SD) | 0.03409986 | 0.00251175 | 13.58 | 5.97E-42 | 1.36E-39 |

| collective | metabolite | field | variable | Estimate | Std. Error | t value | p value | p value Bonferroni |
|---|---|---|---|---|---|---|---|---|
| All | Phospholipids to Total Lipids in Very Small VLDL [%] | 23604 | Adjusted GRS (1 SD) | 0.00206402 | 0.0024955 | 0.83 | 4.08E-01 | 1.00E+00 |
| Statin users | Cholesterol to Total Lipids in Very Small VLDL [%] | 23605 | Statin dose (1 SD) | -0.4468279 | 0.05302035 | -8.43 | 4.21E-17 | 9.60E-15 |
| Statin users | Cholesterol to Total Lipids in Very Small VLDL [%] | 23605 | GRS (1 SD) | -0.4314691 | 0.03391414 | -12.72 | 6.24E-37 | 1.42E-34 |
| Statin users | Cholesterol to Total Lipids in Very Small VLDL [%] | 23605 | Adjusted GRS (1 SD) | -0.0439519 | 0.03361458 | -1.31 | 1.91E-01 | 1.00E+00 |
| Non-statin users | Cholesterol to Total Lipids in Very Small VLDL [%] | 23605 | GRS (1 SD) | -0.3334065 | 0.01275018 | -26.15 | 3.79E-150 | 8.65E-148 |
| Non-statin users | Cholesterol to Total Lipids in Very Small VLDL [%] | 23605 | Adjusted GRS (1 SD) | -0.0088668 | 0.01270958 | -0.70 | 4.85E-01 | 1.00E+00 |
| All | Cholesterol to Total Lipids in Very Small VLDL [%] | 23605 | Statin dose (1 SD) | -0.7419209 | 0.05991059 | -12.38 | 6.16E-35 | 1.40E-32 |
| All | Cholesterol to Total Lipids in Very Small VLDL [%] | 23605 | GRS (1 SD) | -0.2978825 | 0.01287894 | -23.13 | 4.32E-118 | 9.84E-116 |
| All | Cholesterol to Total Lipids in Very Small VLDL [%] | 23605 | Adjusted GRS (1 SD) | 0.00113836 | 0.0128148 | 0.09 | 9.29E-01 | 1.00E+00 |
| Statin users | CE to Total Lipids in Very Small VLDL [%] | 23606 | Statin dose (1 SD) | -0.4106892 | 0.04749607 | -8.65 | 6.43E-18 | 1.47E-15 |
| Statin users | CE to Total Lipids in Very Small VLDL [%] | 23606 | GRS (1 SD) | -0.3744271 | 0.03042563 | -12.31 | 1.13E-34 | 2.57E-32 |
| Statin users | CE to Total Lipids in Very Small VLDL [%] | 23606 | Adjusted GRS (1 SD) | -0.0384482 | 0.03014891 | -1.28 | 2.02E-01 | 1.00E+00 |
| Non-statin users | CE to Total Lipids in Very Small VLDL [%] | 23606 | GRS (1 SD) | -0.2991387 | 0.01149493 | -26.02 | 9.85E-149 | 2.25E-146 |
| Non-statin users | CE to Total Lipids in Very Small VLDL [%] | 23606 | Adjusted GRS (1 SD) | -0.0090349 | 0.01145789 | -0.79 | 4.30E-01 | 1.00E+00 |

| collective | metabolite | field | variable | Estimate | Std. Error | t value | p value | p value Bonferroni |
|---|---|---|---|---|---|---|---|---|
| All | CE to Total Lipids in Very Small VLDL [%] | 23606 | Statin dose (1 SD) | -0.6719586 | 0.05352321 | -12.55 | 7.50E-36 | 1.71E-33 |
| All | CE to Total Lipids in Very Small VLDL [%] | 23606 | GRS (1 SD) | -0.2652072 | 0.01156758 | -22.93 | 4.54E-116 | 1.03E-113 |
| All | CE to Total Lipids in Very Small VLDL [%] | 23606 | Adjusted GRS (1 SD) | 0.00031774 | 0.01150952 | 0.03 | 9.78E-01 | 1.00E+00 |
| Statin users | FC to Total Lipids in Very Small VLDL [%] | 23607 | Statin dose (1 SD) | -0.0361438 | 0.00774645 | -4.67 | 3.13E-06 | 7.13E-04 |
| Statin users | FC to Total Lipids in Very Small VLDL [%] | 23607 | GRS (1 SD) | -0.0570445 | 0.00486616 | -11.72 | 1.25E-31 | 2.84E-29 |
| Statin users | FC to Total Lipids in Very Small VLDL [%] | 23607 | Adjusted GRS (1 SD) | -0.005502 | 0.00482021 | -1.14 | 2.54E-01 | 1.00E+00 |
| Non-statin users | FC to Total Lipids in Very Small VLDL [%] | 23607 | GRS (1 SD) | -0.0342713 | 0.00178237 | -19.23 | 3.19E-82 | 7.27E-80 |
| Non-statin users | FC to Total Lipids in Very Small VLDL [%] | 23607 | Adjusted GRS (1 SD) | 0.0001648 | 0.00177355 | 0.09 | 9.26E-01 | 1.00E+00 |
| All | FC to Total Lipids in Very Small VLDL [%] | 23607 | Statin dose (1 SD) | -0.0699664 | 0.0085823 | -8.15 | 4.05E-16 | 9.23E-14 |
| All | FC to Total Lipids in Very Small VLDL [%] | 23607 | GRS (1 SD) | -0.032678 | 0.00174883 | -18.69 | 8.41E-78 | 1.92E-75 |
| All | FC to Total Lipids in Very Small VLDL [%] | 23607 | Adjusted GRS (1 SD) | 0.00081867 | 0.00173874 | 0.47 | 6.38E-01 | 1.00E+00 |
| Statin users | TG to Total Lipids in Very Small VLDL [%] | 23608 | Statin dose (1 SD) | 0.37673392 | 0.04762909 | 7.91 | 2.96E-15 | 6.74E-13 |
| Statin users | TG to Total Lipids in Very Small VLDL [%] | 23608 | GRS (1 SD) | 0.39131231 | 0.0303364 | 12.90 | 6.52E-38 | 1.49E-35 |
| Statin users | TG to Total Lipids in Very Small VLDL [%] | 23608 | Adjusted GRS (1 SD) | 0.03503584 | 0.0300722 | 1.17 | 2.44E-01 | 1.00E+00 |

| collective | metabolite | field | variable | Estimate | Std. Error | t value | p value | p value Bonferroni |
|---|---|---|---|---|---|---|---|---|
| Non-statin users | TG to Total Lipids in Very Small VLDL [%] | 23608 | GRS (1 SD) | 0.29395692 | 0.01131022 | 25.99 | 2.32E-148 | 5.29E-146 |
| Non-statin users | TG to Total Lipids in Very Small VLDL [%] | 23608 | Adjusted GRS (1 SD) | 0.0056078 | 0.01127369 | 0.50 | 6.19E-01 | 1.00E+00 |
| All | TG to Total Lipids in Very Small VLDL [%] | 23608 | Statin dose (1 SD) | 0.6476627 | 0.05393207 | 12.01 | 5.70E-33 | 1.30E-30 |
| All | TG to Total Lipids in Very Small VLDL [%] | 23608 | GRS (1 SD) | 0.26378326 | 0.01148167 | 22.97 | 1.53E-116 | 3.48E-114 |
| All | TG to Total Lipids in Very Small VLDL [%] | 23608 | Adjusted GRS (1 SD) | -0.003203 | 0.01142414 | -0.28 | 7.79E-01 | 1.00E+00 |
| Statin users | Phospholipids to Total Lipids in IDL [%] | 23609 | Statin dose (1 SD) | 0.06188088 | 0.01165153 | 5.31 | 1.12E-07 | 2.56E-05 |
| Statin users | Phospholipids to Total Lipids in IDL [%] | 23609 | GRS (1 SD) | 0.0437487 | 0.00749164 | 5.84 | 5.31E-09 | 1.21E-06 |
| Statin users | Phospholipids to Total Lipids in IDL [%] | 23609 | Adjusted GRS (1 SD) | 0.00572274 | 0.00740135 | 0.77 | 4.39E-01 | 1.00E+00 |
| Non-statin users | Phospholipids to Total Lipids in IDL [%] | 23609 | GRS (1 SD) | 0.05713119 | 0.00295337 | 19.34 | 3.39E-83 | 7.72E-81 |
| Non-statin users | Phospholipids to Total Lipids in IDL [%] | 23609 | Adjusted GRS (1 SD) | 0.00299999 | 0.00293881 | 1.02 | 3.07E-01 | 1.00E+00 |
| All | Phospholipids to Total Lipids in IDL [%] | 23609 | Statin dose (1 SD) | 0.08792594 | 0.01250889 | 7.03 | 2.23E-12 | 5.09E-10 |
| All | Phospholipids to Total Lipids in IDL [%] | 23609 | GRS (1 SD) | 0.05045246 | 0.00267472 | 18.86 | 3.03E-79 | 6.91E-77 |
| All | Phospholipids to Total Lipids in IDL [%] | 23609 | Adjusted GRS (1 SD) | 0.0015573 | 0.00265936 | 0.59 | 5.58E-01 | 1.00E+00 |
| Statin users | Cholesterol to Total Lipids in IDL [%] | 23610 | Statin dose (1 SD) | -0.3612806 | 0.03932871 | -9.19 | 5.21E-20 | 1.19E-17 |
| Statin users | Cholesterol to Total Lipids in IDL [%] | 23610 | GRS (1 SD) | -0.3069977 | 0.02497732 | -12.29 | 1.36E-34 | 3.10E-32 |
| Statin users | Cholesterol to Total Lipids in IDL [%] | 23610 | Adjusted GRS (1 SD) | -0.0261405 | 0.02475024 | -1.06 | 2.91E-01 | 1.00E+00 |
| Non-statin users | Cholesterol to Total Lipids in IDL [%] | 23610 | GRS (1 SD) | -0.2342087 | 0.0085628 | -27.35 | 5.01E-164 | 1.14E-161 |
| Non-statin users | Cholesterol to Total Lipids in IDL [%] | 23610 | Adjusted GRS (1 SD) | -0.0098335 | 0.0085386 | -1.15 | 2.49E-01 | 1.00E+00 |
| All | Cholesterol to Total Lipids in IDL [%] | 23610 | Statin dose (1 SD) | -0.5209174 | 0.04295811 | -12.13 | 1.40E-33 | 3.20E-31 |
| All | Cholesterol to Total Lipids in IDL [%] | 23610 | GRS (1 SD) | -0.21886 | 0.00857453 | -25.52 | 2.61E-143 | 5.96E-141 |
| All | Cholesterol to Total Lipids in IDL [%] | 23610 | Adjusted GRS (1 SD) | -0.0035292 | 0.00853605 | -0.41 | 6.79E-01 | 1.00E+00 |
| Statin users | CE to Total Lipids in IDL [%] | 23611 | Statin dose (1 SD) | -0.3073834 | 0.0314614 | -9.77 | 2.07E-22 | 4.72E-20 |
| Statin users | CE to Total Lipids in IDL [%] | 23611 | GRS (1 SD) | -0.2166119 | 0.02001227 | -10.82 | 3.17E-27 | 7.23E-25 |

| collective | metabolite | field | variable | Estimate | Std. Error | t value | p value | p value Bonferroni |
|---|---|---|---|---|---|---|---|---|
| Statin users | CE to Total Lipids in IDL [%] | 23611 | Adjusted GRS (1 SD) | -0.0106153 | 0.01981352 | -0.54 | 5.92E-01 | 1.00E+00 |
| Non-statin users | CE to Total Lipids in IDL [%] | 23611 | GRS (1 SD) | -0.2000028 | 0.00724027 | -27.62 | 3.01E-167 | 6.87E-165 |
| Non-statin users | CE to Total Lipids in IDL [%] | 23611 | Adjusted GRS (1 SD) | -0.006924 | 0.00722043 | -0.96 | 3.38E-01 | 1.00E+00 |
| All | CE to Total Lipids in IDL [%] | 23611 | Statin dose (1 SD) | -0.4362566 | 0.03432386 | -12.71 | 1.08E-36 | 2.45E-34 |
| All | CE to Total Lipids in IDL [%] | 23611 | GRS (1 SD) | -0.1813387 | 0.00705056 | -25.72 | 1.79E-145 | 4.08E-143 |
| All | CE to Total Lipids in IDL [%] | 23611 | Adjusted GRS (1 SD) | -0.0002964 | 0.00701922 | -0.04 | 9.66E-01 | 1.00E+00 |
| Statin users | FC to Total Lipids in IDL [%] | 23612 | Statin dose (1 SD) | -0.0538892 | 0.01503398 | -3.58 | 3.40E-04 | 7.75E-02 |
| Statin users | FC to Total Lipids in IDL [%] | 23612 | GRS (1 SD) | -0.0903877 | 0.0095163 | -9.50 | 2.38E-21 | 5.42E-19 |
| Statin users | FC to Total Lipids in IDL [%] | 23612 | Adjusted GRS (1 SD) | -0.0155286 | 0.00941468 | -1.65 | 9.91E-02 | 1.00E+00 |
| Non-statin users | FC to Total Lipids in IDL [%] | 23612 | GRS (1 SD) | -0.0342056 | 0.00326084 | -10.49 | 9.96E-26 | 2.27E-23 |
| Non-statin users | FC to Total Lipids in IDL [%] | 23612 | Adjusted GRS (1 SD) | -0.0029076 | 0.00323991 | -0.90 | 3.69E-01 | 1.00E+00 |
| All | FC to Total Lipids in IDL [%] | 23612 | Statin dose (1 SD) | -0.0846533 | 0.0161104 | -5.25 | 1.52E-07 | 3.46E-05 |
| All | FC to Total Lipids in IDL [%] | 23612 | GRS (1 SD) | -0.0375216 | 0.00313369 | -11.97 | 5.11E-33 | 1.16E-30 |
| All | FC to Total Lipids in IDL [%] | 23612 | Adjusted GRS (1 SD) | -0.0032323 | 0.00311288 | -1.04 | 2.99E-01 | 1.00E+00 |
| Statin users | TG to Total Lipids in IDL [%] | 23613 | Statin dose (1 SD) | 0.29939453 | 0.03286604 | 9.11 | 1.05E-19 | 2.39E-17 |
| Statin users | TG to Total Lipids in IDL [%] | 23613 | GRS (1 SD) | 0.26325197 | 0.02087033 | 12.61 | 2.47E-36 | 5.62E-34 |
| Statin users | TG to Total Lipids in IDL [%] | 23613 | Adjusted GRS (1 SD) | 0.0204234 | 0.02068492 | 0.99 | 3.23E-01 | 1.00E+00 |
| Non-statin users | TG to Total Lipids in IDL [%] | 23613 | GRS (1 SD) | 0.17707697 | 0.00685294 | 25.84 | 1.13E-146 | 2.57E-144 |
| Non-statin users | TG to Total Lipids in IDL [%] | 23613 | Adjusted GRS (1 SD) | 0.00683302 | 0.00683048 | 1.00 | 3.17E-01 | 1.00E+00 |
| All | TG to Total Lipids in IDL [%] | 23613 | Statin dose (1 SD) | 0.43298895 | 0.03615458 | 11.98 | 8.42E-33 | 1.92E-30 |
| All | TG to Total Lipids in IDL [%] | 23613 | GRS (1 SD) | 0.16840788 | 0.00703994 | 23.92 | 3.66E-126 | 8.34E-124 |
| All | TG to Total Lipids in IDL [%] | 23613 | Adjusted GRS (1 SD) | 0.00197256 | 0.00700599 | 0.28 | 7.78E-01 | 1.00E+00 |
| Statin users | Phospholipids to Total Lipids in Large LDL [%] | 23614 | Statin dose (1 SD) | -0.0285354 | 0.01062105 | -2.69 | 7.23E-03 | 1.00E+00 |
| Statin users | Phospholipids to Total Lipids in Large LDL [%] | 23614 | GRS (1 SD) | -0.0149775 | 0.00711504 | -2.11 | 3.53E-02 | 1.00E+00 |
| Statin users | Phospholipids to Total Lipids in Large LDL [%] | 23614 | Adjusted GRS (1 SD) | -0.0103514 | 0.00702363 | -1.47 | 1.41E-01 | 1.00E+00 |

| collective | metabolite | field | variable | Estimate | Std. Error | t value | p value | p value Bonferroni |
|---|---|---|---|---|---|---|---|---|
| Non-statin users | Phospholipids to Total Lipids in Large LDL [%] | 23614 | GRS (1 SD) | -0.0002503 | 0.00253119 | -0.10 | 9.21E-01 | 1.00E+00 |
| Non-statin users | Phospholipids to Total Lipids in Large LDL [%] | 23614 | Adjusted GRS (1 SD) | 0.00037806 | 0.00251339 | 0.15 | 8.80E-01 | 1.00E+00 |
| All | Phospholipids to Total Lipids in Large LDL [%] | 23614 | Statin dose (1 SD) | -0.0236587 | 0.01139693 | -2.08 | 3.79E-02 | 1.00E+00 |
| All | Phospholipids to Total Lipids in Large LDL [%] | 23614 | GRS (1 SD) | -0.0014637 | 0.00232995 | -0.63 | 5.30E-01 | 1.00E+00 |
| All | Phospholipids to Total Lipids in Large LDL [%] | 23614 | Adjusted GRS (1 SD) | -0.0011902 | 0.00231307 | -0.51 | 6.07E-01 | 1.00E+00 |
| Statin users | Cholesterol to Total Lipids in Large LDL [%] | 23615 | Statin dose (1 SD) | -0.153947 | 0.02643774 | -5.82 | 6.02E-09 | 1.37E-06 |
| Statin users | Cholesterol to Total Lipids in Large LDL [%] | 23615 | GRS (1 SD) | -0.1565408 | 0.01811474 | -8.64 | 5.97E-18 | 1.36E-15 |
| Statin users | Cholesterol to Total Lipids in Large LDL [%] | 23615 | Adjusted GRS (1 SD) | 0.00249883 | 0.0179154 | 0.14 | 8.89E-01 | 1.00E+00 |
| Non-statin users | Cholesterol to Total Lipids in Large LDL [%] | 23615 | GRS (1 SD) | -0.1183026 | 0.00539529 | -21.93 | 2.77E-106 | 6.31E-104 |
| Non-statin users | Cholesterol to Total Lipids in Large LDL [%] | 23615 | Adjusted GRS (1 SD) | -0.0072398 | 0.00537191 | -1.35 | 1.78E-01 | 1.00E+00 |
| All | Cholesterol to Total Lipids in Large LDL [%] | 23615 | Statin dose (1 SD) | -0.2398706 | 0.02953739 | -8.12 | 5.24E-16 | 1.19E-13 |
| All | Cholesterol to Total Lipids in Large LDL [%] | 23615 | GRS (1 SD) | -0.1110813 | 0.00553956 | -20.05 | 2.72E-89 | 6.20E-87 |
| All | Cholesterol to Total Lipids in Large LDL [%] | 23615 | Adjusted GRS (1 SD) | -0.0009035 | 0.00550885 | -0.16 | 8.70E-01 | 1.00E+00 |
| Statin users | CE to Total Lipids in Large LDL [%] | 23616 | Statin dose (1 SD) | -0.064081 | 0.01887011 | -3.40 | 6.88E-04 | 1.57E-01 |
| Statin users | CE to Total Lipids in Large LDL [%] | 23616 | GRS (1 SD) | -0.0185702 | 0.01292798 | -1.44 | 1.51E-01 | 1.00E+00 |

| collective | metabolite | field | variable | Estimate | Std. Error | t value | p value | p value Bonferroni |
|---|---|---|---|---|---|---|---|---|
| Statin users | CE to Total Lipids in Large LDL [%] | 23616 | Adjusted GRS (1 SD) | 0.01670152 | 0.01276125 | 1.31 | 1.91E-01 | 1.00E+00 |
| Non-statin users | CE to Total Lipids in Large LDL [%] | 23616 | GRS (1 SD) | -0.0317808 | 0.00406383 | -7.82 | 5.32E-15 | 1.21E-12 |
| Non-statin users | CE to Total Lipids in Large LDL [%] | 23616 | Adjusted GRS (1 SD) | -0.0027405 | 0.00403664 | -0.68 | 4.97E-01 | 1.00E+00 |
| All | CE to Total Lipids in Large LDL [%] | 23616 | Statin dose (1 SD) | -0.0935194 | 0.02066086 | -4.53 | 6.08E-06 | 1.39E-03 |
| All | CE to Total Lipids in Large LDL [%] | 23616 | GRS (1 SD) | -0.0269442 | 0.003918 | -6.88 | 6.14E-12 | 1.40E-09 |
| All | CE to Total Lipids in Large LDL [%] | 23616 | Adjusted GRS (1 SD) | 0.00185866 | 0.00389039 | 0.48 | 6.33E-01 | 1.00E+00 |
| Statin users | FC to Total Lipids in Large LDL [%] | 23617 | Statin dose (1 SD) | -0.0898702 | 0.01742447 | -5.16 | 2.57E-07 | 5.85E-05 |
| Statin users | FC to Total Lipids in Large LDL [%] | 23617 | GRS (1 SD) | -0.1379669 | 0.01111578 | -12.41 | 3.07E-35 | 7.00E-33 |
| Statin users | FC to Total Lipids in Large LDL [%] | 23617 | Adjusted GRS (1 SD) | -0.014201 | 0.01101541 | -1.29 | 1.97E-01 | 1.00E+00 |
| Non-statin users | FC to Total Lipids in Large LDL [%] | 23617 | GRS (1 SD) | -0.0865225 | 0.00380973 | -22.71 | 7.44E-114 | 1.70E-111 |
| Non-statin users | FC to Total Lipids in Large LDL [%] | 23617 | Adjusted GRS (1 SD) | -0.004498 | 0.00379398 | -1.19 | 2.36E-01 | 1.00E+00 |
| All | FC to Total Lipids in Large LDL [%] | 23617 | Statin dose (1 SD) | -0.146358 | 0.01900909 | -7.70 | 1.51E-14 | 3.45E-12 |
| All | FC to Total Lipids in Large LDL [%] | 23617 | GRS (1 SD) | -0.0841374 | 0.00378126 | -22.25 | 1.86E-109 | 4.24E-107 |
| All | FC to Total Lipids in Large LDL [%] | 23617 | Adjusted GRS (1 SD) | -0.0027613 | 0.00376179 | -0.73 | 4.63E-01 | 1.00E+00 |
| Statin users | TG to Total Lipids in Large LDL [%] | 23618 | Statin dose (1 SD) | 0.18248651 | 0.02492264 | 7.32 | 2.70E-13 | 6.16E-11 |
| Statin users | TG to Total Lipids in Large LDL [%] | 23618 | GRS (1 SD) | 0.17151182 | 0.0164778 | 10.41 | 2.64E-25 | 6.02E-23 |
| Statin users | TG to Total Lipids in Large LDL [%] | 23618 | Adjusted GRS (1 SD) | 0.0078488 | 0.01631048 | 0.48 | 6.30E-01 | 1.00E+00 |
| Non-statin users | TG to Total Lipids in Large LDL [%] | 23618 | GRS (1 SD) | 0.1185547 | 0.00522558 | 22.69 | 1.27E-113 | 2.89E-111 |
| Non-statin users | TG to Total Lipids in Large LDL [%] | 23618 | Adjusted GRS (1 SD) | 0.00686293 | 0.00520393 | 1.32 | 1.87E-01 | 1.00E+00 |
| All | TG to Total Lipids in Large LDL [%] | 23618 | Statin dose (1 SD) | 0.26353482 | 0.02784653 | 9.46 | 3.72E-21 | 8.49E-19 |
| All | TG to Total Lipids in Large LDL [%] | 23618 | GRS (1 SD) | 0.11254562 | 0.00532723 | 21.13 | 6.95E-99 | 1.59E-96 |
| All | TG to Total Lipids in Large LDL [%] | 23618 | Adjusted GRS (1 SD) | 0.00209437 | 0.0052987 | 0.40 | 6.93E-01 | 1.00E+00 |
| Statin users | Phospholipids to Total Lipids in Medium LDL [%] | 23619 | Statin dose (1 SD) | 0.03413405 | 0.0102218 | 3.34 | 8.44E-04 | 1.92E-01 |
| Statin users | Phospholipids to Total Lipids in Medium LDL [%] | 23619 | GRS (1 SD) | -0.0317615 | 0.00652068 | -4.87 | 1.12E-06 | 2.55E-04 |
| Statin users | Phospholipids to Total Lipids in Medium LDL [%] | 23619 | Adjusted GRS (1 SD) | -0.0057267 | 0.00644033 | -0.89 | 3.74E-01 | 1.00E+00 |

| collective | metabolite | field | variable | Estimate | Std. Error | t value | p value | p value Bonferroni |
|---|---|---|---|---|---|---|---|---|
| Non-statin users | Phospholipids to Total Lipids in Medium LDL [%] | 23619 | GRS (1 SD) | -0.0479861 | 0.00273636 | -17.54 | 9.88E-69 | 2.25E-66 |
| Non-statin users | Phospholipids to Total Lipids in Medium LDL [%] | 23619 | Adjusted GRS (1 SD) | -0.0026703 | 0.00272184 | -0.98 | 3.27E-01 | 1.00E+00 |
| All | Phospholipids to Total Lipids in Medium LDL [%] | 23619 | Statin dose (1 SD) | 0.05165316 | 0.01085029 | 4.76 | 1.96E-06 | 4.47E-04 |
| All | Phospholipids to Total Lipids in Medium LDL [%] | 23619 | GRS (1 SD) | -0.0471788 | 0.00242875 | -19.43 | 6.42E-84 | 1.46E-81 |
| All | Phospholipids to Total Lipids in Medium LDL [%] | 23619 | Adjusted GRS (1 SD) | -0.0039413 | 0.002415 | -1.63 | 1.03E-01 | 1.00E+00 |
| Statin users | Cholesterol to Total Lipids in Medium LDL [%] | 23620 | Statin dose (1 SD) | -0.1783527 | 0.02587153 | -6.89 | 5.88E-12 | 1.34E-09 |
| Statin users | Cholesterol to Total Lipids in Medium LDL [%] | 23620 | GRS (1 SD) | -0.1315012 | 0.01634069 | -8.05 | 8.94E-16 | 2.04E-13 |
| Statin users | Cholesterol to Total Lipids in Medium LDL [%] | 23620 | Adjusted GRS (1 SD) | -0.0049028 | 0.01615672 | -0.30 | 7.62E-01 | 1.00E+00 |
| Non-statin users | Cholesterol to Total Lipids in Medium LDL [%] | 23620 | GRS (1 SD) | -0.0700603 | 0.00503409 | -13.92 | 5.55E-44 | 1.27E-41 |
| Non-statin users | Cholesterol to Total Lipids in Medium LDL [%] | 23620 | Adjusted GRS (1 SD) | -0.0038713 | 0.00500417 | -0.77 | 4.39E-01 | 1.00E+00 |
| All | Cholesterol to Total Lipids in Medium LDL [%] | 23620 | Statin dose (1 SD) | -0.2677527 | 0.02755682 | -9.72 | 3.30E-22 | 7.53E-20 |
| All | Cholesterol to Total Lipids in Medium LDL [%] | 23620 | GRS (1 SD) | -0.0647541 | 0.00514 | -12.60 | 2.28E-36 | 5.21E-34 |
| All | Cholesterol to Total Lipids in Medium LDL [%] | 23620 | Adjusted GRS (1 SD) | 0.00118357 | 0.00510621 | 0.23 | 8.17E-01 | 1.00E+00 |
| Statin users | CE to Total Lipids in Medium LDL [%] | 23621 | Statin dose (1 SD) | -0.1324105 | 0.02502437 | -5.29 | 1.25E-07 | 2.85E-05 |
| Statin users | CE to Total Lipids in Medium LDL [%] | 23621 | GRS (1 SD) | 0.07624865 | 0.01555838 | 4.90 | 9.62E-07 | 2.19E-04 |

| collective | metabolite | field | variable | Estimate | Std. Error | t value | p value | p value Bonferroni |
|---|---|---|---|---|---|---|---|---|
| Statin users | CE to Total Lipids in Medium LDL [%] | 23621 | Adjusted GRS (1 SD) | 0.01739986 | 0.01536661 | 1.13 | 2.58E-01 | 1.00E+00 |
| Non-statin users | CE to Total Lipids in Medium LDL [%] | 23621 | GRS (1 SD) | 0.06117854 | 0.00635235 | 9.63 | 6.07E-22 | 1.38E-19 |
| Non-statin users | CE to Total Lipids in Medium LDL [%] | 23621 | Adjusted GRS (1 SD) | 0.00377276 | 0.00631098 | 0.60 | 5.50E-01 | 1.00E+00 |
| All | CE to Total Lipids in Medium LDL [%] | 23621 | Statin dose (1 SD) | -0.2042529 | 0.02649796 | -7.71 | 1.41E-14 | 3.22E-12 |
| All | CE to Total Lipids in Medium LDL [%] | 23621 | GRS (1 SD) | 0.07182374 | 0.00570094 | 12.60 | 2.27E-36 | 5.18E-34 |
| All | CE to Total Lipids in Medium LDL [%] | 23621 | Adjusted GRS (1 SD) | 0.00904604 | 0.0056634 | 1.60 | 1.10E-01 | 1.00E+00 |
| Statin users | FC to Total Lipids in Medium LDL [%] | 23622 | Statin dose (1 SD) | -0.0459398 | 0.0250201 | -1.84 | 6.64E-02 | 1.00E+00 |
| Statin users | FC to Total Lipids in Medium LDL [%] | 23622 | GRS (1 SD) | -0.2077462 | 0.01575602 | -13.19 | 1.58E-39 | 3.60E-37 |
| Statin users | FC to Total Lipids in Medium LDL [%] | 23622 | Adjusted GRS (1 SD) | -0.0223009 | 0.01562151 | -1.43 | 1.53E-01 | 1.00E+00 |
| Non-statin users | FC to Total Lipids in Medium LDL [%] | 23622 | GRS (1 SD) | -0.13124 | 0.00589944 | -22.25 | 2.47E-109 | 5.62E-107 |
| Non-statin users | FC to Total Lipids in Medium LDL [%] | 23622 | Adjusted GRS (1 SD) | -0.0076442 | 0.00587435 | -1.30 | 1.93E-01 | 1.00E+00 |
| All | FC to Total Lipids in Medium LDL [%] | 23622 | Statin dose (1 SD) | -0.0634981 | 0.02684468 | -2.37 | 1.80E-02 | 1.00E+00 |
| All | FC to Total Lipids in Medium LDL [%] | 23622 | GRS (1 SD) | -0.1365775 | 0.00547831 | -24.93 | 7.91E-137 | 1.80E-134 |
| All | FC to Total Lipids in Medium LDL [%] | 23622 | Adjusted GRS (1 SD) | -0.0078614 | 0.00545298 | -1.44 | 1.49E-01 | 1.00E+00 |
| Statin users | TG to Total Lipids in Medium LDL [%] | 23623 | Statin dose (1 SD) | 0.14421545 | 0.02257378 | 6.39 | 1.78E-10 | 4.05E-08 |
| Statin users | TG to Total Lipids in Medium LDL [%] | 23623 | GRS (1 SD) | 0.16325685 | 0.01406367 | 11.61 | 4.73E-31 | 1.08E-28 |
| Statin users | TG to Total Lipids in Medium LDL [%] | 23623 | Adjusted GRS (1 SD) | 0.01062488 | 0.01393018 | 0.76 | 4.46E-01 | 1.00E+00 |
| Non-statin users | TG to Total Lipids in Medium LDL [%] | 23623 | GRS (1 SD) | 0.11804893 | 0.00475456 | 24.83 | 1.30E-135 | 2.96E-133 |
| Non-statin users | TG to Total Lipids in Medium LDL [%] | 23623 | Adjusted GRS (1 SD) | 0.00654366 | 0.00473759 | 1.38 | 1.67E-01 | 1.00E+00 |
| All | TG to Total Lipids in Medium LDL [%] | 23623 | Statin dose (1 SD) | 0.21609722 | 0.02443001 | 8.85 | 1.08E-18 | 2.47E-16 |
| All | TG to Total Lipids in Medium LDL [%] | 23623 | GRS (1 SD) | 0.11193346 | 0.00470587 | 23.79 | 9.25E-125 | 2.11E-122 |
| All | TG to Total Lipids in Medium LDL [%] | 23623 | Adjusted GRS (1 SD) | 0.00275784 | 0.00468304 | 0.59 | 5.56E-01 | 1.00E+00 |
| Statin users | Phospholipids to Total Lipids in Small LDL [%] | 23624 | Statin dose (1 SD) | 0.1177617 | 0.0232151 | 5.07 | 4.02E-07 | 9.16E-05 |
| Statin users | Phospholipids to Total Lipids in Small LDL [%] | 23624 | GRS (1 SD) | -0.132275 | 0.01427295 | -9.27 | 2.10E-20 | 4.79E-18 |
| Statin users | Phospholipids to Total Lipids in Small LDL [%] | 23624 | Adjusted GRS (1 SD) | -0.005248 | 0.01411991 | -0.37 | 7.10E-01 | 1.00E+00 |

| collective | metabolite | field | variable | Estimate | Std. Error | t value | p value | p value Bonferroni |
|---|---|---|---|---|---|---|---|---|
| Non-statin users | Phospholipids to Total Lipids in Small LDL [%] | 23624 | GRS (1 SD) | -0.0881225 | 0.00583476 | -15.10 | 1.80E-51 | 4.09E-49 |
| Non-statin users | Phospholipids to Total Lipids in Small LDL [%] | 23624 | Adjusted GRS (1 SD) | -0.0009017 | 0.00580123 | -0.16 | 8.76E-01 | 1.00E+00 |
| All | Phospholipids to Total Lipids in Small LDL [%] | 23624 | Statin dose (1 SD) | 0.17274197 | 0.02433277 | 7.10 | 1.35E-12 | 3.08E-10 |
| All | Phospholipids to Total Lipids in Small LDL [%] | 23624 | GRS (1 SD) | -0.1010175 | 0.00520476 | -19.41 | 8.83E-84 | 2.01E-81 |
| All | Phospholipids to Total Lipids in Small LDL [%] | 23624 | Adjusted GRS (1 SD) | -0.0039112 | 0.00517534 | -0.76 | 4.50E-01 | 1.00E+00 |
| Statin users | Cholesterol to Total Lipids in Small LDL [%] | 23625 | Statin dose (1 SD) | -0.2065564 | 0.02735394 | -7.55 | 4.83E-14 | 1.10E-11 |
| Statin users | Cholesterol to Total Lipids in Small LDL [%] | 23625 | GRS (1 SD) | -0.0995287 | 0.01724424 | -5.77 | 7.97E-09 | 1.82E-06 |
| Statin users | Cholesterol to Total Lipids in Small LDL [%] | 23625 | Adjusted GRS (1 SD) | -0.016028 | 0.01703594 | -0.94 | 3.47E-01 | 1.00E+00 |
| Non-statin users | Cholesterol to Total Lipids in Small LDL [%] | 23625 | GRS (1 SD) | -0.0600891 | 0.00611551 | -9.83 | 8.97E-23 | 2.04E-20 |
| Non-statin users | Cholesterol to Total Lipids in Small LDL [%] | 23625 | Adjusted GRS (1 SD) | -0.0054134 | 0.00607579 | -0.89 | 3.73E-01 | 1.00E+00 |
| All | Cholesterol to Total Lipids in Small LDL [%] | 23625 | Statin dose (1 SD) | -0.3152426 | 0.02946889 | -10.70 | 1.51E-26 | 3.44E-24 |
| All | Cholesterol to Total Lipids in Small LDL [%] | 23625 | GRS (1 SD) | -0.0491309 | 0.00593108 | -8.28 | 1.21E-16 | 2.75E-14 |
| All | Cholesterol to Total Lipids in Small LDL [%] | 23625 | Adjusted GRS (1 SD) | -0.0010395 | 0.00588983 | -0.18 | 8.60E-01 | 1.00E+00 |
| Statin users | CE to Total Lipids in Small LDL [%] | 23626 | Statin dose (1 SD) | -0.162639 | 0.02672057 | -6.09 | 1.21E-09 | 2.76E-07 |
| Statin users | CE to Total Lipids in Small LDL [%] | 23626 | GRS (1 SD) | 0.1651229 | 0.0166202 | 9.94 | 3.33E-23 | 7.59E-21 |

| collective | metabolite | field | variable | Estimate | Std. Error | t value | p value | p value Bonferroni |
|---|---|---|---|---|---|---|---|---|
| Statin users | CE to Total Lipids in Small LDL [%] | 23626 | Adjusted GRS (1 SD) | 0.0107988 | 0.01644728 | 0.66 | 5.11E-01 | 1.00E+00 |
| Non-statin users | CE to Total Lipids in Small LDL [%] | 23626 | GRS (1 SD) | 0.11224377 | 0.00691543 | 16.23 | 3.72E-59 | 8.48E-57 |
| Non-statin users | CE to Total Lipids in Small LDL [%] | 23626 | Adjusted GRS (1 SD) | 0.00206782 | 0.00687706 | 0.30 | 7.64E-01 | 1.00E+00 |
| All | CE to Total Lipids in Small LDL [%] | 23626 | Statin dose (1 SD) | -0.2560856 | 0.02842261 | -9.01 | 2.48E-19 | 5.67E-17 |
| All | CE to Total Lipids in Small LDL [%] | 23626 | GRS (1 SD) | 0.13065434 | 0.00621235 | 21.03 | 5.15E-98 | 1.17E-95 |
| All | CE to Total Lipids in Small LDL [%] | 23626 | Adjusted GRS (1 SD) | 0.00708597 | 0.00617895 | 1.15 | 2.51E-01 | 1.00E+00 |
| Statin users | FC to Total Lipids in Small LDL [%] | 23627 | Statin dose (1 SD) | -0.043921 | 0.02716302 | -1.62 | 1.06E-01 | 1.00E+00 |
| Statin users | FC to Total Lipids in Small LDL [%] | 23627 | GRS (1 SD) | -0.2646487 | 0.01688246 | -15.68 | 4.81E-55 | 1.10E-52 |
| Statin users | FC to Total Lipids in Small LDL [%] | 23627 | Adjusted GRS (1 SD) | -0.0268228 | 0.01676887 | -1.60 | 1.10E-01 | 1.00E+00 |
| Non-statin users | FC to Total Lipids in Small LDL [%] | 23627 | GRS (1 SD) | -0.1723337 | 0.00615342 | -28.01 | 7.82E-172 | 1.78E-169 |
| Non-statin users | FC to Total Lipids in Small LDL [%] | 23627 | Adjusted GRS (1 SD) | -0.0074816 | 0.00613728 | -1.22 | 2.23E-01 | 1.00E+00 |
| All | FC to Total Lipids in Small LDL [%] | 23627 | Statin dose (1 SD) | -0.059161 | 0.02926413 | -2.02 | 4.32E-02 | 1.00E+00 |
| All | FC to Total Lipids in Small LDL [%] | 23627 | GRS (1 SD) | -0.1797853 | 0.00576687 | -31.18 | 1.71E-212 | 3.89E-210 |
| All | FC to Total Lipids in Small LDL [%] | 23627 | Adjusted GRS (1 SD) | -0.008125 | 0.00574876 | -1.41 | 1.58E-01 | 1.00E+00 |
| Statin users | TG to Total Lipids in Small LDL [%] | 23628 | Statin dose (1 SD) | 0.08880105 | 0.02493681 | 3.56 | 3.72E-04 | 8.48E-02 |
| Statin users | TG to Total Lipids in Small LDL [%] | 23628 | GRS (1 SD) | 0.23180465 | 0.01552707 | 14.93 | 4.05E-50 | 9.24E-48 |
| Statin users | TG to Total Lipids in Small LDL [%] | 23628 | Adjusted GRS (1 SD) | 0.02127649 | 0.01541386 | 1.38 | 1.67E-01 | 1.00E+00 |
| Non-statin users | TG to Total Lipids in Small LDL [%] | 23628 | GRS (1 SD) | 0.14821049 | 0.00544408 | 27.22 | 1.59E-162 | 3.64E-160 |
| Non-statin users | TG to Total Lipids in Small LDL [%] | 23628 | Adjusted GRS (1 SD) | 0.00631379 | 0.00542847 | 1.16 | 2.45E-01 | 1.00E+00 |
| All | TG to Total Lipids in Small LDL [%] | 23628 | Statin dose (1 SD) | 0.14250797 | 0.0269766 | 5.28 | 1.30E-07 | 2.97E-05 |
| All | TG to Total Lipids in Small LDL [%] | 23628 | GRS (1 SD) | 0.15014754 | 0.00526726 | 28.51 | 4.05E-178 | 9.24E-176 |
| All | TG to Total Lipids in Small LDL [%] | 23628 | Adjusted GRS (1 SD) | 0.00494954 | 0.00524719 | 0.94 | 3.46E-01 | 1.00E+00 |
| Statin users | Phospholipids to Total Lipids in Very Large HDL [%] | 23629 | Statin dose (1 SD) | -0.2199407 | 0.0953046 | -2.31 | 2.10E-02 | 1.00E+00 |
| Statin users | Phospholipids to Total Lipids in Very Large HDL [%] | 23629 | GRS (1 SD) | -0.150604 | 0.05964319 | -2.53 | 1.16E-02 | 1.00E+00 |
| Statin users | Phospholipids to Total Lipids in Very Large HDL [%] | 23629 | Adjusted GRS (1 SD) | -0.0263059 | 0.05889826 | -0.45 | 6.55E-01 | 1.00E+00 |

| collective | metabolite | field | variable | Estimate | Std. Error | t value | p value | p value Bonferroni |
|---|---|---|---|---|---|---|---|---|
| Non-statin users | Phospholipids to Total Lipids in Very Large HDL [%] | 23629 | GRS (1 SD) | 0.01109413 | 0.01778444 | 0.62 | 5.33E-01 | 1.00E+00 |
| Non-statin users | Phospholipids to Total Lipids in Very Large HDL [%] | 23629 | Adjusted GRS (1 SD) | 0.01321327 | 0.01765916 | 0.75 | 4.54E-01 | 1.00E+00 |
| All | Phospholipids to Total Lipids in Very Large HDL [%] | 23629 | Statin dose (1 SD) | -0.3021213 | 0.09812429 | -3.08 | 2.08E-03 | 4.75E-01 |
| All | Phospholipids to Total Lipids in Very Large HDL [%] | 23629 | GRS (1 SD) | 0.00465079 | 0.017337 | 0.27 | 7.89E-01 | 1.00E+00 |
| All | Phospholipids to Total Lipids in Very Large HDL [%] | 23629 | Adjusted GRS (1 SD) | 0.01161123 | 0.01721194 | 0.67 | 5.00E-01 | 1.00E+00 |
| Statin users | Cholesterol to Total Lipids in Very Large HDL [%] | 23630 | Statin dose (1 SD) | -0.0157818 | 0.06962713 | -0.23 | 8.21E-01 | 1.00E+00 |
| Statin users | Cholesterol to Total Lipids in Very Large HDL [%] | 23630 | GRS (1 SD) | -0.2308305 | 0.04323887 | -5.34 | 9.48E-08 | 2.16E-05 |
| Statin users | Cholesterol to Total Lipids in Very Large HDL [%] | 23630 | Adjusted GRS (1 SD) | -0.0133701 | 0.04272333 | -0.31 | 7.54E-01 | 1.00E+00 |
| Non-statin users | Cholesterol to Total Lipids in Very Large HDL [%] | 23630 | GRS (1 SD) | -0.1500481 | 0.01390947 | -10.79 | 4.10E-27 | 9.35E-25 |
| Non-statin users | Cholesterol to Total Lipids in Very Large HDL [%] | 23630 | Adjusted GRS (1 SD) | -0.0146914 | 0.01382055 | -1.06 | 2.88E-01 | 1.00E+00 |
| All | Cholesterol to Total Lipids in Very Large HDL [%] | 23630 | Statin dose (1 SD) | 0.02185308 | 0.07245489 | 0.30 | 7.63E-01 | 1.00E+00 |
| All | Cholesterol to Total Lipids in Very Large HDL [%] | 23630 | GRS (1 SD) | -0.1709075 | 0.01318926 | -12.96 | 2.25E-38 | 5.12E-36 |
| All | Cholesterol to Total Lipids in Very Large HDL [%] | 23630 | Adjusted GRS (1 SD) | -0.0157693 | 0.01310345 | -1.20 | 2.29E-01 | 1.00E+00 |
| Statin users | CE to Total Lipids in Very Large HDL [%] | 23631 | Statin dose (1 SD) | -0.1527777 | 0.0500831 | -3.05 | 2.29E-03 | 5.23E-01 |
| Statin users | CE to Total Lipids in Very Large HDL [%] | 23631 | GRS (1 SD) | -0.3167328 | 0.03192043 | -9.92 | 3.77E-23 | 8.60E-21 |

| collective | metabolite | field | variable | Estimate | Std. Error | t value | p value | p value Bonferroni |
|---|---|---|---|---|---|---|---|---|
| Statin users | CE to Total Lipids in Very Large HDL [%] | 23631 | Adjusted GRS (1 SD) | -0.0401123 | 0.03159548 | -1.27 | 2.04E-01 | 1.00E+00 |
| Non-statin users | CE to Total Lipids in Very Large HDL [%] | 23631 | GRS (1 SD) | -0.1623899 | 0.00970619 | -16.73 | 9.82E-63 | 2.24E-60 |
| Non-statin users | CE to Total Lipids in Very Large HDL [%] | 23631 | Adjusted GRS (1 SD) | -0.0127907 | 0.00965306 | -1.33 | 1.85E-01 | 1.00E+00 |
| All | CE to Total Lipids in Very Large HDL [%] | 23631 | Statin dose (1 SD) | -0.1796796 | 0.0525298 | -3.42 | 6.28E-04 | 1.43E-01 |
| All | CE to Total Lipids in Very Large HDL [%] | 23631 | GRS (1 SD) | -0.1807653 | 0.0094684 | -19.09 | 3.96E-81 | 9.02E-79 |
| All | CE to Total Lipids in Very Large HDL [%] | 23631 | Adjusted GRS (1 SD) | -0.0146306 | 0.00941465 | -1.55 | 1.20E-01 | 1.00E+00 |
| Statin users | FC to Total Lipids in Very Large HDL [%] | 23632 | Statin dose (1 SD) | 0.13699866 | 0.05360327 | 2.56 | 1.06E-02 | 1.00E+00 |
| Statin users | FC to Total Lipids in Very Large HDL [%] | 23632 | GRS (1 SD) | 0.08590542 | 0.03330493 | 2.58 | 9.91E-03 | 1.00E+00 |
| Statin users | FC to Total Lipids in Very Large HDL [%] | 23632 | Adjusted GRS (1 SD) | 0.02674445 | 0.03288879 | 0.81 | 4.16E-01 | 1.00E+00 |
| Non-statin users | FC to Total Lipids in Very Large HDL [%] | 23632 | GRS (1 SD) | 0.01234058 | 0.00978485 | 1.26 | 2.07E-01 | 1.00E+00 |
| Non-statin users | FC to Total Lipids in Very Large HDL [%] | 23632 | Adjusted GRS (1 SD) | -0.0019015 | 0.00971602 | -0.20 | 8.45E-01 | 1.00E+00 |
| All | FC to Total Lipids in Very Large HDL [%] | 23632 | Statin dose (1 SD) | 0.20153494 | 0.05497005 | 3.67 | 2.48E-04 | 5.64E-02 |
| All | FC to Total Lipids in Very Large HDL [%] | 23632 | GRS (1 SD) | 0.00985704 | 0.00960152 | 1.03 | 3.05E-01 | 1.00E+00 |
| All | FC to Total Lipids in Very Large HDL [%] | 23632 | Adjusted GRS (1 SD) | -0.0011398 | 0.00953232 | -0.12 | 9.05E-01 | 1.00E+00 |
| Statin users | TG to Total Lipids in Very Large HDL [%] | 23633 | Statin dose (1 SD) | 0.23571495 | 0.06023024 | 3.91 | 9.18E-05 | 2.09E-02 |
| Statin users | TG to Total Lipids in Very Large HDL [%] | 23633 | GRS (1 SD) | 0.38143172 | 0.03859007 | 9.88 | 5.53E-23 | 1.26E-20 |
| Statin users | TG to Total Lipids in Very Large HDL [%] | 23633 | Adjusted GRS (1 SD) | 0.0396739 | 0.038197 | 1.04 | 2.99E-01 | 1.00E+00 |
| Non-statin users | TG to Total Lipids in Very Large HDL [%] | 23633 | GRS (1 SD) | 0.13895508 | 0.00935785 | 14.85 | 8.11E-50 | 1.85E-47 |
| Non-statin users | TG to Total Lipids in Very Large HDL [%] | 23633 | Adjusted GRS (1 SD) | 0.00147881 | 0.00930358 | 0.16 | 8.74E-01 | 1.00E+00 |
| All | TG to Total Lipids in Very Large HDL [%] | 23633 | Statin dose (1 SD) | 0.28026155 | 0.06203236 | 4.52 | 6.32E-06 | 1.44E-03 |
| All | TG to Total Lipids in Very Large HDL [%] | 23633 | GRS (1 SD) | 0.16625698 | 0.01001354 | 16.60 | 7.78E-62 | 1.77E-59 |
| All | TG to Total Lipids in Very Large HDL [%] | 23633 | Adjusted GRS (1 SD) | 0.00415837 | 0.00995302 | 0.42 | 6.76E-01 | 1.00E+00 |
| Statin users | Phospholipids to Total Lipids in Large HDL [%] | 23634 | Statin dose (1 SD) | 0.11212045 | 0.04072117 | 2.75 | 5.91E-03 | 1.00E+00 |
| Statin users | Phospholipids to Total Lipids in Large HDL [%] | 23634 | GRS (1 SD) | 0.18174229 | 0.02617172 | 6.94 | 3.93E-12 | 8.95E-10 |
| Statin users | Phospholipids to Total Lipids in Large HDL [%] | 23634 | Adjusted GRS (1 SD) | 0.01568363 | 0.02586587 | 0.61 | 5.44E-01 | 1.00E+00 |

| collective | metabolite | field | variable | Estimate | Std. Error | t value | p value | p value Bonferroni |
|---|---|---|---|---|---|---|---|---|
| Non-statin users | Phospholipids to Total Lipids in Large HDL [%] | 23634 | GRS (1 SD) | 0.08655238 | 0.00839393 | 10.31 | 6.47E-25 | 1.48E-22 |
| Non-statin users | Phospholipids to Total Lipids in Large HDL [%] | 23634 | Adjusted GRS (1 SD) | 0.00080245 | 0.00833992 | 0.10 | 9.23E-01 | 1.00E+00 |
| All | Phospholipids to Total Lipids in Large HDL [%] | 23634 | Statin dose (1 SD) | 0.28201057 | 0.04463726 | 6.32 | 2.78E-10 | 6.34E-08 |
| All | Phospholipids to Total Lipids in Large HDL [%] | 23634 | GRS (1 SD) | 0.07537403 | 0.00857103 | 8.79 | 1.46E-18 | 3.33E-16 |
| All | Phospholipids to Total Lipids in Large HDL [%] | 23634 | Adjusted GRS (1 SD) | -0.002513 | 0.00851174 | -0.30 | 7.68E-01 | 1.00E+00 |
| Statin users | Cholesterol to Total Lipids in Large HDL [%] | 23635 | Statin dose (1 SD) | -0.294482 | 0.07611897 | -3.87 | 1.10E-04 | 2.52E-02 |
| Statin users | Cholesterol to Total Lipids in Large HDL [%] | 23635 | GRS (1 SD) | -0.4112587 | 0.04826362 | -8.52 | 1.69E-17 | 3.86E-15 |
| Statin users | Cholesterol to Total Lipids in Large HDL [%] | 23635 | Adjusted GRS (1 SD) | -0.0508979 | 0.04772859 | -1.07 | 2.86E-01 | 1.00E+00 |
| Non-statin users | Cholesterol to Total Lipids in Large HDL [%] | 23635 | GRS (1 SD) | -0.2296951 | 0.01536334 | -14.95 | 1.77E-50 | 4.04E-48 |
| Non-statin users | Cholesterol to Total Lipids in Large HDL [%] | 23635 | Adjusted GRS (1 SD) | -0.0068451 | 0.01527463 | -0.45 | 6.54E-01 | 1.00E+00 |
| All | Cholesterol to Total Lipids in Large HDL [%] | 23635 | Statin dose (1 SD) | -0.4488724 | 0.08074741 | -5.56 | 2.79E-08 | 6.36E-06 |
| All | Cholesterol to Total Lipids in Large HDL [%] | 23635 | GRS (1 SD) | -0.2272497 | 0.01523612 | -14.92 | 2.92E-50 | 6.66E-48 |
| All | Cholesterol to Total Lipids in Large HDL [%] | 23635 | Adjusted GRS (1 SD) | -0.0045225 | 0.01514009 | -0.30 | 7.65E-01 | 1.00E+00 |
| Statin users | CE to Total Lipids in Large HDL [%] | 23636 | Statin dose (1 SD) | -0.2091741 | 0.06445981 | -3.25 | 1.18E-03 | 2.69E-01 |
| Statin users | CE to Total Lipids in Large HDL [%] | 23636 | GRS (1 SD) | -0.3261335 | 0.04083898 | -7.99 | 1.47E-15 | 3.36E-13 |

| collective | metabolite | field | variable | Estimate | Std. Error | t value | p value | p value Bonferroni |
|---|---|---|---|---|---|---|---|---|
| Statin users | CE to Total Lipids in Large HDL [%] | 23636 | Adjusted GRS (1 SD) | -0.0462601 | 0.04037689 | -1.15 | 2.52E-01 | 1.00E+00 |
| Non-statin users | CE to Total Lipids in Large HDL [%] | 23636 | GRS (1 SD) | -0.1590943 | 0.01370521 | -11.61 | 3.94E-31 | 8.98E-29 |
| Non-statin users | CE to Total Lipids in Large HDL [%] | 23636 | Adjusted GRS (1 SD) | -0.0085331 | 0.0136192 | -0.63 | 5.31E-01 | 1.00E+00 |
| All | CE to Total Lipids in Large HDL [%] | 23636 | Statin dose (1 SD) | -0.290149 | 0.06863225 | -4.23 | 2.39E-05 | 5.44E-03 |
| All | CE to Total Lipids in Large HDL [%] | 23636 | GRS (1 SD) | -0.1632175 | 0.01328995 | -12.28 | 1.20E-34 | 2.73E-32 |
| All | CE to Total Lipids in Large HDL [%] | 23636 | Adjusted GRS (1 SD) | -0.0080493 | 0.01320213 | -0.61 | 5.42E-01 | 1.00E+00 |
| Statin users | FC to Total Lipids in Large HDL [%] | 23637 | Statin dose (1 SD) | -0.0853034 | 0.01694352 | -5.03 | 4.90E-07 | 1.12E-04 |
| Statin users | FC to Total Lipids in Large HDL [%] | 23637 | GRS (1 SD) | -0.0851277 | 0.01067971 | -7.97 | 1.66E-15 | 3.79E-13 |
| Statin users | FC to Total Lipids in Large HDL [%] | 23637 | Adjusted GRS (1 SD) | -0.0046381 | 0.01055911 | -0.44 | 6.60E-01 | 1.00E+00 |
| Non-statin users | FC to Total Lipids in Large HDL [%] | 23637 | GRS (1 SD) | -0.0706001 | 0.00306538 | -23.03 | 5.02E-117 | 1.15E-114 |
| Non-statin users | FC to Total Lipids in Large HDL [%] | 23637 | Adjusted GRS (1 SD) | 0.00168861 | 0.00305298 | 0.55 | 5.80E-01 | 1.00E+00 |
| All | FC to Total Lipids in Large HDL [%] | 23637 | Statin dose (1 SD) | -0.1587182 | 0.0182016 | -8.72 | 3.28E-18 | 7.47E-16 |
| All | FC to Total Lipids in Large HDL [%] | 23637 | GRS (1 SD) | -0.0640324 | 0.00320672 | -19.97 | 1.46E-88 | 3.34E-86 |
| All | FC to Total Lipids in Large HDL [%] | 23637 | Adjusted GRS (1 SD) | 0.00352683 | 0.00318889 | 1.11 | 2.69E-01 | 1.00E+00 |
| Statin users | TG to Total Lipids in Large HDL [%] | 23638 | Statin dose (1 SD) | 0.18236122 | 0.04186387 | 4.36 | 1.34E-05 | 3.06E-03 |
| Statin users | TG to Total Lipids in Large HDL [%] | 23638 | GRS (1 SD) | 0.22951615 | 0.02769442 | 8.29 | 1.23E-16 | 2.81E-14 |
| Statin users | TG to Total Lipids in Large HDL [%] | 23638 | Adjusted GRS (1 SD) | 0.03521437 | 0.02738428 | 1.29 | 1.98E-01 | 1.00E+00 |
| Non-statin users | TG to Total Lipids in Large HDL [%] | 23638 | GRS (1 SD) | 0.14314396 | 0.00974132 | 14.69 | 7.99E-49 | 1.82E-46 |
| Non-statin users | TG to Total Lipids in Large HDL [%] | 23638 | Adjusted GRS (1 SD) | 0.00604302 | 0.00968464 | 0.62 | 5.33E-01 | 1.00E+00 |
| All | TG to Total Lipids in Large HDL [%] | 23638 | Statin dose (1 SD) | 0.16686345 | 0.04600002 | 3.63 | 2.88E-04 | 6.56E-02 |
| All | TG to Total Lipids in Large HDL [%] | 23638 | GRS (1 SD) | 0.15187592 | 0.00918155 | 16.54 | 2.17E-61 | 4.94E-59 |
| All | TG to Total Lipids in Large HDL [%] | 23638 | Adjusted GRS (1 SD) | 0.00703554 | 0.00912565 | 0.77 | 4.41E-01 | 1.00E+00 |
| Statin users | Phospholipids to Total Lipids in Medium HDL [%] | 23639 | Statin dose (1 SD) | 0.01976001 | 0.01471746 | 1.34 | 1.79E-01 | 1.00E+00 |
| Statin users | Phospholipids to Total Lipids in Medium HDL [%] | 23639 | GRS (1 SD) | 0.06134735 | 0.0093486 | 6.56 | 5.44E-11 | 1.24E-08 |
| Statin users | Phospholipids to Total Lipids in Medium HDL [%] | 23639 | Adjusted GRS (1 SD) | 0.01219351 | 0.0092378 | 1.32 | 1.87E-01 | 1.00E+00 |

| collective | metabolite | field | variable | Estimate | Std. Error | t value | p value | p value Bonferroni |
|---|---|---|---|---|---|---|---|---|
| Non-statin users | Phospholipids to Total Lipids in Medium HDL [%] | 23639 | GRS (1 SD) | 0.0409253 | 0.00402294 | 10.17 | 2.70E-24 | 6.16E-22 |
| Non-statin users | Phospholipids to Total Lipids in Medium HDL [%] | 23639 | Adjusted GRS (1 SD) | 0.00426883 | 0.00399696 | 1.07 | 2.86E-01 | 1.00E+00 |
| All | Phospholipids to Total Lipids in Medium HDL [%] | 23639 | Statin dose (1 SD) | 0.02609789 | 0.01591025 | 1.64 | 1.01E-01 | 1.00E+00 |
| All | Phospholipids to Total Lipids in Medium HDL [%] | 23639 | GRS (1 SD) | 0.04000675 | 0.00361006 | 11.08 | 1.58E-28 | 3.61E-26 |
| All | Phospholipids to Total Lipids in Medium HDL [%] | 23639 | Adjusted GRS (1 SD) | 0.00417325 | 0.00358576 | 1.16 | 2.44E-01 | 1.00E+00 |
| Statin users | Cholesterol to Total Lipids in Medium HDL [%] | 23640 | Statin dose (1 SD) | -0.0509894 | 0.03372841 | -1.51 | 1.31E-01 | 1.00E+00 |
| Statin users | Cholesterol to Total Lipids in Medium HDL [%] | 23640 | GRS (1 SD) | -0.1699948 | 0.02125921 | -8.00 | 1.35E-15 | 3.09E-13 |
| Statin users | Cholesterol to Total Lipids in Medium HDL [%] | 23640 | Adjusted GRS (1 SD) | -0.0386908 | 0.02101763 | -1.84 | 6.57E-02 | 1.00E+00 |
| Non-statin users | Cholesterol to Total Lipids in Medium HDL [%] | 23640 | GRS (1 SD) | -0.1073614 | 0.0095006 | -11.30 | 1.37E-29 | 3.12E-27 |
| Non-statin users | Cholesterol to Total Lipids in Medium HDL [%] | 23640 | Adjusted GRS (1 SD) | -0.0091569 | 0.00944056 | -0.97 | 3.32E-01 | 1.00E+00 |
| All | Cholesterol to Total Lipids in Medium HDL [%] | 23640 | Statin dose (1 SD) | -0.0199891 | 0.037189 | -0.54 | 5.91E-01 | 1.00E+00 |
| All | Cholesterol to Total Lipids in Medium HDL [%] | 23640 | GRS (1 SD) | -0.1128439 | 0.00845601 | -13.34 | 1.36E-40 | 3.10E-38 |
| All | Cholesterol to Total Lipids in Medium HDL [%] | 23640 | Adjusted GRS (1 SD) | -0.0123307 | 0.00840104 | -1.47 | 1.42E-01 | 1.00E+00 |
| Statin users | CE to Total Lipids in Medium HDL [%] | 23641 | Statin dose (1 SD) | -0.01969 | 0.02979332 | -0.66 | 5.09E-01 | 1.00E+00 |
| Statin users | CE to Total Lipids in Medium HDL [%] | 23641 | GRS (1 SD) | -0.1438682 | 0.01873919 | -7.68 | 1.70E-14 | 3.88E-12 |

| collective | metabolite | field | variable | Estimate | Std. Error | t value | p value | p value Bonferroni |
|---|---|---|---|---|---|---|---|---|
| Statin users | CE to Total Lipids in Medium HDL [%] | 23641 | Adjusted GRS (1 SD) | -0.0327533 | 0.01852399 | -1.77 | 7.71E-02 | 1.00E+00 |
| Non-statin users | CE to Total Lipids in Medium HDL [%] | 23641 | GRS (1 SD) | -0.0799825 | 0.00836062 | -9.57 | 1.13E-21 | 2.58E-19 |
| Non-statin users | CE to Total Lipids in Medium HDL [%] | 23641 | Adjusted GRS (1 SD) | -0.008795 | 0.00830608 | -1.06 | 2.90E-01 | 1.00E+00 |
| All | CE to Total Lipids in Medium HDL [%] | 23641 | Statin dose (1 SD) | 0.03637807 | 0.03318091 | 1.10 | 2.73E-01 | 1.00E+00 |
| All | CE to Total Lipids in Medium HDL [%] | 23641 | GRS (1 SD) | -0.0891319 | 0.00744947 | -11.96 | 5.68E-33 | 1.29E-30 |
| All | CE to Total Lipids in Medium HDL [%] | 23641 | Adjusted GRS (1 SD) | -0.012355 | 0.00739993 | -1.67 | 9.50E-02 | 1.00E+00 |
| Statin users | FC to Total Lipids in Medium HDL [%] | 23642 | Statin dose (1 SD) | -0.0312964 | 0.00747426 | -4.19 | 2.86E-05 | 6.51E-03 |
| Statin users | FC to Total Lipids in Medium HDL [%] | 23642 | GRS (1 SD) | -0.0261264 | 0.00477513 | -5.47 | 4.52E-08 | 1.03E-05 |
| Statin users | FC to Total Lipids in Medium HDL [%] | 23642 | Adjusted GRS (1 SD) | -0.0059369 | 0.00471695 | -1.26 | 2.08E-01 | 1.00E+00 |
| Non-statin users | FC to Total Lipids in Medium HDL [%] | 23642 | GRS (1 SD) | -0.0273788 | 0.00208119 | -13.16 | 1.73E-39 | 3.94E-37 |
| Non-statin users | FC to Total Lipids in Medium HDL [%] | 23642 | Adjusted GRS (1 SD) | -0.0003608 | 0.00206858 | -0.17 | 8.62E-01 | 1.00E+00 |
| All | FC to Total Lipids in Medium HDL [%] | 23642 | Statin dose (1 SD) | -0.0563636 | 0.00808726 | -6.97 | 3.41E-12 | 7.77E-10 |
| All | FC to Total Lipids in Medium HDL [%] | 23642 | GRS (1 SD) | -0.0237118 | 0.00187422 | -12.65 | 1.16E-36 | 2.64E-34 |
| All | FC to Total Lipids in Medium HDL [%] | 23642 | Adjusted GRS (1 SD) | 2.5632E-05 | 0.00186191 | 0.01 | 9.89E-01 | 1.00E+00 |
| Statin users | TG to Total Lipids in Medium HDL [%] | 23643 | Statin dose (1 SD) | 0.03122012 | 0.02037985 | 1.53 | 1.26E-01 | 1.00E+00 |
| Statin users | TG to Total Lipids in Medium HDL [%] | 23643 | GRS (1 SD) | 0.10864495 | 0.01279784 | 8.49 | 2.23E-17 | 5.08E-15 |
| Statin users | TG to Total Lipids in Medium HDL [%] | 23643 | Adjusted GRS (1 SD) | 0.02649288 | 0.01265473 | 2.09 | 3.63E-02 | 1.00E+00 |
| Non-statin users | TG to Total Lipids in Medium HDL [%] | 23643 | GRS (1 SD) | 0.06643903 | 0.00601911 | 11.04 | 2.61E-28 | 5.96E-26 |
| Non-statin users | TG to Total Lipids in Medium HDL [%] | 23643 | Adjusted GRS (1 SD) | 0.00488998 | 0.00598089 | 0.82 | 4.14E-01 | 1.00E+00 |
| All | TG to Total Lipids in Medium HDL [%] | 23643 | Statin dose (1 SD) | -0.0061184 | 0.02296062 | -0.27 | 7.90E-01 | 1.00E+00 |
| All | TG to Total Lipids in Medium HDL [%] | 23643 | GRS (1 SD) | 0.07283865 | 0.00530479 | 13.73 | 7.18E-43 | 1.64E-40 |
| All | TG to Total Lipids in Medium HDL [%] | 23643 | Adjusted GRS (1 SD) | 0.0081578 | 0.00527054 | 1.55 | 1.22E-01 | 1.00E+00 |
| Statin users | Phospholipids to Total Lipids in Small HDL [%] | 23644 | Statin dose (1 SD) | -0.0233948 | 0.01354495 | -1.73 | 8.42E-02 | 1.00E+00 |
| Statin users | Phospholipids to Total Lipids in Small HDL [%] | 23644 | GRS (1 SD) | 0.07112963 | 0.00850487 | 8.36 | 6.50E-17 | 1.48E-14 |
| Statin users | Phospholipids to Total Lipids in Small HDL [%] | 23644 | Adjusted GRS (1 SD) | 0.00217982 | 0.00841024 | 0.26 | 7.95E-01 | 1.00E+00 |

| collective | metabolite | field | variable | Estimate | Std. Error | t value | p value | p value Bonferroni |
|---|---|---|---|---|---|---|---|---|
| Non-statin users | Phospholipids to Total Lipids in Small HDL [%] | 23644 | GRS (1 SD) | 0.083629 | 0.00401217 | 20.84 | 2.97E-96 | 6.76E-94 |
| Non-statin users | Phospholipids to Total Lipids in Small HDL [%] | 23644 | Adjusted GRS (1 SD) | 0.00348581 | 0.00399375 | 0.87 | 3.83E-01 | 1.00E+00 |
| All | Phospholipids to Total Lipids in Small HDL [%] | 23644 | Statin dose (1 SD) | 0.00815481 | 0.0150715 | 0.54 | 5.88E-01 | 1.00E+00 |
| All | Phospholipids to Total Lipids in Small HDL [%] | 23644 | GRS (1 SD) | 0.07710558 | 0.00351357 | 21.95 | 1.58E-106 | 3.61E-104 |
| All | Phospholipids to Total Lipids in Small HDL [%] | 23644 | Adjusted GRS (1 SD) | 0.00202486 | 0.00349527 | 0.58 | 5.62E-01 | 1.00E+00 |
| Statin users | Cholesterol to Total Lipids in Small HDL [%] | 23645 | Statin dose (1 SD) | -0.0146509 | 0.02128415 | -0.69 | 4.91E-01 | 1.00E+00 |
| Statin users | Cholesterol to Total Lipids in Small HDL [%] | 23645 | GRS (1 SD) | -0.1478379 | 0.01342052 | -11.02 | 3.89E-28 | 8.86E-26 |
| Statin users | Cholesterol to Total Lipids in Small HDL [%] | 23645 | Adjusted GRS (1 SD) | -0.0216883 | 0.01328784 | -1.63 | 1.03E-01 | 1.00E+00 |
| Non-statin users | Cholesterol to Total Lipids in Small HDL [%] | 23645 | GRS (1 SD) | -0.130231 | 0.00610514 | -21.33 | 1.05E-100 | 2.39E-98 |
| Non-statin users | Cholesterol to Total Lipids in Small HDL [%] | 23645 | Adjusted GRS (1 SD) | -0.0077469 | 0.0060778 | -1.27 | 2.02E-01 | 1.00E+00 |
| All | Cholesterol to Total Lipids in Small HDL [%] | 23645 | Statin dose (1 SD) | -0.0283168 | 0.02361613 | -1.20 | 2.31E-01 | 1.00E+00 |
| All | Cholesterol to Total Lipids in Small HDL [%] | 23645 | GRS (1 SD) | -0.1269717 | 0.00541436 | -23.45 | 2.47E-121 | 5.63E-119 |
| All | Cholesterol to Total Lipids in Small HDL [%] | 23645 | Adjusted GRS (1 SD) | -0.0083607 | 0.00538768 | -1.55 | 1.21E-01 | 1.00E+00 |
| Statin users | CE to Total Lipids in Small HDL [%] | 23646 | Statin dose (1 SD) | -0.0203534 | 0.02112428 | -0.96 | 3.35E-01 | 1.00E+00 |
| Statin users | CE to Total Lipids in Small HDL [%] | 23646 | GRS (1 SD) | -0.1206423 | 0.01328249 | -9.08 | 1.16E-19 | 2.64E-17 |

| collective | metabolite | field | variable | Estimate | Std. Error | t value | p value | p value Bonferroni |
|---|---|---|---|---|---|---|---|---|
| Statin users | CE to Total Lipids in Small HDL [%] | 23646 | Adjusted GRS (1 SD) | -0.0232506 | 0.01313791 | -1.77 | 7.68E-02 | 1.00E+00 |
| Non-statin users | CE to Total Lipids in Small HDL [%] | 23646 | GRS (1 SD) | -0.0992954 | 0.00614597 | -16.16 | 1.25E-58 | 2.85E-56 |
| Non-statin users | CE to Total Lipids in Small HDL [%] | 23646 | Adjusted GRS (1 SD) | -0.0081152 | 0.00611173 | -1.33 | 1.84E-01 | 1.00E+00 |
| All | CE to Total Lipids in Small HDL [%] | 23646 | Statin dose (1 SD) | -0.0041142 | 0.02358964 | -0.17 | 8.62E-01 | 1.00E+00 |
| All | CE to Total Lipids in Small HDL [%] | 23646 | GRS (1 SD) | -0.1001125 | 0.00540466 | -18.52 | 1.72E-76 | 3.93E-74 |
| All | CE to Total Lipids in Small HDL [%] | 23646 | Adjusted GRS (1 SD) | -0.0100632 | 0.00537327 | -1.87 | 6.11E-02 | 1.00E+00 |
| Statin users | FC to Total Lipids in Small HDL [%] | 23647 | Statin dose (1 SD) | 0.00569912 | 0.00533998 | 1.07 | 2.86E-01 | 1.00E+00 |
| Statin users | FC to Total Lipids in Small HDL [%] | 23647 | GRS (1 SD) | -0.0272021 | 0.00336372 | -8.09 | 6.48E-16 | 1.48E-13 |
| Statin users | FC to Total Lipids in Small HDL [%] | 23647 | Adjusted GRS (1 SD) | 0.00155715 | 0.00332589 | 0.47 | 6.40E-01 | 1.00E+00 |
| Non-statin users | FC to Total Lipids in Small HDL [%] | 23647 | GRS (1 SD) | -0.030932 | 0.00171397 | -18.05 | 1.13E-72 | 2.57E-70 |
| Non-statin users | FC to Total Lipids in Small HDL [%] | 23647 | Adjusted GRS (1 SD) | 0.00037154 | 0.00170506 | 0.22 | 8.28E-01 | 1.00E+00 |
| All | FC to Total Lipids in Small HDL [%] | 23647 | Statin dose (1 SD) | -0.0242075 | 0.00622758 | -3.89 | 1.02E-04 | 2.33E-02 |
| All | FC to Total Lipids in Small HDL [%] | 23647 | GRS (1 SD) | -0.0268576 | 0.00151856 | -17.69 | 6.61E-70 | 1.51E-67 |
| All | FC to Total Lipids in Small HDL [%] | 23647 | Adjusted GRS (1 SD) | 0.00170405 | 0.00150957 | 1.13 | 2.59E-01 | 1.00E+00 |
| Statin users | TG to Total Lipids in Small HDL [%] | 23648 | Statin dose (1 SD) | 0.03804499 | 0.01434195 | 2.65 | 8.00E-03 | 1.00E+00 |
| Statin users | TG to Total Lipids in Small HDL [%] | 23648 | GRS (1 SD) | 0.07671358 | 0.0090434 | 8.48 | 2.35E-17 | 5.37E-15 |
| Statin users | TG to Total Lipids in Small HDL [%] | 23648 | Adjusted GRS (1 SD) | 0.01951399 | 0.00894216 | 2.18 | 2.91E-02 | 1.00E+00 |
| Non-statin users | TG to Total Lipids in Small HDL [%] | 23648 | GRS (1 SD) | 0.0465998 | 0.00421239 | 11.06 | 1.99E-28 | 4.54E-26 |
| Non-statin users | TG to Total Lipids in Small HDL [%] | 23648 | Adjusted GRS (1 SD) | 0.00425881 | 0.00418564 | 1.02 | 3.09E-01 | 1.00E+00 |
| All | TG to Total Lipids in Small HDL [%] | 23648 | Statin dose (1 SD) | 0.02016154 | 0.01604655 | 1.26 | 2.09E-01 | 1.00E+00 |
| All | TG to Total Lipids in Small HDL [%] | 23648 | GRS (1 SD) | 0.04986494 | 0.00372792 | 13.38 | 8.94E-41 | 2.04E-38 |
| All | TG to Total Lipids in Small HDL [%] | 23648 | Adjusted GRS (1 SD) | 0.00633475 | 0.00370369 | 1.71 | 8.72E-02 | 1.00E+00 |
